# Targeted inhibition of WIP1 and histone H3K27 demethylase activity synergistically suppresses neuroblastoma growth

**DOI:** 10.1038/s41419-025-07658-1

**Published:** 2025-04-19

**Authors:** Diana Treis, Kristina Ihrmark Lundberg, Nicola Bell, Panagiotis Alkinoos Polychronopoulos, Conny Tümmler, Emma Åkerlund, Stefania Aliverti, Ingrid Lilienthal, Adena Pepich, Brinton Seashore-Ludlow, Kazuyasu Sakaguchi, Per Kogner, John Inge Johnsen, Malin Wickström

**Affiliations:** 1 Childhood Cancer Research Unit, Division of Pediatric Oncology and Surgery, Dept. of Women’s and Children’s Health, Karolinska Institutet, Stockholm, Sweden; 2 Science for Life Laboratory, Dept. of Oncology-Pathology, Karolinska Institutet, Stockholm, Sweden; 3 Laboratory of Biological Chemistry, Dept. of Chemistry, Faculty of Science, Hokkaido University, Sapporo, Japan

**Keywords:** Paediatric cancer, Embryonal neoplasms, Tumour-suppressor proteins, Preclinical research, Drug screening

## Abstract

High-risk neuroblastoma frequently exhibits segmental gain of chromosome 17q, including the locus of *PPM1D*, which encodes the phosphatase WIP1, a regulator of p53 activity, DNA repair, and apoptosis. High expression of *PPM1D* is correlated to poor prognosis, and genetic or pharmacologic inhibition of WIP1 suppresses neuroblastoma growth. Here, we show that combining drugs that target WIP1 and H3K27 demethylation induces synergistic cytotoxicity in neuroblastoma. We screened 527 different compounds together with inhibitors of WIP1 and identified a strong cytotoxic synergism between the WIP1 inhibitor SL-176 and GSK-J4, a specific inhibitor of the H3K27 demethylase JMJD3. Viability assays in neuroblastoma cell lines and treatment of tumor spheroids confirmed the synergistic effect of combining SL-176 with GSK-J4. Immunoblot experiments demonstrated a marked effect on WIP1 downstream targets and apoptosis markers, while qPCR showed a synergistic upregulation of p53 downstream targets PUMA and p21. RNA sequencing revealed a vast number of differentially expressed genes, suggesting a pervasive effect of this drug combination on transcription, with enrichment of pathways involved in DNA damage response. Finally, this drug combination was confirmed to reduce tumor growth in zebrafish xenograft experiments. In conclusion, the combination of the WIP1 inhibitor SL-176 and the epigenetic modifier GSK-J4 induces synergistic cytotoxicity in neuroblastoma cells by potentiating p53 downstream effects.

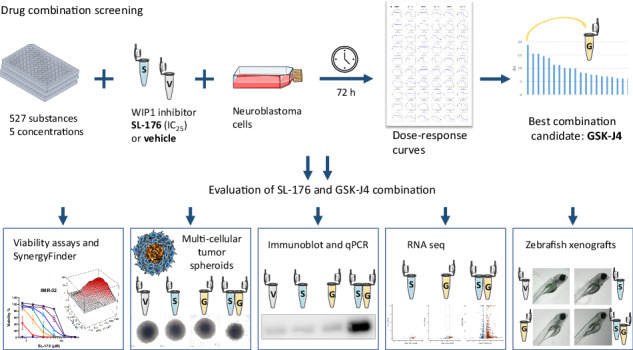

## Introduction

High-risk neuroblastoma remains a deadly disease with a survival rate of only 50-60% [1]. The prognosis is particularly poor in children who relapse after chemotherapy. In light of this, increasing attention has been directed toward the development of targeted therapies for neuroblastoma.

The phosphatase WIP1 (wildtype p53-induced phosphatase 1), encoded by *PPM1D* located on chromosome 17q23.2, is a putative oncoprotein implicated in many malignancies, including neuroblastoma where gain of 17q is a common genetic aberration and a marker of poor prognosis [2–4]. WIP1 halts DNA damage response by directly dephosphorylating p53, inhibiting p53-activated kinases, stabilizing the p53 inhibitor MDM2, and by promoting apoptosis through dephosphorylation of BAX [5, 6]. We and others have demonstrated that inhibition of WIP1 has promising anti-tumorigenic effects in preclinical models of neuroblastoma [4, 7–9]. However, single-drug treatments of malignancies have frequently been observed to induce treatment resistance and tumor regrowth, for instance, by activation of compensatory signaling pathways through genetic and epigenetic changes. Effective and sustained anti-cancer treatment usually relies on combinations of drugs that selectively inhibit multiple targets in cancer cells [10]. Therefore, we performed a drug combination screen using 527 clinical and experimental compounds in combination with the WIP1 inhibitor SL-176 [11]. This screening suggested the epigenetic modulator GSK-J4 as the most promising combination partner.

The small molecule GSK-J4 specifically inhibits JMJD3 and UTX, both demethylases of the histone 3 lysine 27 (H3K27) [12]. JMJD3 is highly expressed in neuroblastoma, where its encoding gene *KDM6B* is marked by epigenetic modification characteristic for active transcription [13]. JMJD3 knockdown has been previously shown to decrease cell viability and colony formation in neuroblastoma cell lines, and GSK-J4 is effective in a range of neuroblastoma cell lines by reducing cell viability, inhibiting xenograft growth, and modulating gene expression, including changes in genes associated with neuronal differentiation pathways [13, 14].

In the present study, we have identified and characterized synergistic effects between combined WIP1 inhibition and inhibition of H3K27 demethylases, ultimately aiming for future precision therapies with low toxicity for neuroblastoma patients.

## Material and methods

### Cell culture and reagents

Neuroblastoma cell lines were chosen to represent the genetic heterogeneity of the disease regarding *TP53* mutation status, *MYCN* status, and *PPM1D* expression (Supplementary Fig. S1A). All included cell lines contain a 17q gain, except for CHLA-20, where data on 17q status are missing [15, 16]. *PPM1D* expression was adopted from the Broad Institute’s expression database within the DepMap portal (https://depmap.org/portal) [17]. Cell lines were purchased from ATCC with the exception of CHLA-20 cells, a kind gift from Prof. Christer Einvik (Tromsø, Norway), and nHDF cells (PromoCell, Heidelberg, Germany). All cell lines except CHLA-20 were cultured in RPMI cell culture medium (Gibco, Life Technologies Limited, Paisley, UK) supplemented with 10% heat-inactivated fetal bovine serum (FBS), 2 mM L-glutamine, 100 IU/ml penicillin G, and 100 μg/ml streptomycin (Life Technologies Inc., Stockholm, Sweden) at 37 °C in a humidified 5% CO_2_ atmosphere. CHLA-20 cells were cultured in Iscove’s Modified Dulbecco’s Medium containing 20% Fetal Bovine Serum, 1x Insulin-Transferrin-Selenium (ITS-G), 100 IU/ml penicillin G, and 100 μg/ml streptomycin (all Gibco). The identities of the cell lines (IMR-32 (RRID:CVCL_0346), SK-N-SH (RRID:CVCL_0531), SK-N-AS (RRID:CVCL_1700), SK-N-BE(2) (RRID:CVCL_0528), SK-N-FI (RRID:CVCL_1702), SK-N-DZ (RRID:CVCL_1701) and Kelly (RRID:CVCL_2092)) were verified by short tandem repeat genetic profiling using the PowerPlex 16HS kit (Promega) in 2019. All experiments were performed with mycoplasma-free cells. Experiments were carried out in complete cell culture media with cells from passages below 25. SL-176 was synthesized as previously described [11] and dissolved in DMSO (Sigma-Aldrich). GSK-J4 (Sigma-Aldrich Chemie GmbH, Steinheim, Germany) and Nutlin-3 (Sigma-Aldrich) were also dissolved in DMSO. The DMSO concentration did not exceed 1% v/v in any experiment.

### Drug combination screening

In a semi-automated drug combination screening experiment, the WIP1 inhibitors SL-176 and GSK2830371 were each tested in combination with 527 different clinical and experimental compounds (Supplementary Fig. S1D). For this purpose, a fixed concentration of SL-176 or GSK2830371 (chosen as the IC_25_ of the respective drug in IMR-32 cells) or vehicle was combined with five different concentrations of each of the 527 screening compounds, or vehicle. Combination drugs were dispensed into 384-well plates (Corning, Tewksbury, MA, USA) using an acoustic liquid-handling system (Echo 550, Beckman Coulter, Indianapolis, IN, USA). WIP1 inhibitors, or vehicle, were added at a 5x concentration using a Multidrop Combi Reagent Dispenser (ThermoFisher Scientific, Maltham, MA, USA) in 5 µl of media, and finally 20 μl of single cell suspension (equivalent to 1500-2000 cells) were dispensed into each well. Plates were incubated for 72 hours at standard cell culture conditions, after which cell viability was assessed using an ATP-based luminescent cell viability assay (CellTiterGlo 2.0^™^, Promega, Madison, WI, USA). Luminescence was measured using the EnSight^™^ multimode plate reader in conjunction with the Kaleido^™^ data acquisition and analysis software (PerkinElmer, Waltham, MA, USA). Using the Breeze^™^ software, a drug sensitivity score (DSS) [18], which takes into account both the IC_50_, as well as minimum and maximum inhibition from the fit concentration-response curve, was calculated for each drug, and the ΔDSS was calculated as the difference between DSS of the single screening drug and DSS of the combination with the respective WIP1 inhibitor. ΔDSS ≥ 5 was chosen as cut-off for identifying enhanced combinational effect, likely synergy.

### Small interfering RNA (siRNA) transfections

Three different siRNA constructs targeting *PPM1D* (assay ID: s16127, s16128, s16129, #4392420, Silencer Select, Thermo Fisher) and one scramble (negative control no. 1, #4390843, Silencer Select, Thermo Fisher) siRNA were diluted in Opti-MEM (Gibco) and added, 25 nmol siRNA per well, to 96-well plates together with Lipofectamine RNAiMAX Transfection Reagent (Invitrogen, #13778150). After 5 min incubation, 1.5 × 10^4^ SK-N-BE(2) or SK-N-SH cells were added per well. GSK-J4 (for SK-N-BE(2) 0.1 µM and for SK-N-SH 0.3 µM) or DMSO was added after 24 hours. The plates were scanned in the Incucyte® S3 or SX5 Live-Cell Analysis System (Sartorius) every 24 hours after the addition of drugs for three days, and the cell confluency was measured using the Incucyte software (2023 A and 2024B). Knock-down of *PPM1D* 72 hours after transfection was verified through qPCR.

### Cell viability assays

To determine the effect on cell viability, the formazan-based colorimetric assay WST-1 (Roche, Mannheim, Germany) was used according to the manufacturer’s instructions. Cells were seeded into 96-well plates, left to attach overnight, and drugs were added at the indicated concentrations. After 72 hours, WST-1 was added, and the plates were incubated for 2–3 hours. Absorbance was then measured at 450 nm by a spectrophotometer. The reference wavelength was set to 650 nm. The cell culture medium with WST-1 reagent served as a control for background absorption. All experiments were performed in triplicate, and the mean out of at least three independent experiments is reported.

### Cell confluency assays

For long-term drug exposure experiments, cells were seeded into 48-well plates (2000–4000 cells/well), allowed to attach overnight and then treated with vehicle, GSK-J4, SL-176 or the combination of both in triplicate wells. After 72 hours, the media was replaced for one set of plates. Cell confluence was monitored using the Sartorius Incucyte® S3 or SX5 Live-Cell Live-Cell Analysis System at 0, 72 and 144 hours after drug addition. Images were analyzed using the Incucyte® Basic Analysis Software to assess cell confluence.

### Synergy calculation

To assess the potential synergy of a drug pair, viability assays were performed in the manner described above. Instead of triplicates, concentration matrices were used. The mean of at least three independent experiments generated a dose-response matrix, which was evaluated using the web application SynergyFinder 3.0 (https://synergyfinder.fimm.fi)[19]. Synergy was calculated using the zero interaction potency (ZIP) method [20] and expressed as the synergy score δ, representing the percentual response beyond expectation. Automatic outlier detection and correction were implemented to avoid false-positive findings. δ scores plotted against drug concentrations yielded three-dimensional synergy landscapes, allowing identification of the respective drug concentrations with the strongest synergy, determining the “most synergistic area”.

### Multicellular tumor spheroids (MCTS)

To assess the drug effect in a three-dimensional tumor model, SK-N-AS or IMR-32 neuroblastoma cells were co-cultured with normal human dermal fibroblasts (nHDF). 2500 SK-N-AS or IMR-32 cells were seeded together with 5000 nHDF cells into each well of a low-attachment spheroid 96-well microplate (Corning, Kennebunk, ME, USA or faCellitate, Mannheim, Germany), inducing the formation of multicellular tumor spheroids (MCTS) with nHDF cells at the core and neuroblastoma cells at the periphery [21]. Growth media was supplemented with SYTOX^™^ Green Nucleic Acid Stain (Invitrogen, Carlsbad, CA, USA) as a fluorescent marker for cell membrane disintegration and cell death. Employing the Incucyte® S3 Live-Cell Analysis System (Sartorius), MCTS size, morphology, and fluorescence were assessed at the indicated time points.

### Immunohistochemistry

MCTS were collected, fixed with formaldehyde (3.7–4.0% w/v; PanReac Química, Barcelona, Spain) overnight, and stored in 70% ethanol until paraffin-embedding. Sections were deparaffinized in xylene substitute (Neo-Clear®, Merck KGaA, Darmstadt, Germany) and rehydrated. Heat-induced epitope retrieval was performed in citrate buffer pH 6.0 (Sigma-Aldrich® C9999, KGaA, Darmstadt, Germany) with heating to 110 ˚C for 5 min in a decloaking chamber (Biocare Medical, Concord, CA, USA). Sections were then incubated in blocking buffer (5% normal goat serum (Sigma-Aldrich®) in TBS-T) for 60 min, followed by peroxidase blocking using BLOXALL® (Vector Laboratories, Burlingame, CA, USA) for 10 min at room temperature. Finally, sections were stained with antibodies for cleaved caspase-3 (1:2000; CST 9664) or p21 (1:50; CST 2947, both Cell Signaling Technology, Danvers, MA, USA) overnight at 4 ˚C. After washing and incubation with polymer reagent (ImmPRESS®), DAB substrate (ImmPACT®; both Vector Laboratories) was added for 90 s (cleaved caspase-3) or 5 min (p21). Subsequently, sections were counterstained with Hematoxylin (Abcam, Cambridge, UK), dehydrated, and mounted using SignalStain® Mounting Medium (Cell Signaling Technology).

### Immunoblotting

Cells were seeded in full cell culture medium (RPMI supplemented with 10% fetal bovine serum, penicillin-streptomycin and L-glutamine) at 1–2 × 10^5^ cells/well into six-well plates and left to attach overnight. Drugs at the indicated concentrations, or vehicle, were added and the plates were incubated at standard cell culture conditions for the indicated time. Proteins were then extracted by adding RIPA buffer (Pierce^™^ Ripa, Thermo Scientific, Rockford, IL, USA) supplemented with Halt™ Protease and Phosphatase Inhibitor Cocktail (Thermo Scientific). Samples were vortexed, sonicated, and centrifuged, and the supernatant was collected. Protein concentration was determined using the DC Protein Assay (BioRad, Hercules, CA, USA) according to the manufacturer’s description. Next, 5–15 µg of total protein were separated by reducing SDS-PAGE (NuPAGE®, Invitrogen, Carlsbad, CA, USA) and transferred to a PVDF membrane (Immobilon®-P, Millipore, Sigma-Aldrich). Unspecific binding sites were blocked using 5% non-fat dry milk in TBS-T. Primary and secondary antibody concentrations and incubation conditions are found in Supplementary Table S1. Membranes were incubated with primary antibodies at 4 ˚C overnight, washed with TBS-T (Tris-buffered saline with 0.1% Tween-20; Sigma-Aldrich), and incubated with the secondary antibody for 1 h at room temperature. Following three washing steps, proteins were detected with SuperSignal^™^ West Pico Chemiluminescent Substrate (Thermo Scientific) and visualized using the ImageQuant LAS 4000 imager (GE Healthcare, Chicago, IL, USA). MagicMark XP Western Protein Standard (Thermo Scientific) served as a molecular marker to approximate the mass of detected proteins. Densitometry of the resulting protein bands was performed using the image analysis software ImageJ 1.53k, and readings were normalized to the loading control or, in the case of phosphorylated p38, to total p38 [22]. Complete blots are found in the Supplementary immunoblot material.

### Quantitative PCR (qPCR)

Cells were seeded into 6-well-plates at 1 × 10^5^ (SK-N-BE(2)), 2 × 10^5^ (IMR-32) or 3 × 10^5^ (SK-N-AS) cells per well and left to attach. After 24 hours, cells were treated either with DMSO, SL-176 (final concentration 3 µM for IMR-32 cells; 5 µM for SK-N-BE(2) and SK-N-AS cells), GSK-J4 (final concentration 0.3 µM for IMR-32 cells; 0.5 µM for SK-N-BE(2) and SK-N-AS cells) or a combination of the latter two. In addition, SK-N-AS and IMR-32 cells were treated with SL-176 at the concentration used for the screening (11.2 µM). The treatments lasted for 6, 24, 48 or 72 hours, after which time RNA was extracted using the Qiagen RNeasy kit (Qiagen, Hilden, Germany) according to the manufacturer’s instructions. Briefly, cells were lysed in buffer RLT substituted with beta-mercaptoethanol, homogenized through QiaShredder columns (Qiagen), and then transferred to RNeasy Mini spin columns (Qiagen). A DNase digestion step was included using the RNase-Free DNase Set (Qiagen). After purification, RNA was eluted in RNase-free water, and the RNA concentration was measured using the NanoDrop™ 2000 reader (ThermoFisher). For cDNA synthesis, we used the High-Capacity RNA-to-cDNA™ Kit (Applied Biosystems, ThermoFisher) according to the manufacturer’s instructions. TaqMan probes and primers used for qPCR were *Hs00355782_m1* (*CDKN1A*/p21), *Hs00248075_m1* (*BBC3*/PUMA), *Hs01013292_m1* (*PPM1D*) and *Hs01034249_m1* (*TP53*/p53). *Hs00188166_m1* (SDHA) was included as a housekeeping control and was multiplexed with *Hs00355782_m1*. qPCR was performed in triplicates with 50 ng of cDNA per sample on a QuantStudio Flex 7 system (ThermoFisher) for 40 cycles using standard experimental conditions and including a non-template control. Relative expression was calculated in Microsoft Excel using the delta-delta method, subtracting the mean housekeeping control Ct value from the Ct value of the amplicon assessed and lastly comparing drug-treated samples to vehicle control.

### RNA-sequencing (RNA-seq)

Cells were seeded and treated as described in the section above for 72 hours. RNA was extracted as described in the section above. Total RNA was subjected to quality control with the Agilent TapeStation (Agilent Technologies, Santa Clara, CA, USA) according to the manufacturer’s instructions. To construct libraries suitable for Illumina sequencing, the Illumina TruSeq Stranded mRNA sample preparation protocol, which includes mRNA isolation, cDNA synthesis, ligation of adapters, and amplification of indexed libraries, was used. The yield and quality of the amplified libraries were analyzed using Qubit by ThermoFisher and the Agilent TapeStation. The indexed cDNA libraries were normalized and combined, and the pools were sequenced on the Illumina NextSeq 550 (Illumina, San Diego, CA, USA) for a 75-cycle v2 sequencing run generating 75 bp single-end reads. Basecalling and demultiplexing was performed using CASAVA software (Illumina) with default settings generating Fastq files for further downstream mapping and analysis.

### RNA-seq analysis

Raw data was adapter and quality trimmed using Cutadapt and aligned to the human genome build (GRCh38) using STAR. Gene assignment was performed using FeatureCounts. Differential gene expression was determined using DEseq2. RNA expression profiling data was analyzed by Gene Set Enrichment Analysis (GSEA) [23, 24]. GSEA was performed using GSEA v4.2.3. software (UC San Diego, Broad Institute). The gene sets h.all.v7.5.1.symbols.gmt (Hallmarks) were used to detect the enrichment of different pathways [25]. All gene set files for this analysis were obtained from the GSEA software. To obtain accurate false discovery rates (FDR) and enrichment scores (ES), gene set permutations were set to 10,000 times for this analysis. Venn diagrams were created using the open source software jvenn [26].

### Zebrafish xenografts

SK-N-BE(2) cells expressing green fluorescent protein (GFP) were cultured as described above. Prior to transplantation, cells were detached, incubated 15 min with Hank’s Balanced Salt Solution with Mg^2+^ and Ca^2+^ (Gibco) with additional 5 mM MgCl_2_ and DNAse I (100 µg/ml), and passed over a 40 μm cell strainer to acquire a single-cell suspension. Zebrafish embryos were raised in E3 water containing 30 mg/l phenylthiourea to block pigmentation. The cell suspension was kept on ice before transplantation and then loaded into a microcapillary connected to a Femtojet® 4x (Eppendorf, Hamburg, Germany). A volume of 1.5–2.0 nl with 100–300 cells were injected into the perivitelline space of zebrafish embryos at 48 hours post-fertilization. A 96-well plate was prepared with 250 µl of 1% agarose in 1x E3 medium per well. Anesthetized embryos (160 µg/ml MS-222, Tricaine) were transferred onto the agarose surface. After transplantation, embryos were screened for successful transplantation and treated with either vehicle (DMSO 0.1-0.2%), SL-176 (10 µM), GSK-J4 (1 µM), or a combination of both drugs by adding the substances to the water. The embryos were kept at 33 °C. They were manually oriented into position and imaged at 0 and 72 hours post-exposure using the Acquifer imaging machine (Bruker, Billerica, MA, USA).

An Image J (v. 2.14.0/1.54 f) macro was developed by us to quantify tumor growth. The tumor areas were measured using “analyze particles” with enhanced contrast 0.005. All images were analyzed automatically with the same settings.

### Ethics statement

All procedures complied with the guidelines of the European Union directive 2010/63/EU and with the Swedish legislation on animal experimentation, according to which zebrafish embryos need to be freely feeding to be considered research animals, a state occurring about 5 days post-fertilization. This state was not reached during our experiments.

### Statistical analyses

GraphPad Prism software version 9.3.1 (GraphPad Software, San Diego, California, USA) was used for statistical analyses. The IC_50_ values (inhibitory concentration 50%) were determined from log concentrations-effect curves using non-linear regression analysis.

The sample sizes in the zebrafish embryo study were determined to ensure sufficient statistical power to detect a predefined effect size, based on findings from pilot experiments.

For comparison of three or more groups, one-way ANOVA followed by Dunnett’s multiple-comparisons post-test was used, while nested ANOVA was applied for comparing groups comprised of technical replicates nested within biological replicates. Outliers were identified with Grubbs’ test. *P* < 0.05 was considered significant, and all tests were two-sided.

## Results

### Drug combination screening identifies GSK-J4 as the strongest combination candidate for SL-176

Treatment of IMR-32 and SK-N-AS with SL-176 at IC_25_ (in IMR-32) elicited expected WIP1 downstream effects, as assessed by qPCR (Supplementary Fig. S1C). This drug concentration was selected for the screening.

**Fig. 1:**
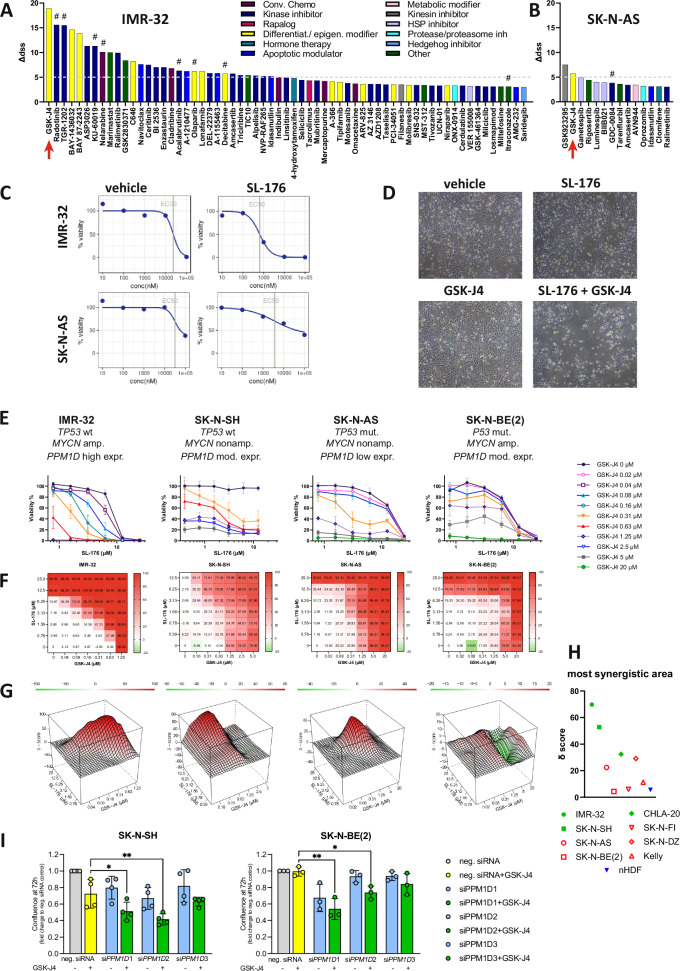
Combined treatment with SL-176 and GSK-J4 induces synergistic cytotoxicity in neuroblastoma cells. Waterfall plots showing the compounds with the highest differential drug sensitivity score (ΔDSS) in combination with SL-176 as compared to vehicle in IMR-32 cells (**A**) and SK-N-AS cells (**B**). Dashed lines indicate ΔDSS = 5, the level considered as cut-off for plausible synergy detected through this assay. Combinations with ΔDSS ≥ 3 are shown. Colors indicate drug classes and # indicates suspected false positives. **C** Dose-response curves for GSK-J4 in IMR-32 and SK-N-AS cells in combination with either vehicle or fixed-dose SL-176 (11 µM). Addition of SL-176 shifted the GSK-J4 dose-response curve one order of magnitude to the left. **D** Morphology of IMR-32 cells treated for 72 hours either with vehicle, SL-176 (3 µM), GSK-J4 (0.3 µM) or the combination. Dose-response curves (**E**), dose-response matrices (**F**) and synergy landscapes (**G**) for four different neuroblastoma cell lines with different genetic characteristics, treated with GSK-J4+/– SL-176. Dose response data shown is the mean of at least three independent experiments from WST-1. Synergy was assessed with the ZIP method using the SynergyFinder tool. **H** δ scores of the areas with the most synergistic dose combinations of SL-176 and GSK-J4 for eight neuroblastoma cell lines and human dermal fibroblasts (nHDF). **I** combining GSK-J4 treatment (0.3 µM for SK-N-SH and 0.1 µM for SK-N-BE(2)) with siRNA-knockdown of PPM1D yielded significantly lower cell confluency after 72 hours for two out of three constructs for each cell line (One-way ANOVA with Dunnett’s post-test, SK-N-SH: neg. siRNA+GSK-J4 vs siPPM1D1 *P* = 0.00496, neg. siRNA+GSK-J4 vs siPPM1D2 *P* = 0.0055 and SK-N-BE(2): neg. siRNA+GSK-J4 vs siPPM1D1 *P* = 0.0016, neg. siRNA+GSK-J4 vs siPPM1D2 *P* = 0.0387). Mean ± SD for four (SK-N-SH) or three (SK-N-BE(2)) independent experiments, performed in triplicates. Knockdown was confirmed to be significant at 60-72% remaining PPM1D expression for SK-N-SH and 11-44% remaining PPM1D expression for SK-N-BE(2).

Drug combination screening in the p53 wild-type cell line, IMR-32, identified 31 compounds with ΔDSS ≥ 5 for the combination with SL-176, suggesting possible synergy (Fig. 1A, Supplementary Table S2). Substances of the drug class “differentiating/epigenetic modifier” were enriched among these hits. The combination compound resulting in the strongest decrease in cell viability, as assessed by this screening, was the H3K27 demethylation inhibitor GSK-J4 with a ΔDSS of 18.9 (Fig. 1A). Review of the corresponding dose-response curves for GSK-J4 revealed a shift to the left by more than one order of magnitude upon addition of SL-176 (Fig. 1C). Analogous screening in the p53-mutated cell line, SK-N-AS, found only two compounds to yield ΔDSS ≥ 5 (Fig. 1B). In this screen, GSK-J4 had the second highest ΔDSS of 5.7, and the EC_50_ was shifted to the left, though incomplete inhibition was observed for both conditions in this cell line, as seen in the dose-response curves (Fig. 1C). A number of drug combinations yielding high ΔDSS in this screening were regarded false-positives after review of the corresponding dose-response curves (Fig. 1A, B, Supplementary Table S2).

In addition, we performed a parallel drug screen with the commercially available WIP1 inhibitor GSK2830371, in which 37 drug combinations yielded ΔDSS ≥ 5 in IMR-32 cells (Supplementary Fig. 2A, Supplementary Table S2). When disregarding putative false-positives, carboplatin had the highest ΔDSS of 11.1. For SK-N-AS cells, only three drugs achieved ΔDSS ≥ 5 when combined with GSK2830371 (Supplementary Fig. S2A). One of these, the mitotic kinesin inhibitor GSK923295, achieved ΔDSS ≥ 5 both in IMR-32 and SK-N-AS cells, but complete inhibition was not achieved in SK-N-AS cells (Supplementary Fig. S2A, Supplementary Table S2).

In IMR-32 cells, both WIP1 inhibitors yielded high ΔDSS in combination with several MDM2 inhibitors, for example idasanutlin (Fig. 1A, Supplementary Fig. S2). The combination of both WIP1 inhibitors, SL-176 and GSK-2830371, with each other also appeared possibly synergistic according to the screening (Fig. 1A). However, the combination of GSK-J4 with GSK2830371 was not among the top hits (Supplementary Fig. S2A).

### Cell viability and proliferation experiments confirmed synergy between GSK-J4 and WIP1 inhibition in neuroblastoma cell lines

The combination of SL-176 and GSK-J4 was selected for further investigation. Morphologically, this combination had an evident effect on cell integrity when comparing to treatment with SL-176 or GSK-J4 alone, or with vehicle (Fig. 1D). Next, we performed cell viability assays in eight different neuroblastoma cell lines, employing 6-8 different concentrations of each drug in a combination matrix. All tested neuroblastoma cell lines, regardless of *TP53* mutation status, *PPM1D* expression or *MYCN* amplification status, displayed additive or synergistic effects. The observed synergy, expressed as δ scores, was highly dependent on the drug concentration ranges chosen (Fig. 1E–G, Supplementary Fig. S3A–C). δ scores for the most synergistic areas ranged from 4.34 to 69.8, and the highest maximal δ score was found for IMR-32 (Fig. 1H). Scrutiny of dose-response matrices detected dose levels at which the combination achieved nearly complete inhibition of cell viability, whereas single-drug treatment induced discernible changes in viability to a much lesser extent.

In comparison, combination effects between GSK2830371 and GSK-J4 were less pronounced, with the most synergistic area δ scores ranging between 5.18 and 17.2 (Supplementary Fig. S2B, C).

Normal human dermal fibroblasts (nHDF) were included as a non-cancerous control and subjected to treatment with SL-176 and GSK-J4 alone, as well as in a matrix with six different concentrations of each drug in combination. Compared to the tumor cells, nHDF cells demonstrated low sensitivity to both single drug and SL-176 and GSK-J4 combination treatment. However, synergistic cytotoxicity occurred at the highest doses (Fig. 1H, Supplementary Fig. S3A–C).

The mechanistic importance of inhibiting WIP1 in conjunction with GSK-J4 treatment was confirmed through knockdown experiments in SK-N-SH and SK-N-BE(2) cells. In both cell lines, two out of three *PPM1D* RNA-binding siRNA constructs in combination with GSK-J4 treatment yielded significantly reduced cell proliferation as compared to GSK-J4 treatment alone (Fig. 1I).

To explore whether alternative targeting of the DNA damage response by inhibiting MDM2 could also be synergistic with *KDM6B* inhibition, we treated five different neuroblastoma cell lines with the combination of GSK-J4 and Nutlin-3. While some synergy was detected, especially in IMR-32 cells, it was notably less pronounced compared to the combination of GSK-J4 and SL-176. Additionally, p53-mutated cell lines were largely resistant to MDM2 inhibition (Supplementary Fig. S3D–F).

### Synergistic effect of SL-176 and GSK-J4 is confirmed in long-term and three-dimensional cell cultures

**Fig. 2:**
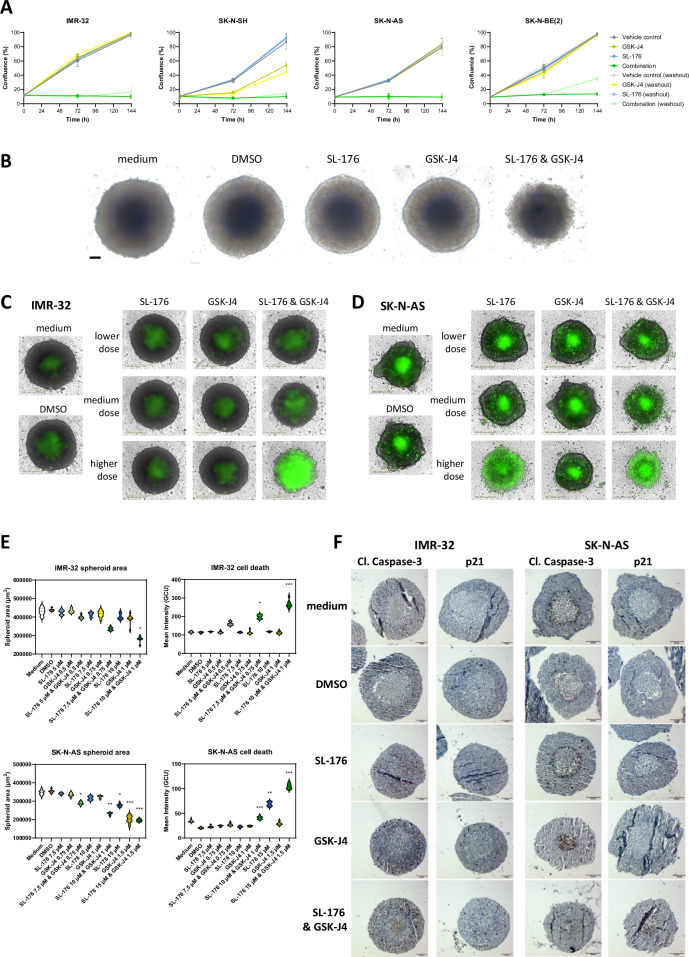
Synergism of SL-176 and GSK-J4 is confirmed in long-term experiments and in neuroblastoma spheroids. **A** confluency of NB cells followed for 144 hours after adding vehicle or drug treatment. Cells were treated with GSK-J4 (0.3 µM in IMR-32, SK-N-AS and SK-N-BE(2) and 0.6 µM in SK-N-SH), SL-176 (3 µM in IMR-32 and 6 µM in SK-N-SH, SK-N-AS and SK-N-BE(2)) or their combination. In the “washout” group, the medium was exchanged at 72 hours. Data represent the mean ± SD from four independent experiments. **B** representative bright-field microscopy images of IMR-32 tumor spheroids exposed to vehicle, SL-176 10 µM, GSK-J4 1 µM, or the combination, for 6 days. Scale bar, 100 µm. Representative bright-field images and overlay with green fluorescence of IMR-32 (**C**) and SK-N-AS (**D**) spheroids after 6 days of treatment. Scale bars, 400 µm. **E** SL-176 and GSK-J4 combination treatment affects both size and viability of IMR-32- and SK-N-AS-derived spheroids in a dose-dependent manner. GCU, green calibrated unit. *p < 0.05; **p < 0.01; ***p < 0.001. Experiments were performed three times (SK-N-AS) or four times (IMR-32), and 3-8 spherocytes were analyzed in each run. **F** immunohistochemistry of IMR-32 and SK-N-AS spheroids detecting cleaved caspase-3 and p21. Scale bars, 100 µm.

Since epigenetic interventions may take longer to manifest, we conducted an extended experiment in which neuroblastoma cell growth was followed for 6 days after the addition of drugs by monitoring cell confluency. In all four cell lines, a strong inhibitory effect on cell growth was observed exclusively for the combined SL-176 and GSK-J4 treatment, and this effect was sustained over the entire observation period. Upon drug removal after 72 hours (“washout”), some regrowth was only observed in SK-N-BE(2) cells (Fig. 2A).

To further explore the synergistic effect of SL-176 and GSK-J4, multi-cellular tumor spheroids consisting of neuroblastoma cells and nHDF cells were employed as a three-dimensional tumor model. In this model, fibroblasts mainly form the center of the sphere while tumor cells position themselves at its periphery, as demonstrated by PHOX2B staining [21, 27].

The combination of SL-176 and GSK-J4 affected both the tumor spheroids’ size and their cell membrane integrity, used as a surrogate marker for viability (Fig. 2B–E). For IMR-32 spheroids, a dose-dependent effect on size and viability was evident for the combination treatment (Fig. 2C, E). No significant change was observed after single-drug treatment in these spheroids. SK-N-AS spheroids showed a dose-dependent significant effect on the size of combination-treated spheroids, whereas single-drug treatment affected spheroid size only at each drug’s highest concentration (SL-176 15 µM and GSK-J4 1.5 µM, respectively) (Fig. 2D, E). Viability was significantly affected when the doses SL-176 10 µM and GSK-J4 1 µM, or higher, were combined (Fig. 2D, E).

Immunohistochemistry of tumor spheroids exposed to the drugs for six days demonstrated an increased number of cells positive for cleaved caspase-3, indicating apoptosis in IMR-32 cells treated with SL-176, GSK-J4 or, especially, the combination. In these spheroids, the number of cells positive for p21 staining, performed as a marker for WIP1 inhibition, was also increased. In SK-N-AS spheroids, there was a surprising degree of cleaved caspase-3-positive fibroblasts at the center. After treatment with SL-176 or the combination, the fraction of tumor cells positive for cleaved caspase-3 was increased. Interestingly, combination treatment elicited positive staining for p21 in the fibroblasts of SK-N-AS spheroids, but not in those of IMR-32 spheroids (Fig. 2F).

### Combined treatment with SL-176 and GSK-J4 induces apoptosis and cell cycle arrest

To elucidate mechanisms underlying the pronounced synergistic effect on neuroblastoma cell viability, as well as the time course of the occurring changes, immunoblots were performed on neuroblastoma cells treated over different timespans with either vehicle, SL-176, GSK-J4 or the combination of SL-176 and GSK-J4.

**Fig. 3:**
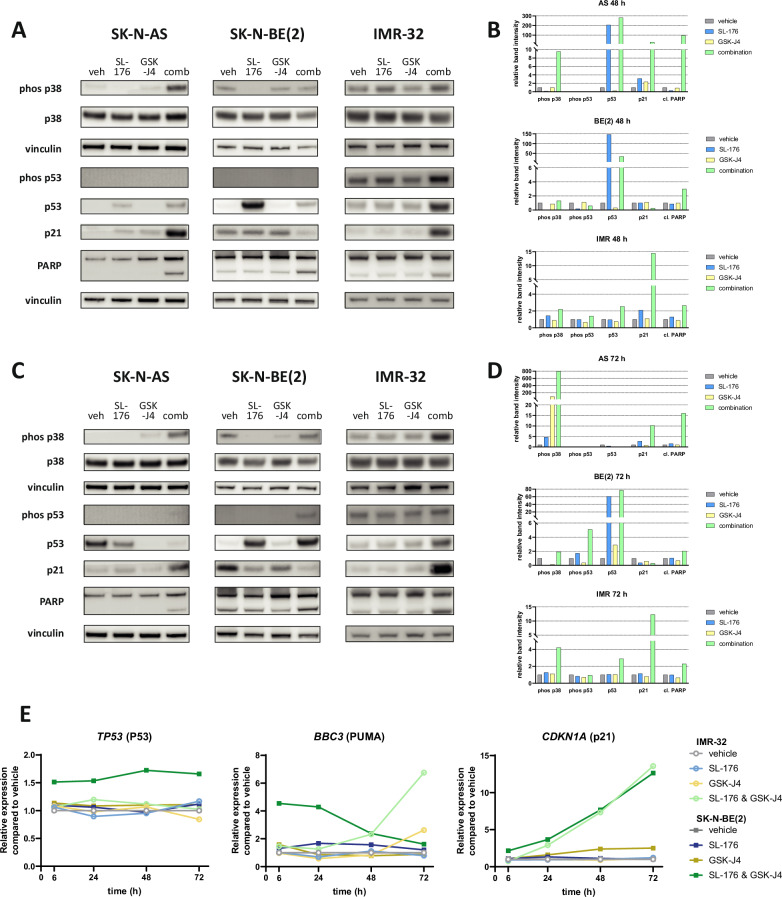
Combined treatment with SL-176 and GSK-J4 induces cell cycle arrest and apoptosis. **A** immunoblots of neuroblastoma cells treated for 48 hours with vehicle, SL-176, GSK-J4 or the combination. **B** corresponding densitometry of bands seen in (A), normalized to vinculin, or, in the case of phosphorylated p38, to total p38. C immunoblots of neuroblastoma cells treated for 72 hours with vehicle, SL-176, GSK-J4 or the combination. **D** corresponding densitometry of bands seen in (**C**), normalized to vinculin, or, in the case of phosphorylated p38, to total p38. Protein expression was investigated with immunoblot and the experiments were repeated with similar results. Please refer to the supplementary immunoblot material for complete immunoblots and Ponceau stainings. **E** relative expression of *TP53*, *BBC3* and *CDKN1A* in IMR-32 and SK-N-BE(2) neuroblastoma cells treated with either vehicle, SL-176 (3–5 µM), GSK-J4 (0.3–0.5 µM) or the combination for 6–72 hours, analyzed with qPCR. The mean of technical triplicates is presented.

Treatment for 1, 6, and 24 hours resulted in few changes in protein expression, but a modest increase in phosphorylated p38 could be seen in IMR-32 cells (Supplementary Fig. S4A). After 48 and 72 hours, however, phosphorylation of WIP1 downstream targets p38, Chk2 and p53 increased, while total p53 and p21 were also upregulated (Fig. 3A–D, Supplementary Fig. S4A). Apoptosis markers poly(ADP-ribose) polymerase (PARP) (Fig. 3A–D) and p53-upregulated modulator of apoptosis (PUMA) (Supplementary Fig. S4B) were affected likewise in the combination-treated cells, while protein levels from cells treated with single drugs did not differ from vehicle-treated cells. Similar findings were made for SK-N-AS and SK-N-BE(2) cells (Fig. 3A–D, Supplementary Fig. S4B). qPCR analysis showed pronounced upregulation of *BBC3* (PUMA) and *CDKN1A* (p21) expression in combination-treated IMR-32, SK-N-BE(2) and SK-N-AS cells, while *TP53* mRNA levels were modestly increased only for SK-N-BE(2) cells treated with the combination (Fig. 3E, Supplementary Fig. S4C).

Interestingly, p21 appeared to be upregulated at transcriptional level in combination-treated IMR-32, SK-N-AS and SK-N-BE(2) cells (Fig. 3E, Supplementary Fig. S4C), but at protein level p21 was lost in the p53-mutated cell line SK-N-BE(2) after 48-72 hours of combined treatment with SL-176 and GSK-J4 (Fig. 3A–D).

In addition, we observed a decrease in N-MYC protein in SK-N-BE(2) cells (Supplementary Fig. S4B).

### RNA-seq confirms pervasive effect of SL-176 and GSK-J4 combination on transcription

**Fig. 4:**
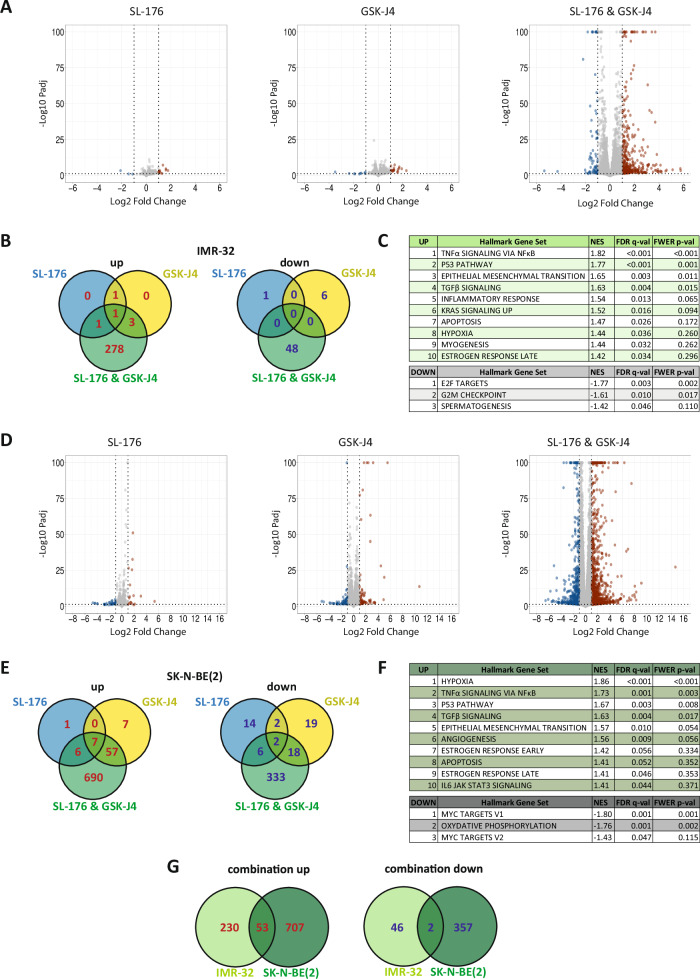
Combination treatment with SL-176 and GSK-J4 induces vast transcriptional changes in neuroblastoma cells. **A** volcano plots showing differentially expressed genes in IMR-32 cells treated for 72 hours with 3 µM SL-176, 0.3 µM GSK-J4, or the combination, as compared to vehicle-treated cells. **B** Venn diagrams showing up- and downregulated genes in IMR-32 cells (Log_2_fold change ≥ 1 or ≤ 1, respectively). **C** list of top enriched or downregulated gene sets for IMR-32. **D** volcano plots showing differentially expressed genes in SK-N-BE(2) cells treated for 72 hours with 5 µM SL-176, 0.5 µM GSK-J4, or the combination, as compared to vehicle-treated cells. **E** Venn diagrams showing up- and downregulated genes in SK-N-BE(2) cells (Log_2_fold change ≥ 1 or ≤ 1, respectively). **F** list of top enriched or downregulated gene sets for SK-N-BE(2). **G** Venn diagrams showing common upregulated (Log_2_fold change ≥ 1) or downregulated genes (Log_2_fold change ≤ 1) between IMR-32 and SK-N-BE(2) cells. In A and D, -Log10 P_adj_ values > 100 were set at 100.

To evaluate combination treatment effects on the transcriptional landscape, we performed RNA-seq on IMR-32 and SK-N-BE(2) cells treated with vehicle, SL-176, GSK-J4, or the combination. As compared to vehicle-treated cells, RNA-seq data showed 331 differentially expressed genes in the combination-treated IMR-32 cells compared to 4 and 11 differentially expressed genes for cells treated with only SL-176 or only GSK-J4, respectively (Fig. 4B). For SK-N-BE(2) cells, these figures were 1119, 38 and 112, respectively (Fig. 4E). These findings were characterized by low p-values, underscoring the dramatic change induced by combined inhibition of WIP1 and JMJD3 (Fig. 4A, D). Although both cell lines demonstrated the same pattern of high numbers of differentially expressed genes specifically in those cells treated with the drug combination, the overlap of those up- or downregulated genes between the two cell lines was small (Fig. 4G). This indicates that transcriptional changes induced by the drug combination cause different transcriptional cascades, depending on different genetic and epigenetic prerequisites and different pathway activations in the cell lines. To further characterize up- and downregulated genes in each cell line, we performed gene set enrichment analysis (GSEA). Among the “Hallmark” gene sets found to be most significantly enriched, the DNA damage response pathways “TNFα signaling via NFκB” and the “p53 pathway” ranked among the top three in both cell lines (Fig. 4C, F). Among the ten gene sets most significantly enriched, seven were shared between the cell lines (Fig. 4C, F). The most significantly downregulated pathways were related to cell cycle in the case of IMR-32 (Fig. 4C), while SK-N-BE(2) cells exhibited downregulation of MYC targets (Fig. 4F), consistent with downregulation of *MYCN* (Supplementary Fig. S5).

### Combination treatment halts tumor growth in zebrafish xenografts

**Fig. 5:**
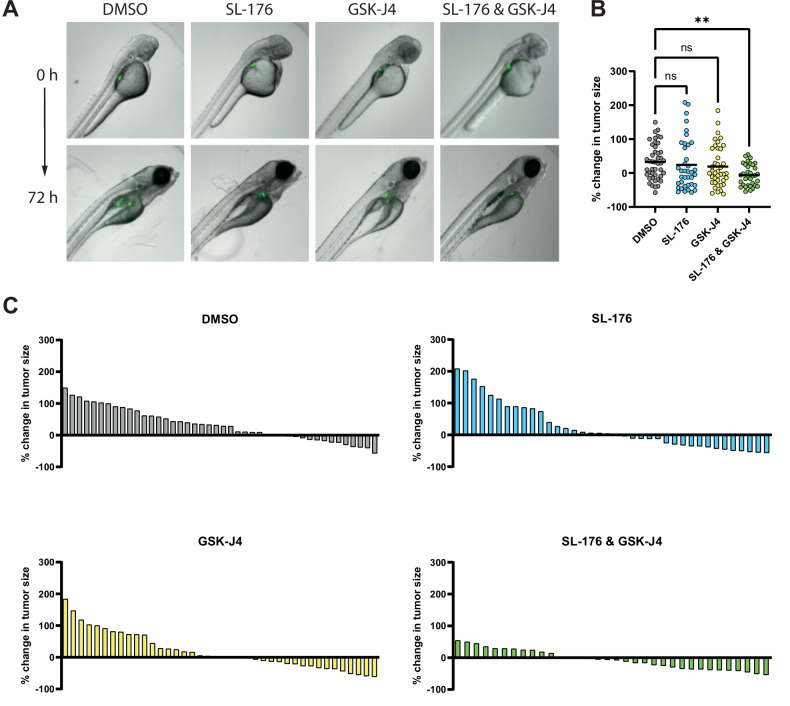
Combination treatment with SL-176 and GSK-J4 reduces the tumor growth of zebrafish neuroblastoma xenografts. **A** Images of zebrafish embryos taken at 0 hours and 72 hours post exposure, representative images were chosen from each group. **B** Scatter plot showing % change in tumor size from base line for the different groups (two experiments pooled together), horizontal line indicating mean. One-way ANOVA with Dunnett’s post-test was performed, comparing each group against the control. One outlier identified by Grubbs’ test was removed. **P = 0.0096. **C** Waterfall plots showing the same data as in B.

In order to confirm the potentiated effect of SL-176 and GSK-J4 in an in vivo setting, a zebrafish xenograft model was used. GFP-transduced SK-N-BE(2) cells were transplanted into the perivitelline space of embryos at 48 hours post-fertilization. Embryos were exposed to either vehicle (DMSO), SL-176 10 µM, GSK-J4 1 µM or a combination of both drugs for 72 h (n = 48 per group, divided in two experiments) (Fig. 5A). No obvious toxicity was detected. The tumor growth in the zebrafish in the combination group was significantly decreased compared to the control group. No significant changes were observed between the groups receiving single treatment when comparing with the vehicle control (Fig. 5B, C).

## Discussion

In this study, we have identified and investigated a drug combination for two highly relevant therapeutic targets, WIP1 and JMJD3, which efficiently induces cell death in several neuroblastoma models. WIP1 is encoded by *PPM1D*, located on chromosome 17q23.2, a chromosome segment frequently gained in neuroblastoma [2, 3]. WIP1 is often overexpressed in neuroblastoma and associated with poor prognosis, and it has been established as an oncogene and therapeutic target in neuroblastoma [9, 28]. With the aim to enhance the effect of WIP1 inhibitors, we performed a drug combination screening where we combined the WIP1 inhibitor SL-176 with 527 different clinical and experimental cancer compounds from different drug classes in two different neuroblastoma cell lines. In an attempt to represent the biological diversity of high-risk neuroblastoma, we selected two cell lines: the *MYCN*-amplified, highly *PPM1D*-expressing cell line IMR-32, and the 11q-deleted, *TP53*-mutated cell line SK-N-AS. The latter cell line is generally considered as resistant to many cancer drugs and also harbors an *NRAS* mutation. While such mutations are rarely found in primary neuroblastomas, alterations in the RAS-MAPK pathway are recurrent features of relapsed neuroblastomas [29–31].

A ranking of differential drug sensitivity scores (ΔDSS) was used to identify promising drug combinations with SL-176. While 31 compounds with ΔDSS ≥ 5 were identified in IMR-32 cells, only two were found in SK-N-AS cells, consistent with this cell line’s mesenchymal and drug-resistant phenotype [32].

Screening in both cell lines yielded GSK-J4 as the most synergistic combination partner, an epigenetically active drug that inhibits H3K27 demethylases JMJD3 and UTX. This was of particular interest since GSK-J4 has previously shown promising preclinical results in the context of neuroblastoma [14]. In our study, we demonstrated synergy between SL-176 and GSK-J4 in experiments assessing cell viability, and observed synergistic effects across a panel of neuroblastoma cell lines, including SK-N-DZ, SK-N-AS, and SK-N-BE(2), which have previously been classified as resistant to GSK-J4 [14]. This pattern was upheld in qPCR and immunoblot experiments, where molecules involved in cell cycle regulation, as well as direct WIP1 targets, were affected by the combination but not by the treatment with single drugs added at equal doses. Our cell line panel included both *TP53* wild-type and mutated cell lines, as well as *MYCN*-amplified and non-amplified cell lines. While our results suggest a tendency toward stronger synergy in *TP53*-wildtype cell lines, efficacy was also evident in mutated cell lines. This is particularly encouraging as *TP53* mutations in NB are often associated with advanced and recurrent disease, highlighting a critical need for novel treatment options. We observed no pattern with regard to synergistic responses between cell lines with and without *MYCN* amplification.

While inhibition of WIP1 is expected to be effective by modulating the activity of its main target, the tumor suppressor p53, as well as other WIP1 targets associated with apoptosis, cell cycle arrest, and DNA repair [33, 34], the mechanism of action of JMJD3 inhibition is less understood. Several efforts have been undertaken to analyze this mechanism. JMJD3 itself has been associated with both tumor suppressor and oncogenic features, even within the field of neuroblastoma [13, 14, 35, 36]. Lochmann et al. found that neuroblastoma cells were particularly sensitive to GSK-J4, leading to differentiation, upregulation of PUMA, and cell death [14]. At the doses studied by us, we confirmed a slight upregulation of PUMA by single-drug GSK-J4 only in IMR-32 cells, while the combination of SL-176 and GSK-J4 elicited a stronger PUMA increase in IMR-32 cells and, importantly, resulted in an upregulation of PUMA mRNA expression also in SK-N-BE(2) cells classified as resistant to GSK-J4 [14].

JMJD3 inhibition has also been shown to cause the accumulation of H3K27me3, disturbing the interaction of transcription factors and resulting in the downregulation of the CDK4/6-pRB-E2F pathway and of *MYCN* in neuroblastoma [13]. Our data partly confirm this model by demonstrating that the combination treatment inhibiting JMJD3 and WIP1 significantly reduces *MYCN* mRNA expression and protein expression in SK-N-BE(2) cells (Supplementary Figs. S4 and S5). However, at the doses studied, we did not observe any effects on *MYCN* expression by single-drug GSK-J4 treatment in SK-N-BE(2). IMR-32 cells, on the other hand, showed an increase in *MYCN* mRNA, but a decrease in protein expression as measured by immunoblot (Supplementary Figs. S4B and S5), and E2F targets were the most significantly downregulated gene set in our gene set enrichment analysis in IMR-32 cells. Furthermore, D’Oto et al. observed an upregulation of p21 by GSK-J4 treatment and showed that the promoter of its encoding gene *CDKN1A* was marked by enhanced chromatin accessibility after GSK-J4 treatment [13]. We likewise observed an increase of *CDKN1A* mRNA in response to single-drug GSK-J4 treatment in SK-N-BE(2) cells, while combined treatment with GSK-J4 and SL-176 induced a much stronger and more significant upregulation of p21 (Fig. 3, Supplementary Fig. S5). This effect might be due to an increased chromatin accessibility caused by GSK-J4 and also to the increased level of active p53 caused by SL-176-mediated WIP1 inhibition.

In agreement with D’Oto et al., another study analyzing commonly downregulated genes in different neuroblastoma cell lines treated with GSK-J4, also highlighted the role of the E2F signaling pathway [37]. Here, the gene *SAPCD2* was identified as a key player, operating via cytoplasmatic redistribution of E2F7. The resulting decreased nuclear E2F7 concentrations would decrease E2F activator inhibition and confer increased transcription of E2F target genes [37]. In our RNA-seq dataset we found significant downregulation of *SAPCD2* upon SL-176 and GSK-J4 combination treatment in SK-N-BE(2), but not in IMR-32 cells, in which this gene also was expressed at a lower level (Supplementary Fig. S5).

For both neuroblastoma cell lines subjected to RNA-seq, gene set enrichment analysis (GSEA) showed enrichment of both the NF-κB and the p53 pathways (Fig. 4). This is in line with our expectations as we seek, through inhibition of WIP1, to indirectly promote p53 effects. The NF-κB pathway has been shown to be transcriptionally targeted by JMJD3 to promote progression of melanoma [38]. NF-κB is often viewed as an antagonist of p53; however, it has recently been observed that these two pathways are closely interlinked with each other and WIP1, and the direction of their interaction has been demonstrated to be context-dependent [39, 40]. GSEA also demonstrated induction of the TGFβ signaling pathway, implicated in neurogenesis and neuronal commitment of stem cells in both IMR-32 and SK-N-AS cells. Interestingly, it has been shown that JMJD3 is critically involved in neuronal differentiation and may cooperate with p53 in this function [36, 41–43]. WIP1, on the other hand, has been proposed as a negative regulator of the TGFβ pathway by dephosphorylating its central mediator SMAD4 [44].

The analysis of RNA-seq data revealed a large number of differentially expressed genes, many of which are involved in canonical pathways of DNA damage response and cell cycle control. As the incubation time for the treatment with SL-176 and/or GSK-J4 was 72 hours, some cell death did occur at this time point. This makes it difficult to discern primary (drug-induced) vs. secondary (cell-death-induced) changes in these genes’ expression. Furthermore, dosing in single- versus combination-treated cells was equimolar rather than titrated to equal effect on viability. This allows quantitative observations of synergistic effects, while qualitative changes achievable by single drugs at higher concentrations cannot be assessed. However, it is interesting that SK-N-BE(2) cells show such a vast number of differentially expressed genes in the RNA-seq data upon combination treatment, despite the fact that the synergy examination based on cell viability was not as strongly positive as in IMR-32. This speaks in favor of the changes in gene expression as a primary effect of the drug combination rather than a secondary effect caused by cell death-induced DNA damage response. To address this question in depth, further RNA-seq experiments spanning several time points and dosing levels would be solicited.

As a potential limitation of our synergism-finding approach, the drug combination screening in each cell line was performed as a single experiment, but with five different concentrations of each combination drug, from which dose-response curves and resulting DSS were calculated. Hence, outliers may have had an impact on the resulting dose-response curve. This can lead to high DSS, which must be viewed as “false positives”, and which are usually identified by inspection of the corresponding dose-response curves. In the waterfall plots displayed, putative “false positives” are indicated. Conversely, however, outliers could also lead to a synergistic combination not being identified by this assay, and this has not been corrected for in our approach. Regardless of this potential methodological challenge, H3K27 demethylase inhibitor GSK-J4 stood out as the most promising candidate for combination with WIP1 inhibitor SL-176, and the synergy was confirmed using multiple assays.

Both the drug combination screening and the immunoblot readout reveal a different profile of SL-176 as compared to the commercially available WIP1 inhibitor GSK2830371 [7]. Differences in effect between SL-176 and GSK2830371 are in line with previously observed differential drug sensitivities depending on cell lines’ *TP53* mutational status [9]. Further studies are needed to elucidate the mechanistic differences between these two WIP1 inhibitors. The fact that both WIP1 inhibitors together seem to combine synergistically, according to the drug combination screening (Fig. 1), may suggest that each achieves partial inhibition of WIP1 and that both complement each other in inhibiting this target. We further verified the enhanced effect of combining WIP inhibition with GSK-J4 treatment through siRNA-mediated knockdown of *PPM1D*. However, further experiments may be required to confidently rule out potential off-target effects that may impact our results. Novel insights into the tertiary structure of WIP1 could help advance the understanding of this protein’s interactions and aid in the design of improved inhibitors [45].

As expected from their mechanisms of action, combination of either WIP1 inhibitor with MDM2 inhibitors resulted in high ΔDSS in the screening in IMR-32 cells (Fig. 1A, Supplementary Fig. S2). For GSK2830371, this is in good concordance with the literature [8, 46–49]. Since MDM2 inhibitors, like WIP1 inhibitors, indirectly reactivate p53, they might also be synergistic with H3K27 demethylase inhibition. To explore this, we tested the combination of Nutlin-3 with GSK-J4, detecting some, albeit weaker, synergism (Supplementary Fig. S3).

The potentiated effect of SL-176 and GSK-J4 was upheld in vivo, as tested in a zebrafish xenograft experiment. Probably owing to the technically challenging micromanipulation, this model has an inherent large variability, which is also reflected in the range of relative tumor growth in our control group [50, 51]. However, the larvae treated with the drug combination display clearly reduced tumor volumes, thus confirming in vitro experiments.

Taken together, inhibition of WIP1 and JMJD3 appears to be synergistic in neuroblastoma cell lines. In all assays and regarding all readouts, spanning from viability analyses in monolayer and 3D cell culture models to qPCR, immunoblot, and RNA-seq, the combined effect of SL-176 and GSK-J4 is clearly over-additive. In many instances, doses of SL-176 and GSK-J4 that did not convey any change when used as single drugs, elicit marked alterations when combined with each other.

While these are very encouraging results, there are, to our knowledge, currently no WIP1 or JMJD3 inhibitors in clinical development. The compounds used in this study all suffer from insufficient pharmacokinetic properties, precluding their clinical application. However, new WIP1-inhibiting lead substances based on alternative small molecule scaffolds have been introduced, offering hope for the future development of novel, clinically viable WIP1 inhibitors [52].

## Conclusion

We have identified strong synergy for the drug combination of WIP1 inhibitor SL-176 and H3K27 demethylase inhibitor GSK-J4 in neuroblastoma cell lines and zebrafish xenografts. These two drugs combine to induce cell cycle arrest and apoptosis. Although the precise mechanism of this synergism remains to be determined, our data support that WIP1 and JMJD3 are important targets in neuroblastoma and merit further investigation aiming at clinical implementation, especially in combination with each other.

## Supplementary information

Supplementary Figure S1

Supplementary Figure S2

Supplementary Figure S3

Supplementary Figure S4

Supplementary Figure S5

Supplementary Table S1

Supplementary Table S2

Supplementary Immunoblot Material

## Data Availability

RNA-Seq data associated with this study has been deposited to the Swedish National Data Service (SND) under the accession number #2025-72/1, 10.48723/rkz9-n137. Drug combination screening data are included as Supplementary Table S2. Other raw data are available from the corresponding author upon reasonable request.
