# Supplementary material for: Targeted inhibition of WIP1 and histone H3K27 demethylase activity synergistically suppresses neuroblastoma growth
Source: Cell Death Dis. 2025 Apr 19;16(1):318. doi: 10.1038/s41419-025-07658-1 (PMC12009370; doi:10.1038/s41419-025-07658-1)
Supplement: Supplementary file 1 — Supplementary Figure S1 [file 41419_2025_7658_MOESM1_ESM.pdf]

Supplementary Figure S1

A **Genetic characteristics of neuroblastoma cell lines**

	IMR-32	SK-N-SH	SK-N-AS	SK-N-BE(2)	CHLA-20	SK-N-FI	SK-N-DZ	Kelly
TP53	wt	wt	mut	mut	wt	mut	mut	mut
MYCN	amp	nonamp	nonamp	amp	nonamp	nonamp	amp	amp
***PPM1D* expr.**	high	moderate	low	moderate	*[data missing]*	low	moderate	moderate
**17q**	gain	gain	gain	gain	*[data missing]*	gain	gain	gain

B ***PPM1D* expression**

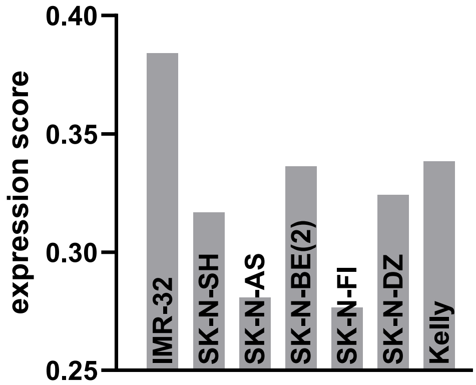

C **Effect of single-drug SL-176 at screening concentration**

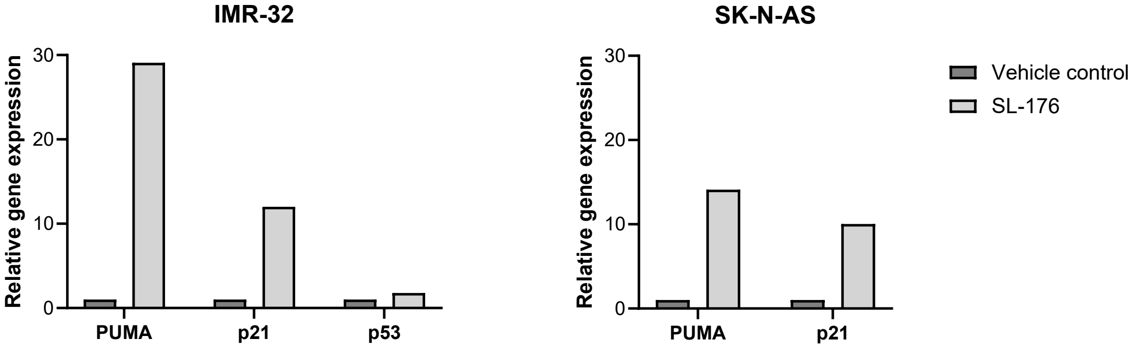

D **Drug classes in screening**

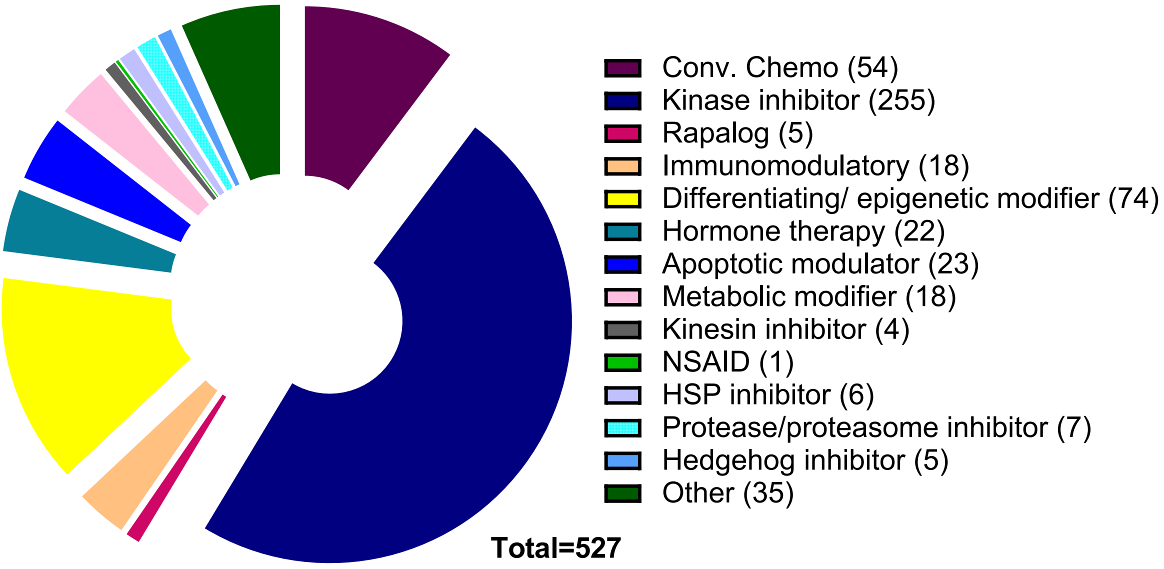

Supplementary Figure S1. Cell lines and drug combination screening: A, table showing genetic characteristics of NB cell lines included in the study. B, *PPM1D* expression enrichment scores from the DepMap portal for NB cell lines included in the study. C, relative gene expression of *TP53* (p53) and its downstream effectors *BBC3* (PUMA) and *CDKN1A* (p21) after 24 hours treatment with either vehicle or 11.2 µM SL-176, the concentration used for the drug screening. qPCR analysis; mean of technical triplicates is presented. D, drug classes included in drug combination screening.
