# Supplementary material for: Targeted inhibition of WIP1 and histone H3K27 demethylase activity synergistically suppresses neuroblastoma growth
Source: Cell Death Dis. 2025 Apr 19;16(1):318. doi: 10.1038/s41419-025-07658-1 (PMC12009370; doi:10.1038/s41419-025-07658-1)
Supplement: Supplementary file 2 — Supplementary Figure S2 [file 41419_2025_7658_MOESM2_ESM.pdf]

Supplementary Figure S2

A

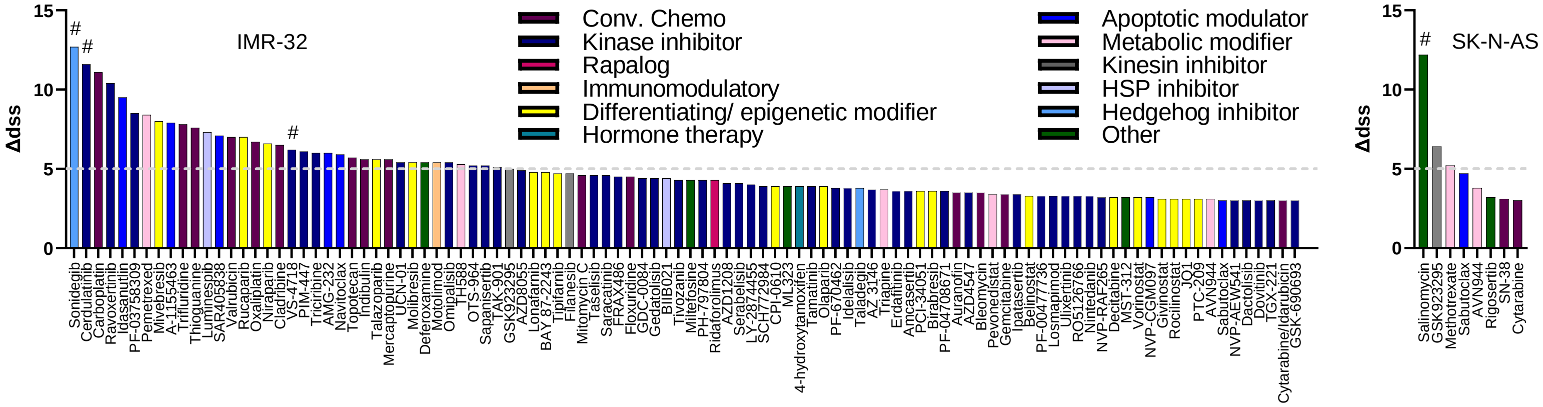

B

Cell Line	Drug combination	Synergy score	Most synergistic area score	Method
IMR-32	GSK-J4 -GSK2830371	4.98	17.20	ZIP
SK-N-AS	GSK-J4 - GSK2830371	-2.43	5.86	ZIP
SK-N-BE(2)	GSK-J4 - GSK2830371	1.87	5.18	ZIP

C

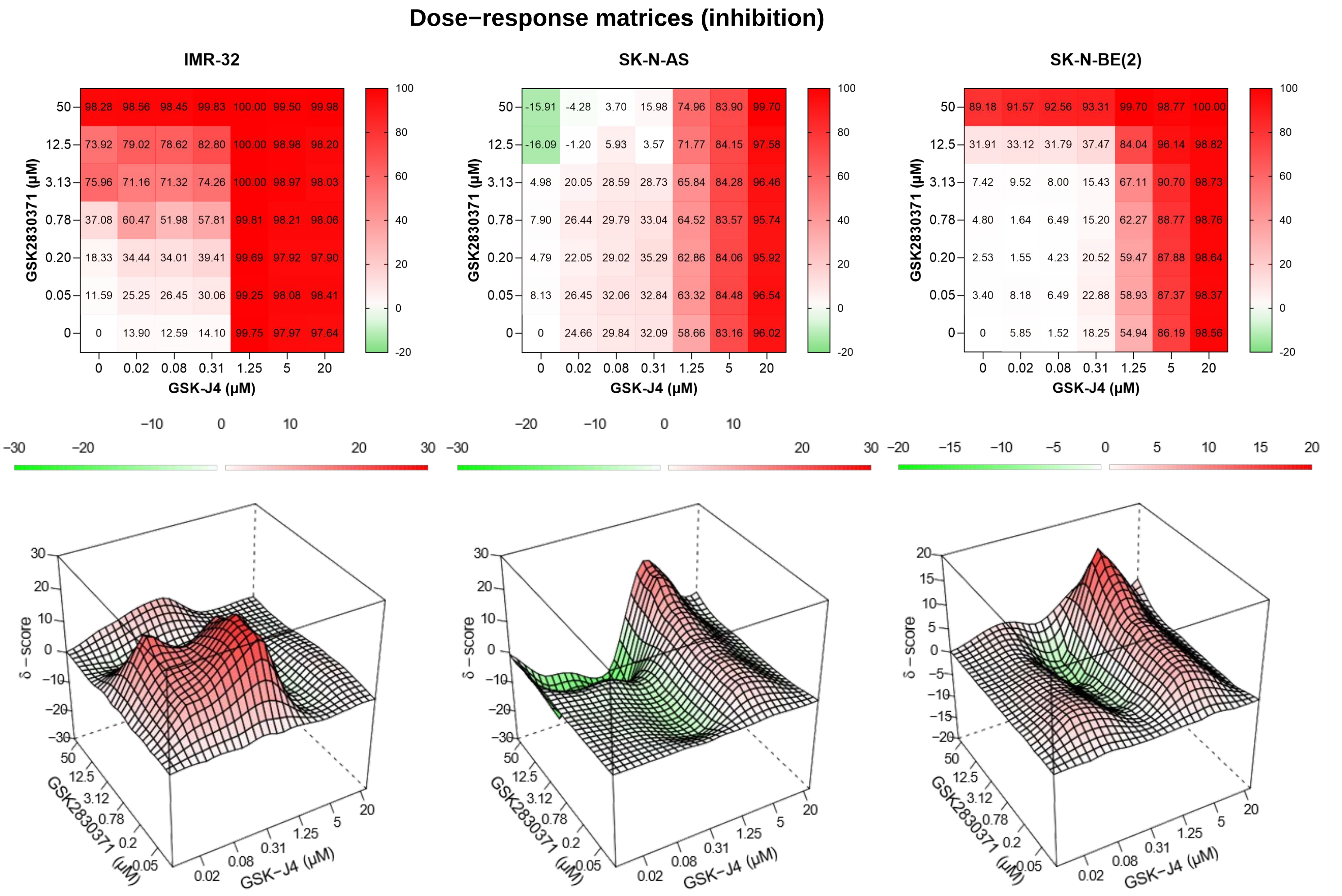

Supplementary Figure S2: WIP1 inhibitor GSK2830371. A, waterfall plots showing the compounds with the highest differential drug sensitivity score (ΔDSS) in combination with GSK2830371 as compared to vehicle in IMR-32 cells (left) and SK-N-AS cells (right). Dashed lines mark ΔDSS = 5. Combinations with ΔDSS ≥3 are shown. Colors indicate drug classes and # indicates suspected false positives. B, Synergy scores for the combination of GSK2830371 with GSK-J4. C, corresponding dose-response matrices (upper panel) and synergism landscapes (lower panel) for three different neuroblastoma cell lines treated with GSK2830371 +/- GSK-J4. Dose response data shown is the mean of at least two independent experiments. Synergy was assessed with the ZIP method using the SynergyFinder tool.
