# Supplementary material for: Targeted inhibition of WIP1 and histone H3K27 demethylase activity synergistically suppresses neuroblastoma growth
Source: Cell Death Dis. 2025 Apr 19;16(1):318. doi: 10.1038/s41419-025-07658-1 (PMC12009370; doi:10.1038/s41419-025-07658-1)
Supplement: Supplementary file 3 — Supplementary Figure S3 [file 41419_2025_7658_MOESM3_ESM.pdf]

Supplementary Figure S3

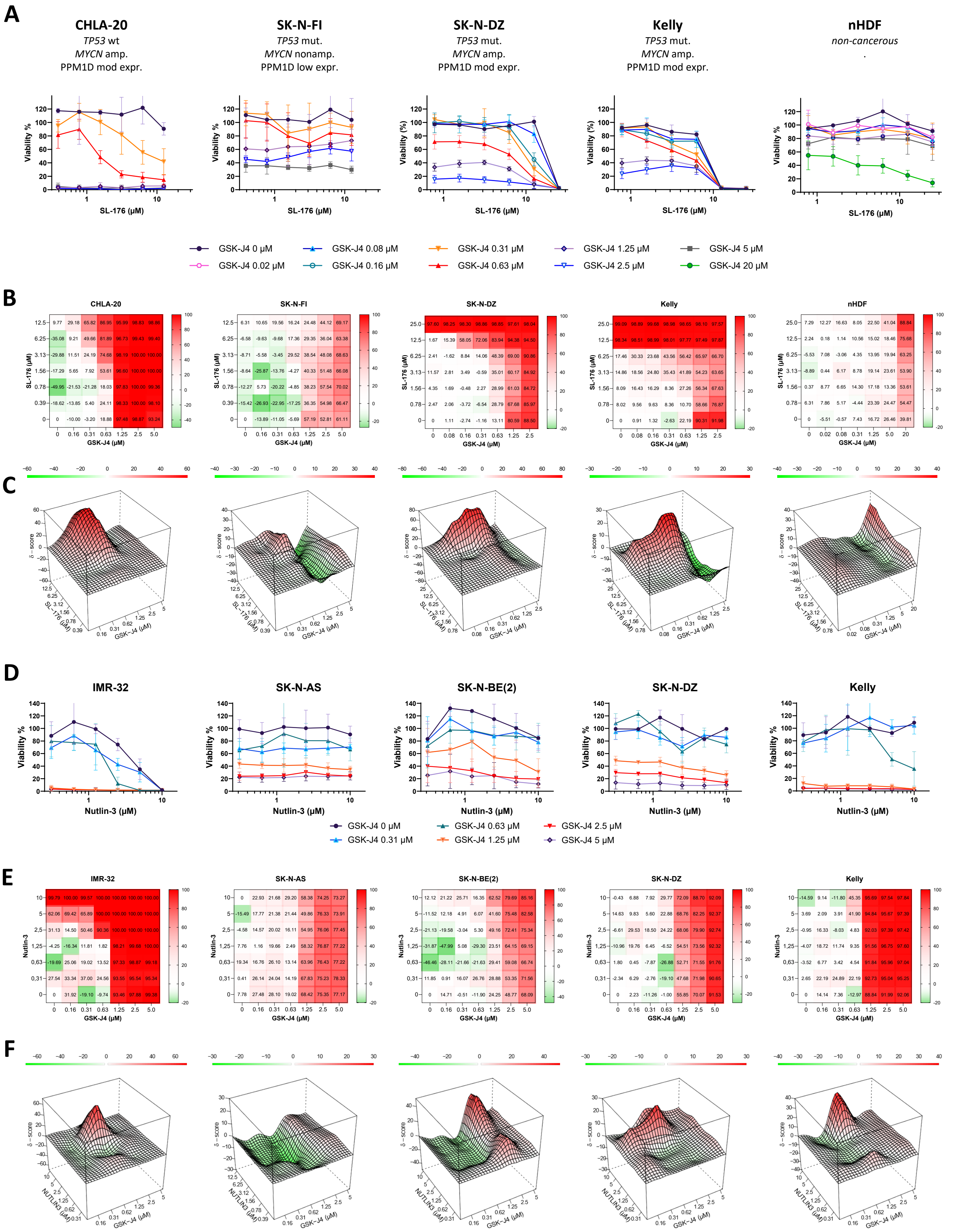

Supplementary Figure S3: A-C, Combination treatment of additional NB cell lines and normal human dermal fibroblasts (nHDF) with SL-176 and GSK-J4. Dose-response curves (A), dose-response matrices (B) and synergy landscapes (C). D-E, combination of GSK-J4 with the MDM2 inhibitor Nutlin-3. Dose response data shown is the mean of at least three independent experiments from WST-1. Synergy was assessed with the ZIP method using the SynergyFinder tool.
