# Supplementary material for: Targeted inhibition of WIP1 and histone H3K27 demethylase activity synergistically suppresses neuroblastoma growth
Source: Cell Death Dis. 2025 Apr 19;16(1):318. doi: 10.1038/s41419-025-07658-1 (PMC12009370; doi:10.1038/s41419-025-07658-1)
Supplement: Supplementary file 4 — Supplementary Figure S4 [file 41419_2025_7658_MOESM4_ESM.pdf]

Supplementary Figure S4

A **6 timepoints assessed in IMR-32 cells**

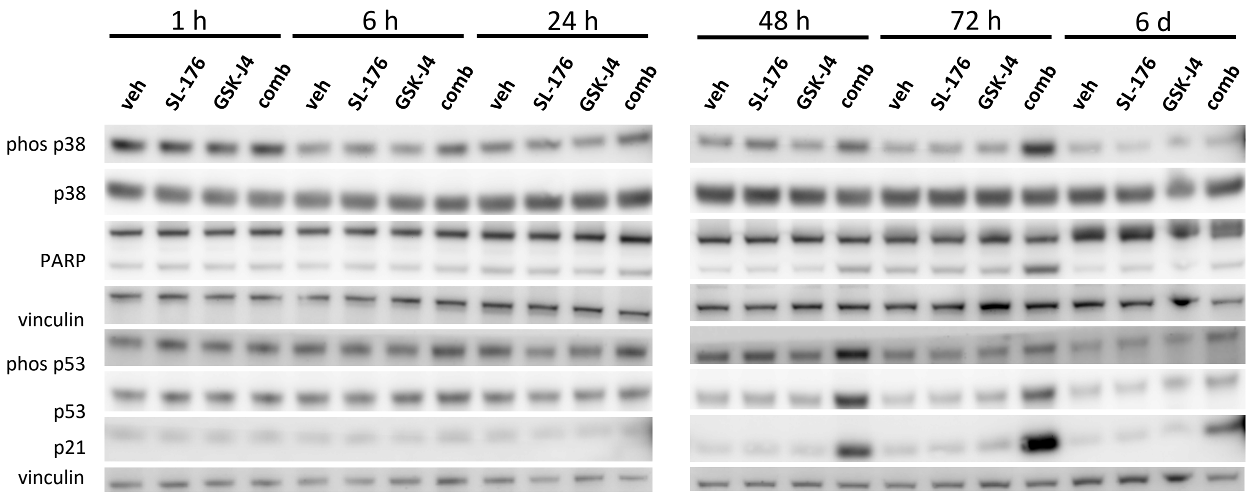

B **Additional proteins assessed in three cell lines at two timepoints**

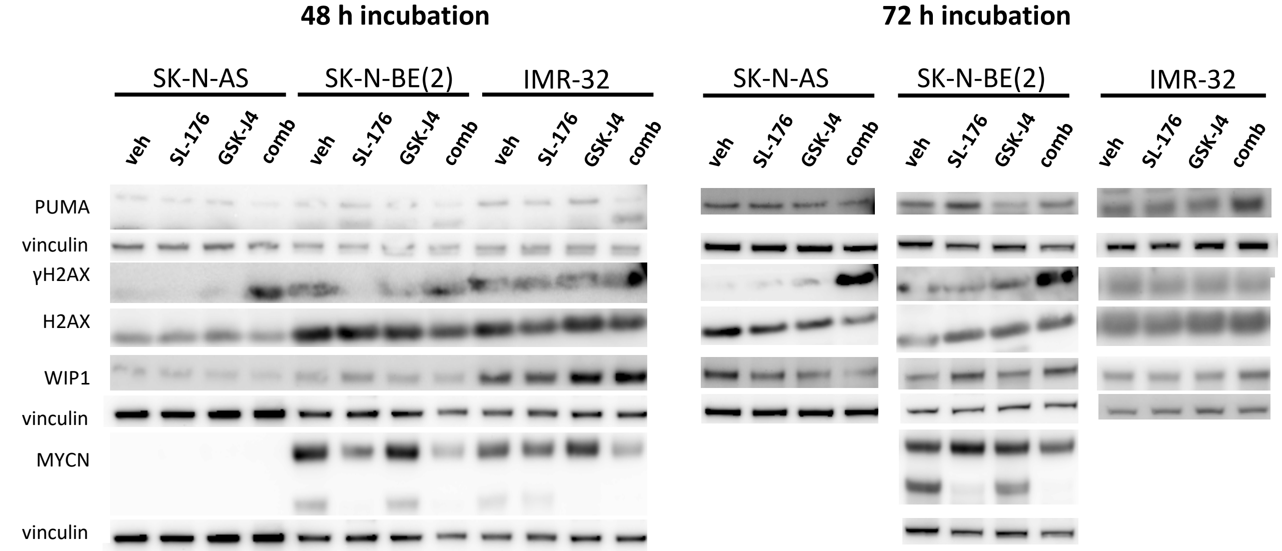

C **qPCR in SK-N-AS cells**

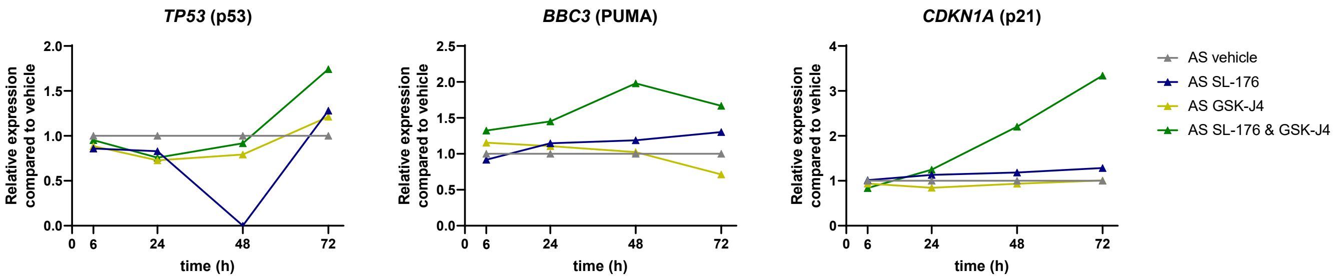

Supplementary Figure S4: Additional immunoblot and qPCR results. Please refer to supplementary immunoblot material for complete immunoblots and Ponceau stainings. A, IMR-32 cells treated with vehicle, SL-176, GSK-J4 or the combination, assessed at different time points. Note that blots for 48 h and 72 h are also shown in Figure 3AC. B, additional proteins assessed by immunoblot. C, relative expression of *TP53*, *BBC3* and *CDKN1A* in SK-N-AS cells treated with either vehicle, SL-176, GSK-J4 or the combination for 6-72 h, analyzed with qPCR. The mean of technical triplicates is presented.
