# Supplementary material for: Targeted inhibition of WIP1 and histone H3K27 demethylase activity synergistically suppresses neuroblastoma growth
Source: Cell Death Dis. 2025 Apr 19;16(1):318. doi: 10.1038/s41419-025-07658-1 (PMC12009370; doi:10.1038/s41419-025-07658-1)
Supplement: Supplementary file 5 — Supplementary Figure S5 [file 41419_2025_7658_MOESM5_ESM.pdf]

Supplementary Figure S5

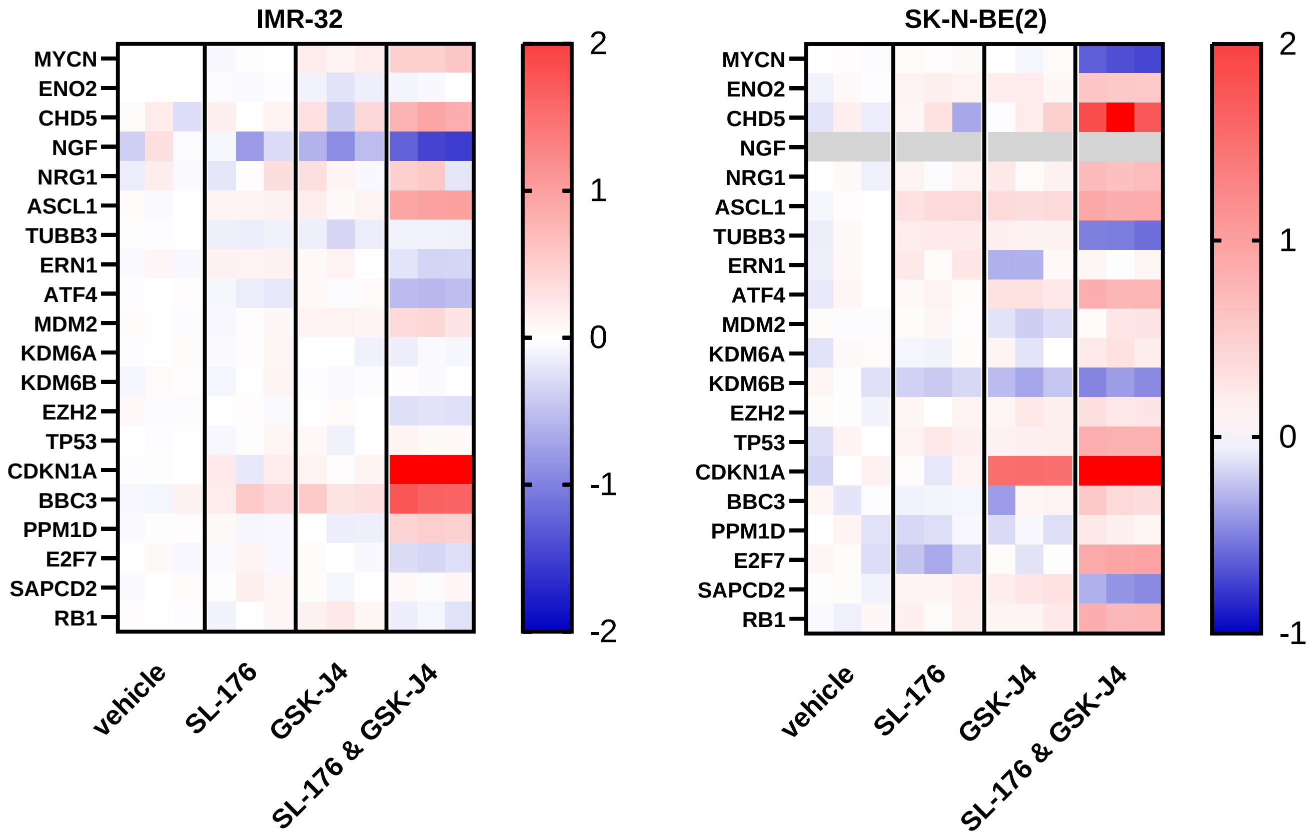

Supplementary Figure S5: Relative expression of selected genes analyzed by RNA-seq, Log2 fold change.
