# Supplementary material for: Targeted inhibition of WIP1 and histone H3K27 demethylase activity synergistically suppresses neuroblastoma growth
Source: Cell Death Dis. 2025 Apr 19;16(1):318. doi: 10.1038/s41419-025-07658-1 (PMC12009370; doi:10.1038/s41419-025-07658-1)

Supplementary material:

Immunoblot additional data and raw data

Disposition

- Fig. 3A: cell lines SK-N-AS, SK-N-BE(2), IMR-32 at 48 hours
- Fig. 3C: cell lines SK-N-AS, SK-N-BE(2), IMR-32 at 72 hours
- Blots and Ponceau stainings corresponding to Fig. 3A-D
- Blots corresponding to supplementary figure S4

*time specifications denote exposure times during image acquisition
*Please note that some membranes were cut after blotting, and each part incubated with different antibodies
*Please refer to Suppl. Table S1 for reagents and conditions

48 hours – Fig. 3A

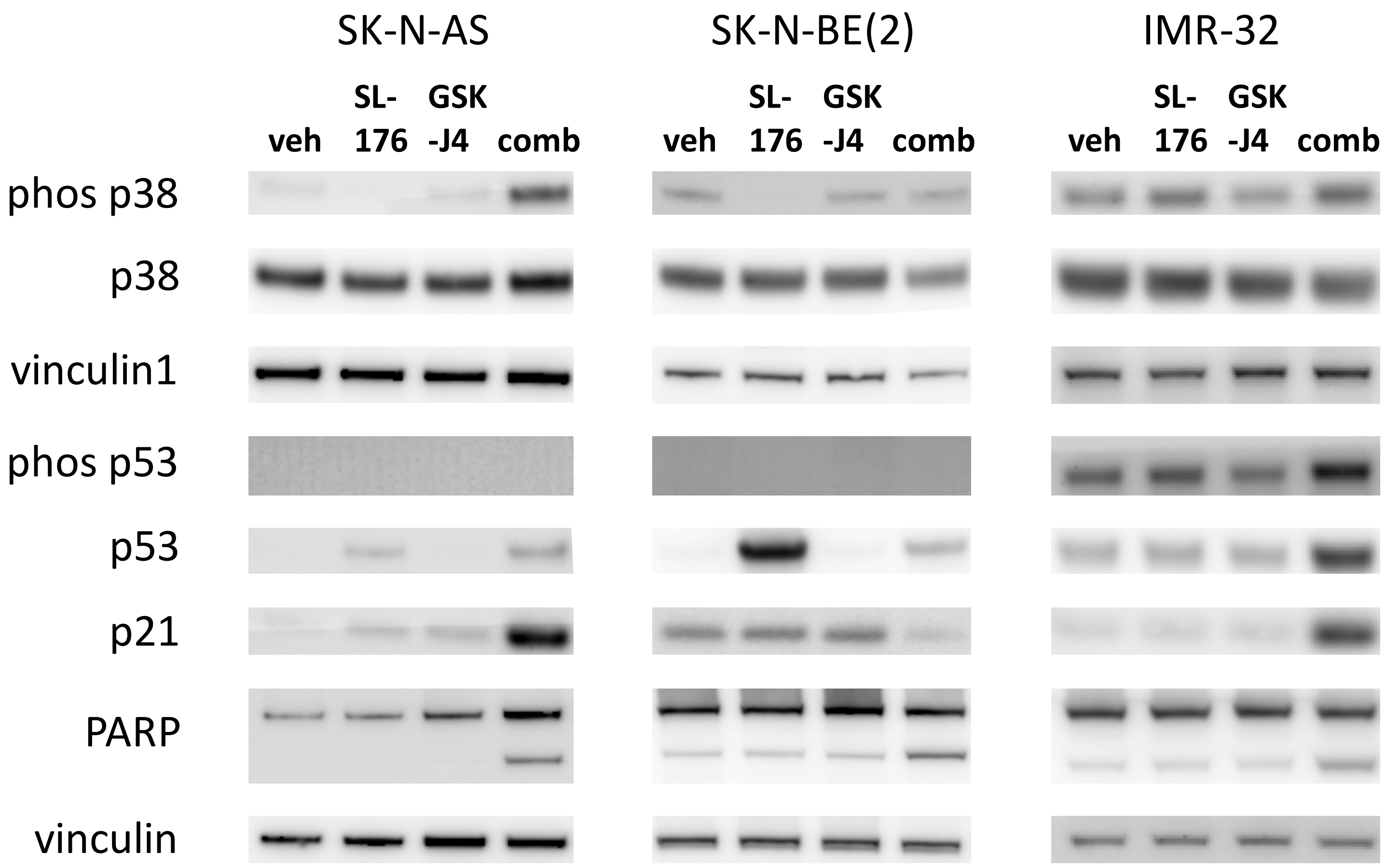

72 hours – Fig. 3C

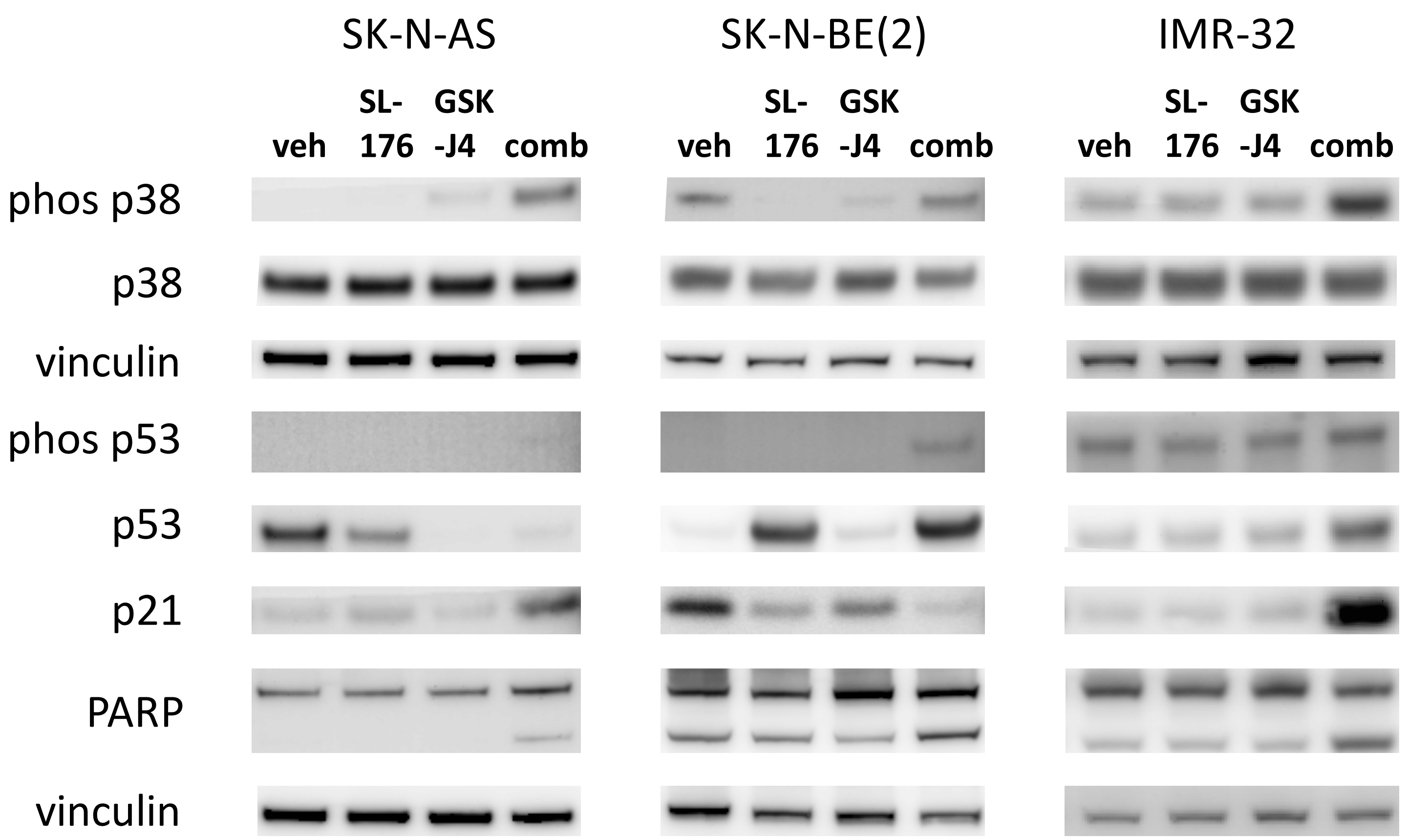

Complete blots corresponding to figure 3A,C

2020-DT-10:IMR-32 PARP, phos p38, p38, vinculin;48-72h

Loading:

Laddr	Laddr	48h	48h	48h	48h	72h	72h	72h	72 h	6 d	6 d	6 d	6 d	Laddr
Magic Mark	Visual	Veh	SL-176	GSK-J4	Comb	Veh	SL-176	GSK-J4	Comb	Veh	SL-176	GSK-J4	comb	Magic Mark

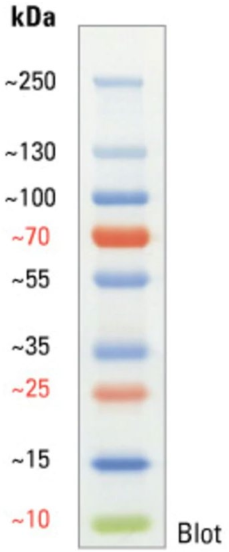

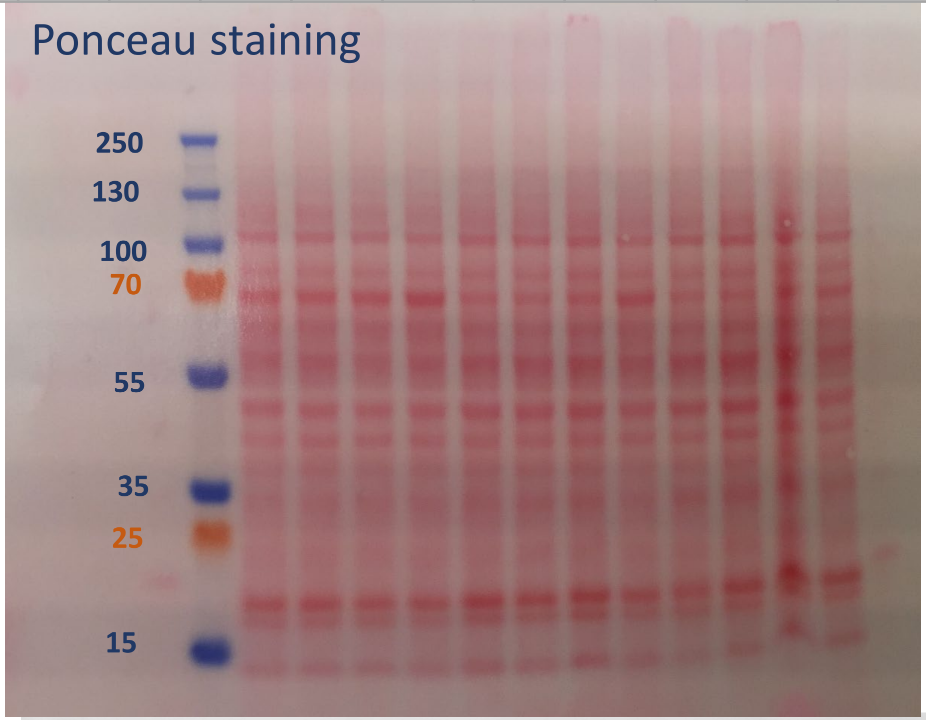

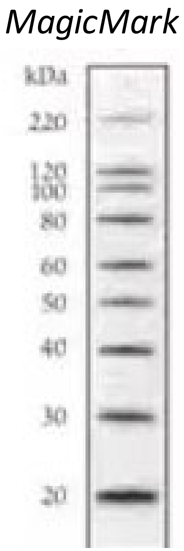

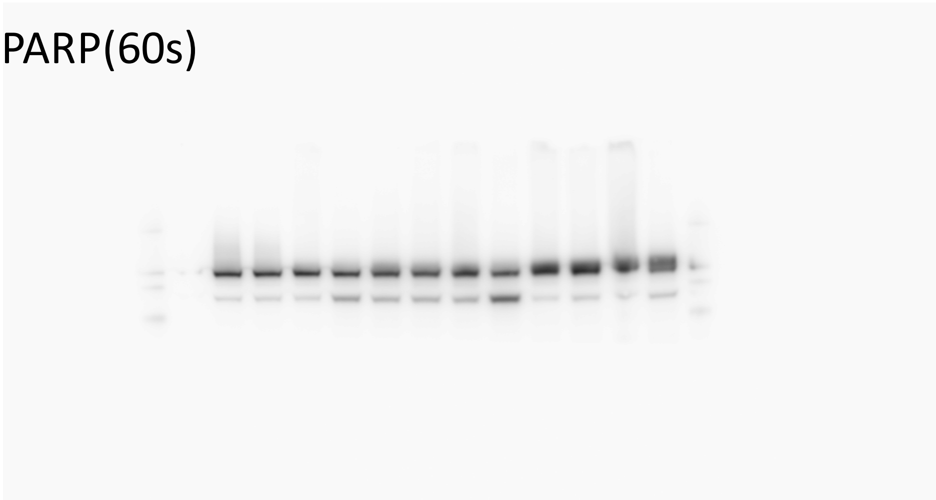

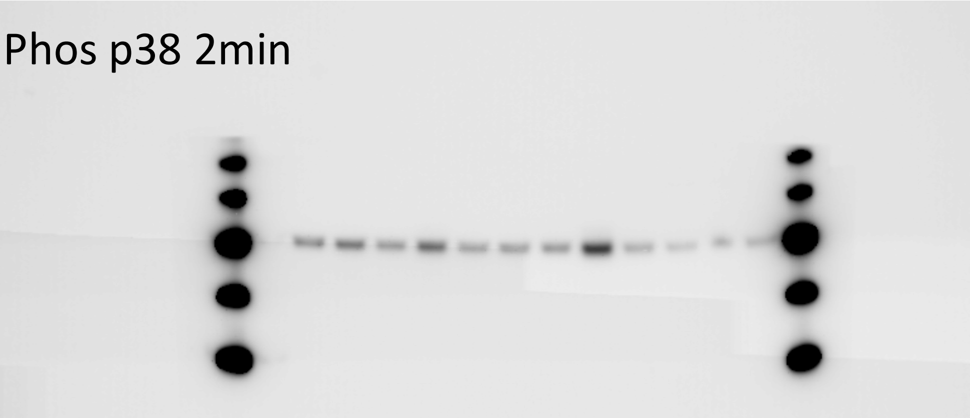

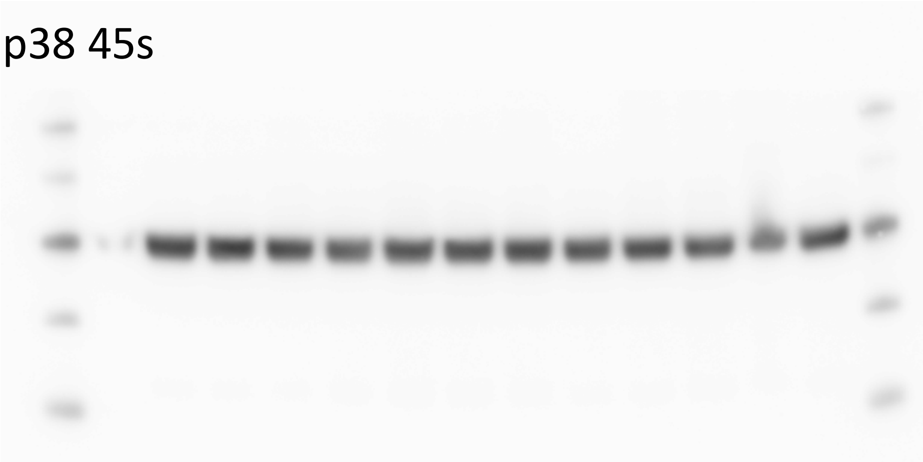

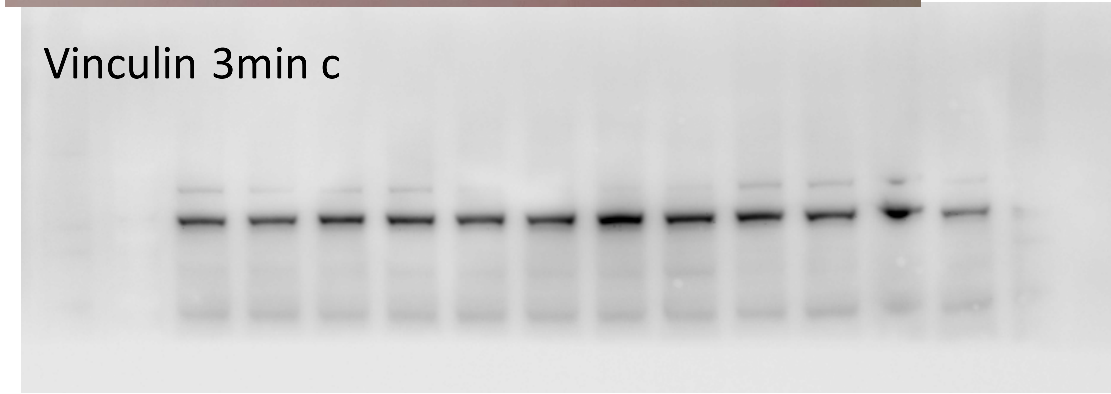

2020-DT-12:IMR-32 phos p53, p53, p21, vinculin;48-72h

Loading:

Laddr	Laddr	48h	48h	48h	48h	72h	72h	72h	72 h	6 d	6 d	6 d	6 d	Laddr
Magic Mark	Visual	Veh	SL-176	GSK-J4	Comb	Veh	SL-176	GSK-J4	Comb	Veh	SL-176	GSK-J4	comb	Magic Mark

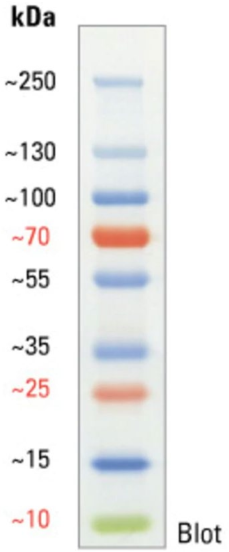

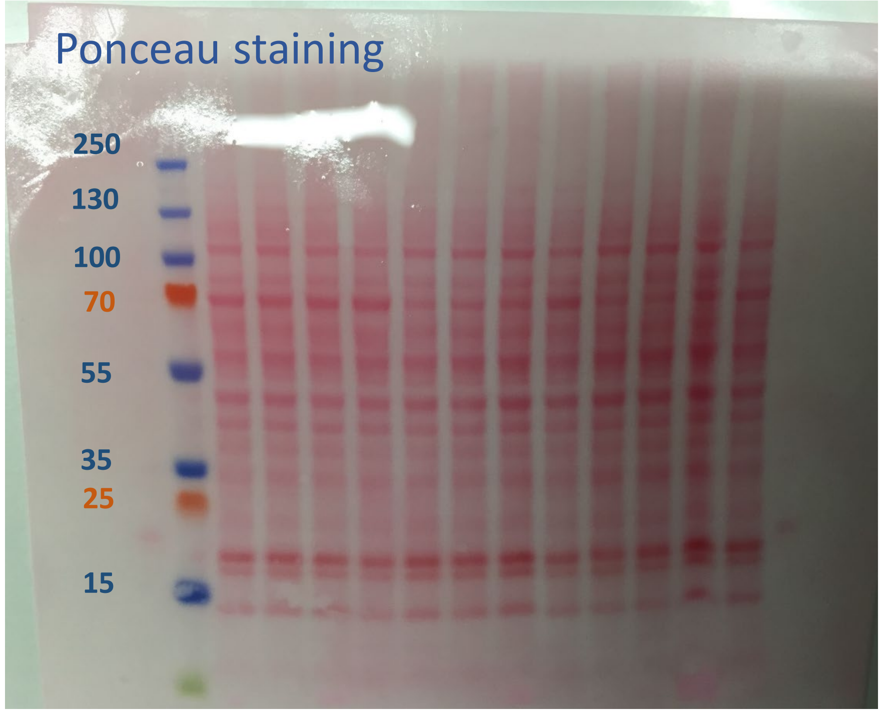

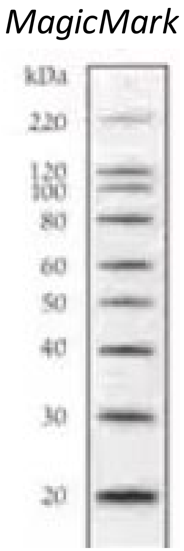

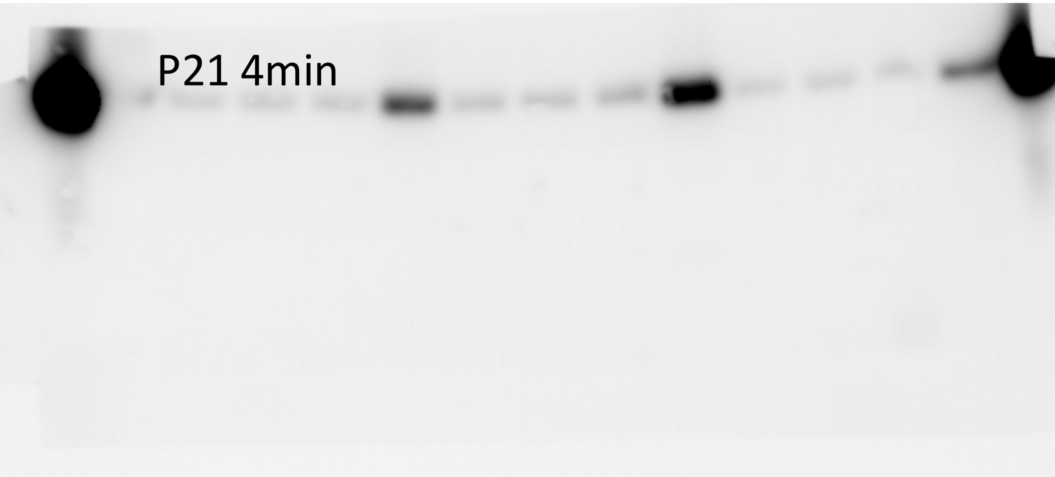

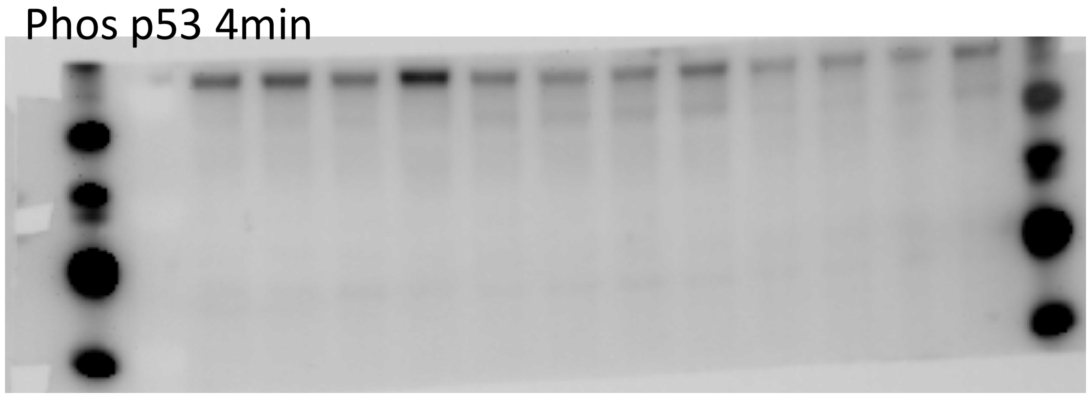

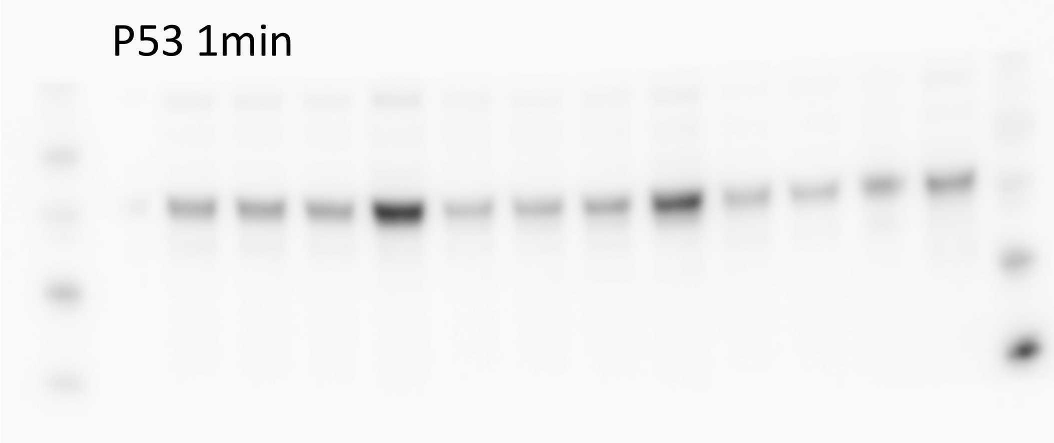

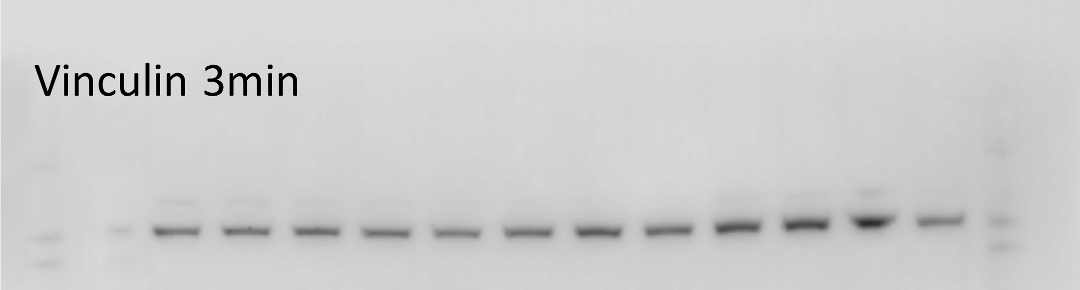

2021-DT-05:BE2 phos p53, p53, p21, PARP vinculin;48-72h

Loading:

Laddr	Laddr	48h	48h	48h	48h	72h	72h	72h	72 h	Laddr	Laddr
Magic Mark	Visual	Veh	SL-176	GSK-J4	Comb	Veh	SL-176	GSK-J4	Comb	Magic Mark	Visual

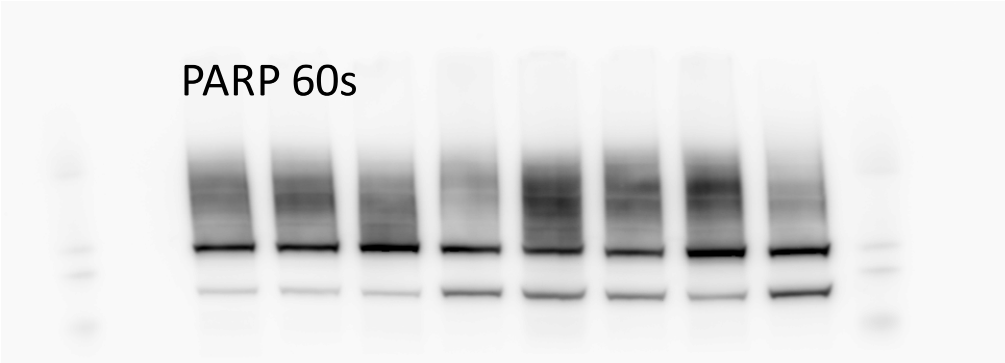

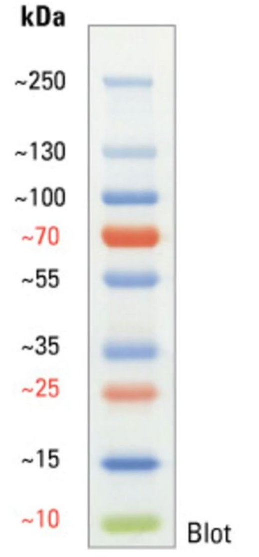

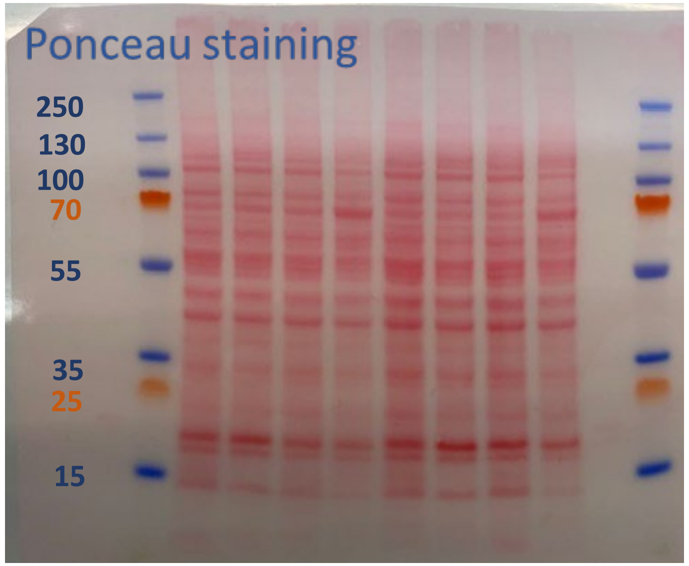

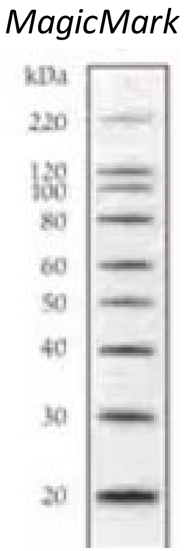

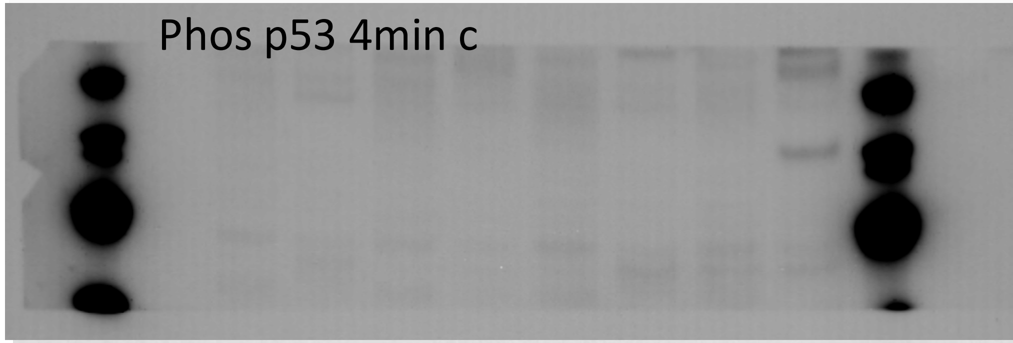

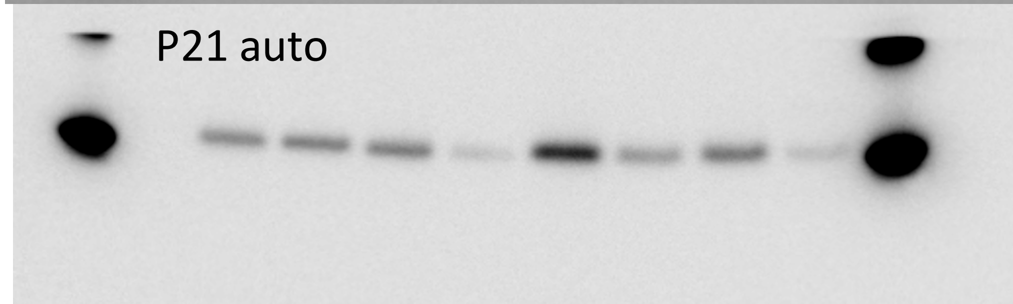

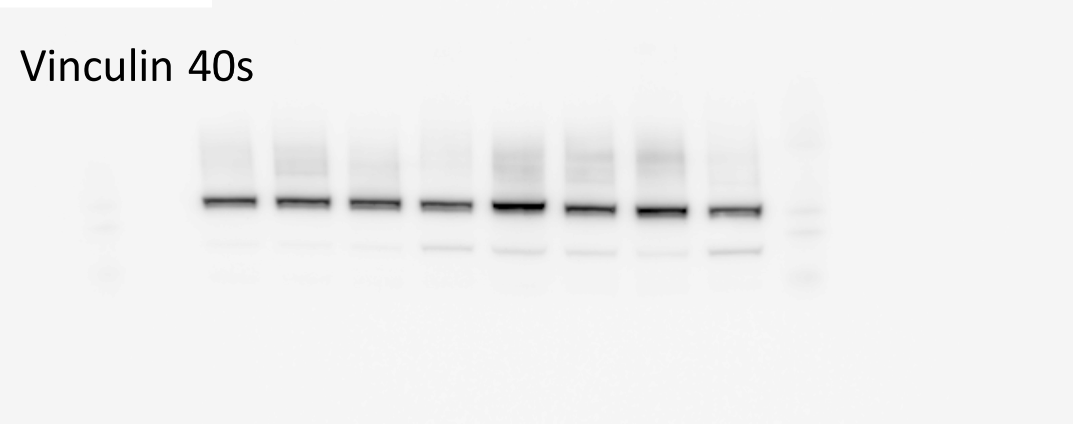

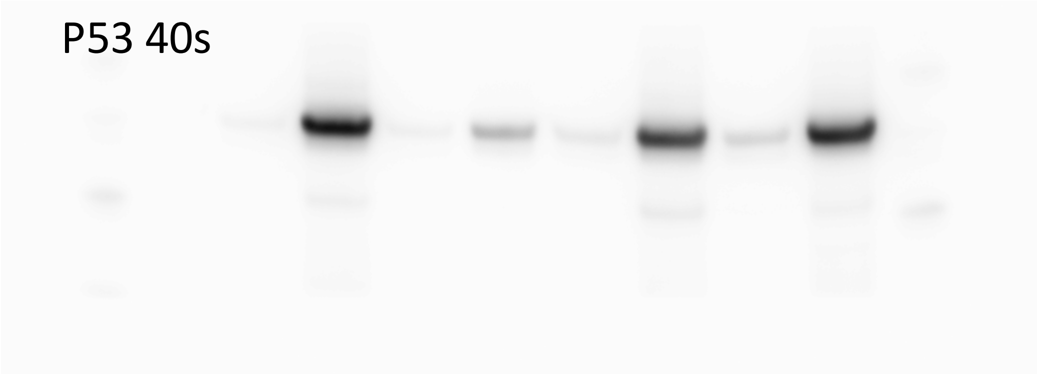

2021-DT-06:
BE2 phos p38, p38, vinculin;48-72h

Loading:

Laddr	Laddr	48h	48h	48h	48h	72h	72h	72h	72 h	Laddr	Laddr
Magic Mark	Visual	Veh	SL-176	GSK-J4	Comb	Veh	SL-176	GSK-J4	Comb	Magic Mark	Visual

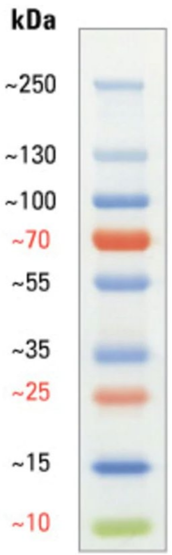

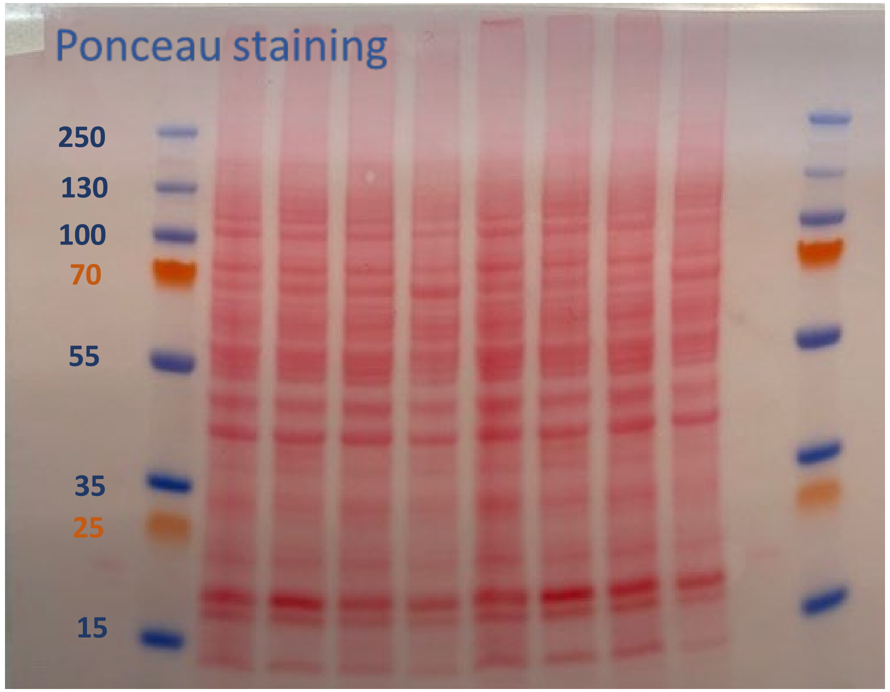

MagicMark

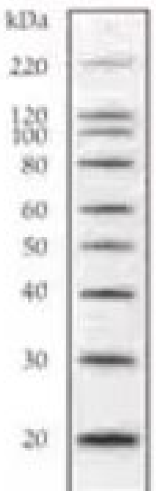

Phos p38 2min c

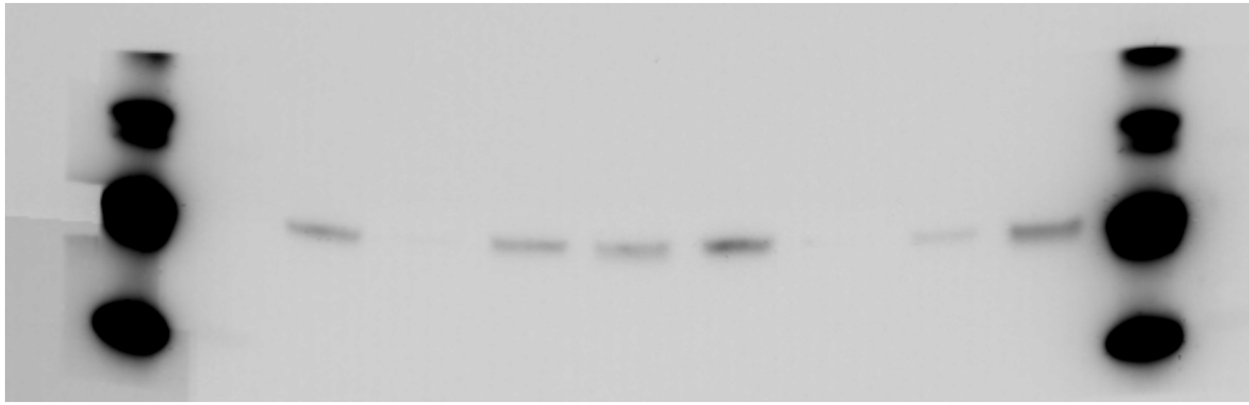

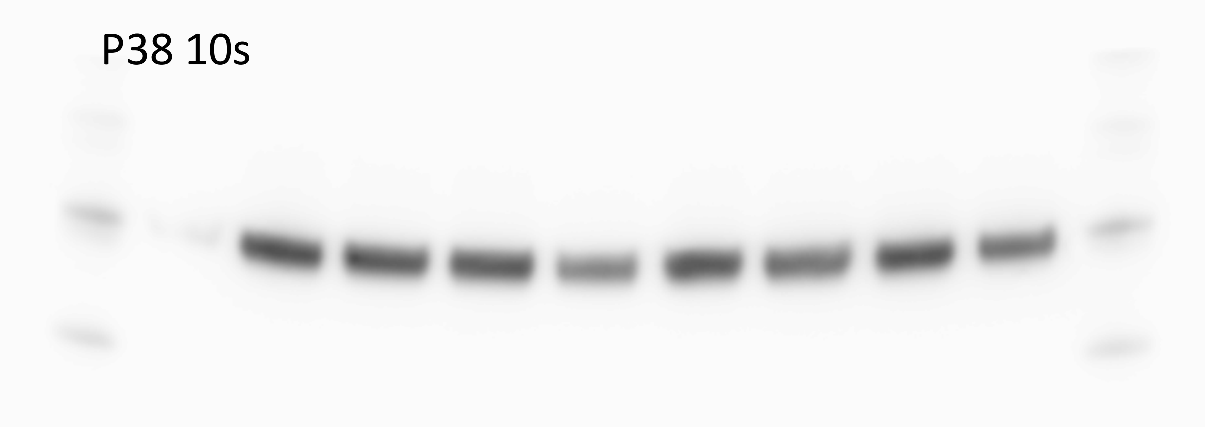

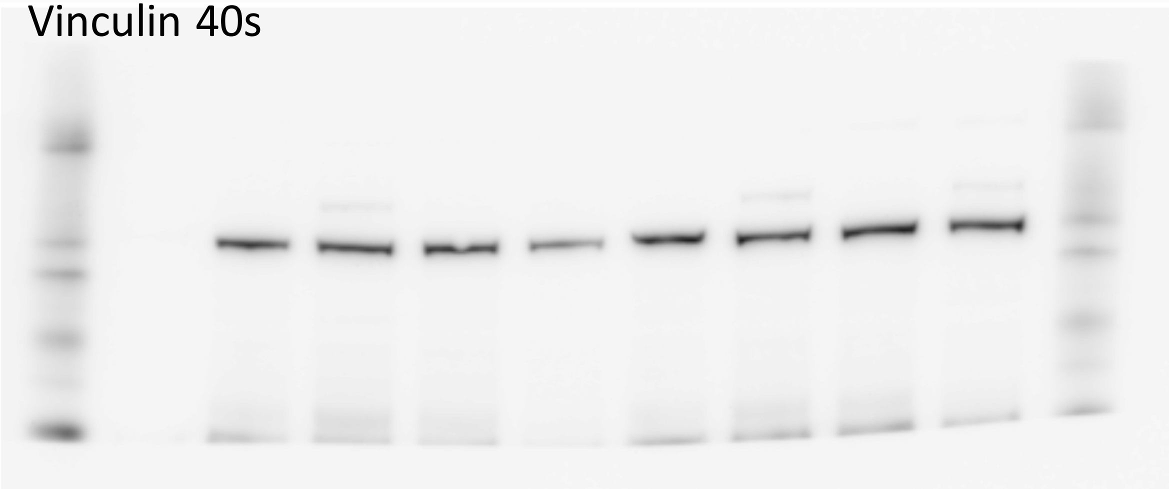

2021-DT-07:AS phos p53, p53, p21, PARP vinculin;48-72h

Loading:

Laddr	Laddr	48h	48h	48h	48h	72h	72h	72h	72 h	Laddr	Laddr
Magic Mark	Visual	Veh	SL-176	GSK-J4	Comb	Veh	SL-176	GSK-J4	Comb	Magic Mark	Visual

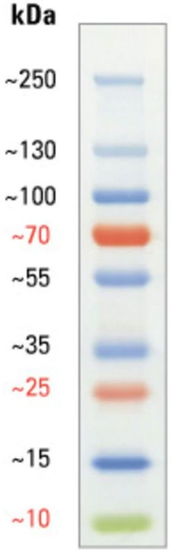

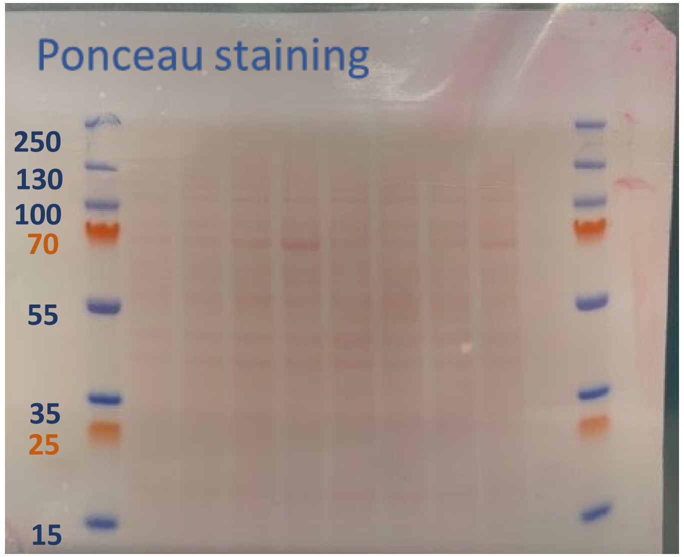

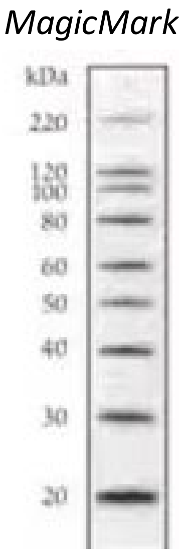

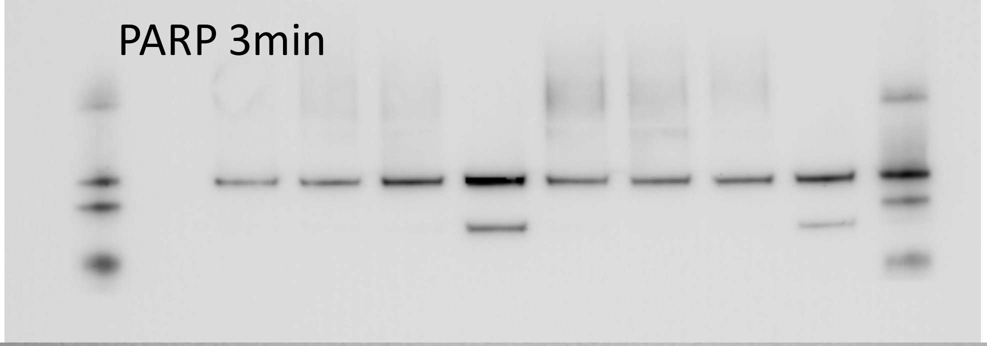

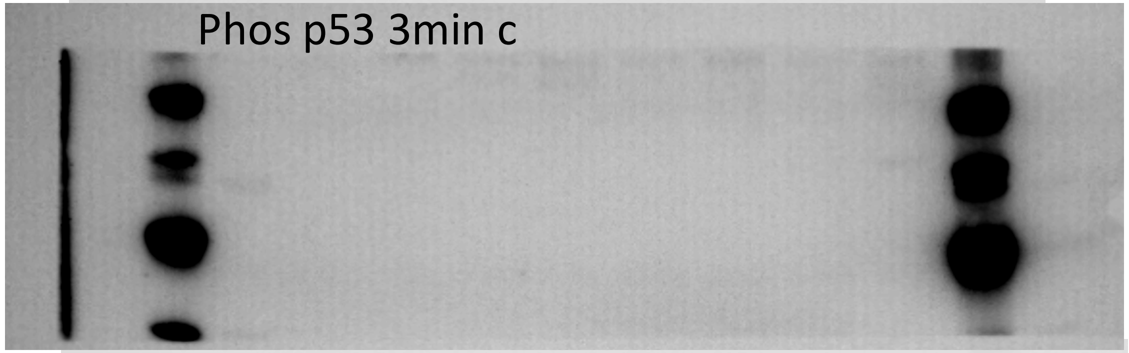

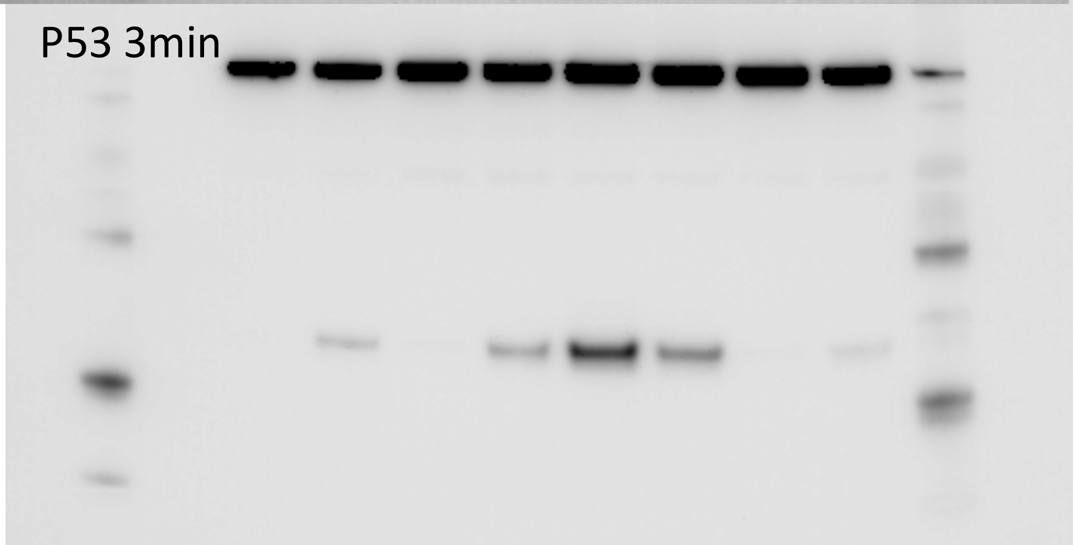

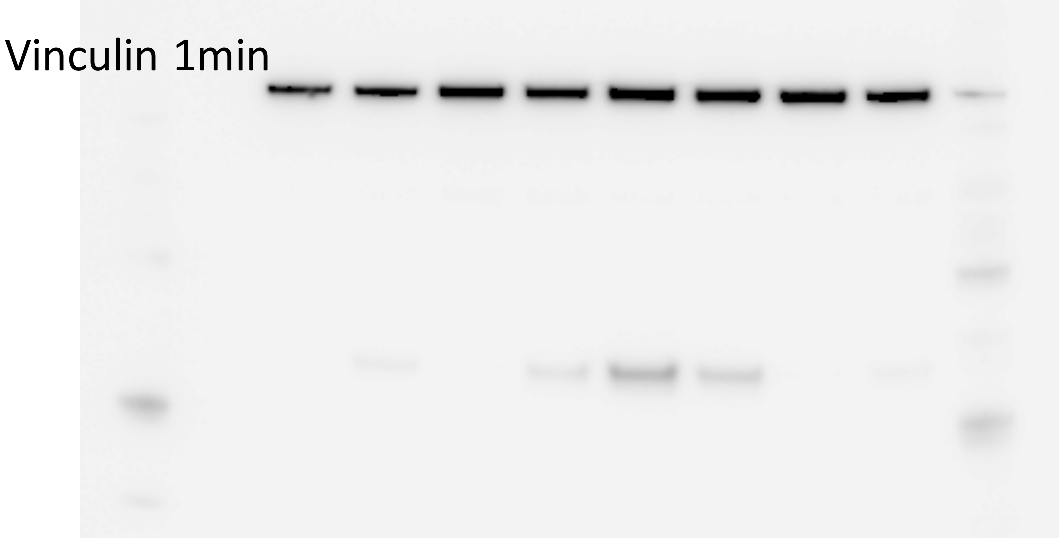

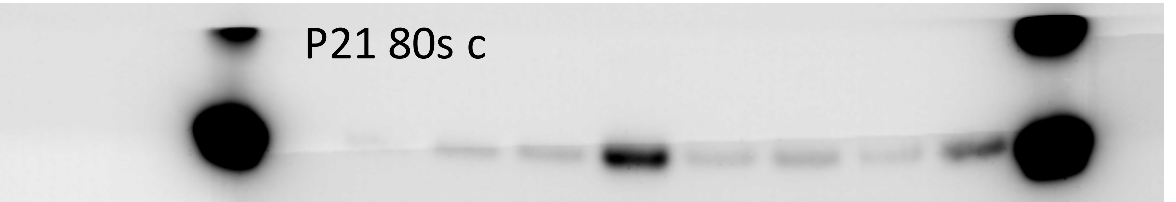

2021-DT-08: AS phos p38, p38, vinculin;48-72h

Loading:

Laddr	Laddr	48h	48h	48h	48h	72h	72h	72h	72 h	Laddr	Laddr
Magic Mark	Visual	Veh	SL-176	GSK-J4	Comb	Veh	SL-176	GSK-J4	Comb	Magic Mark	Visual

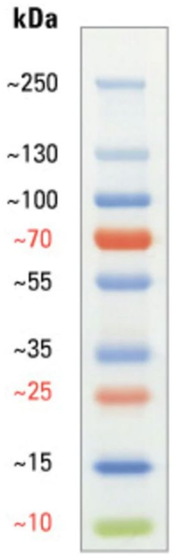

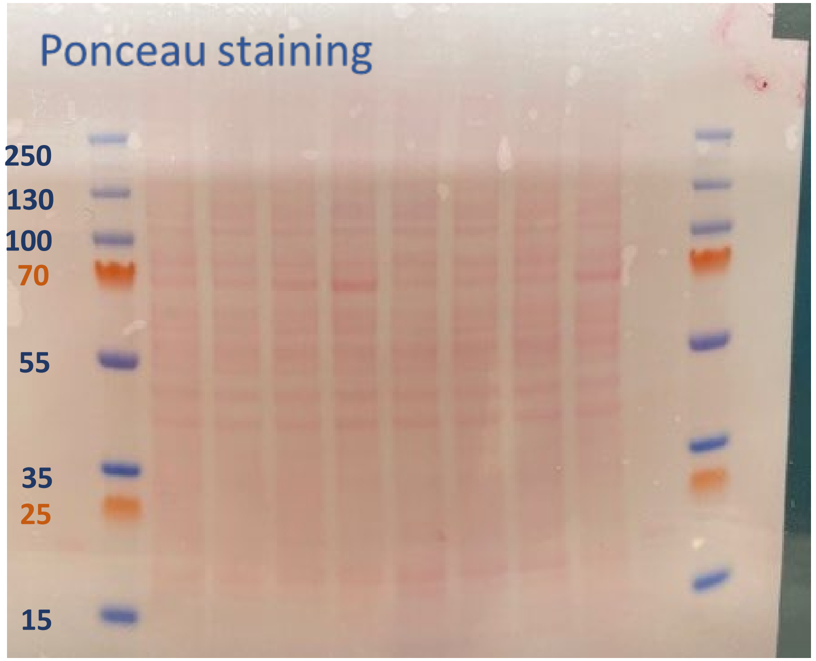

MagicMark

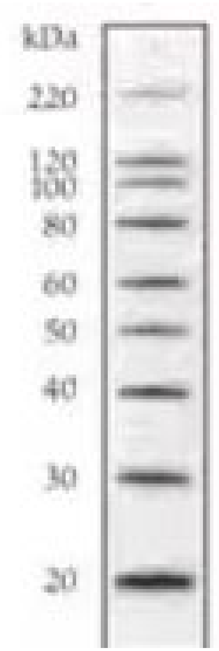

Vinculin 20s

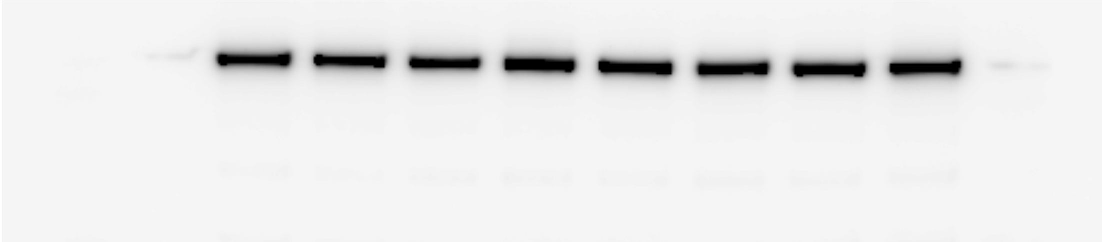

Phos p38 2min

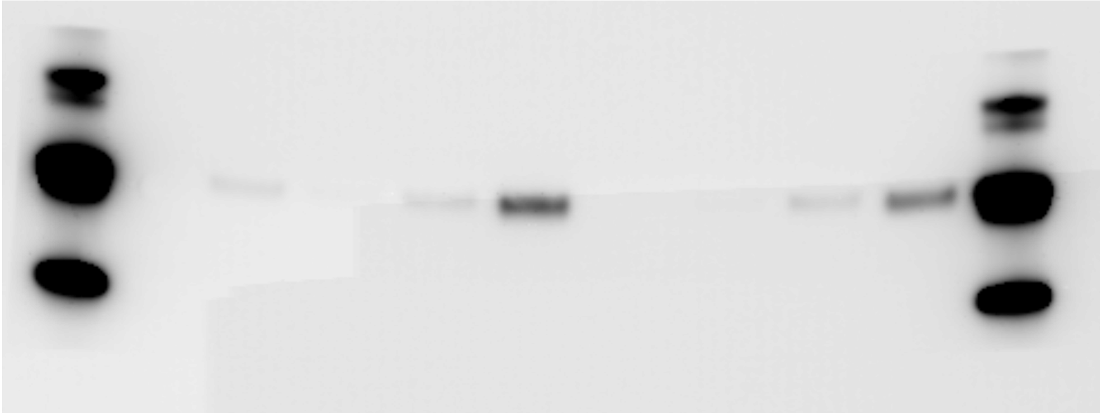

P38 20s

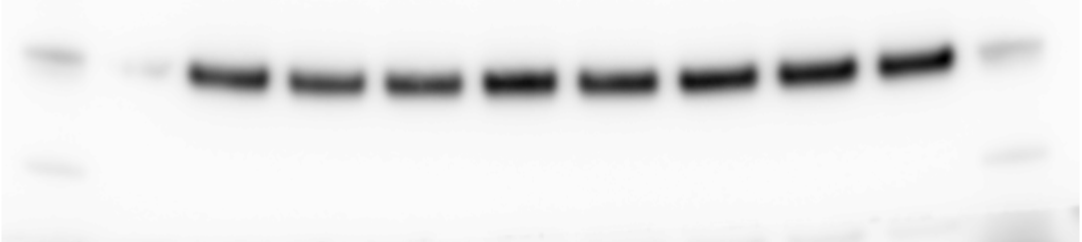

Blots for supplementary figure S4A

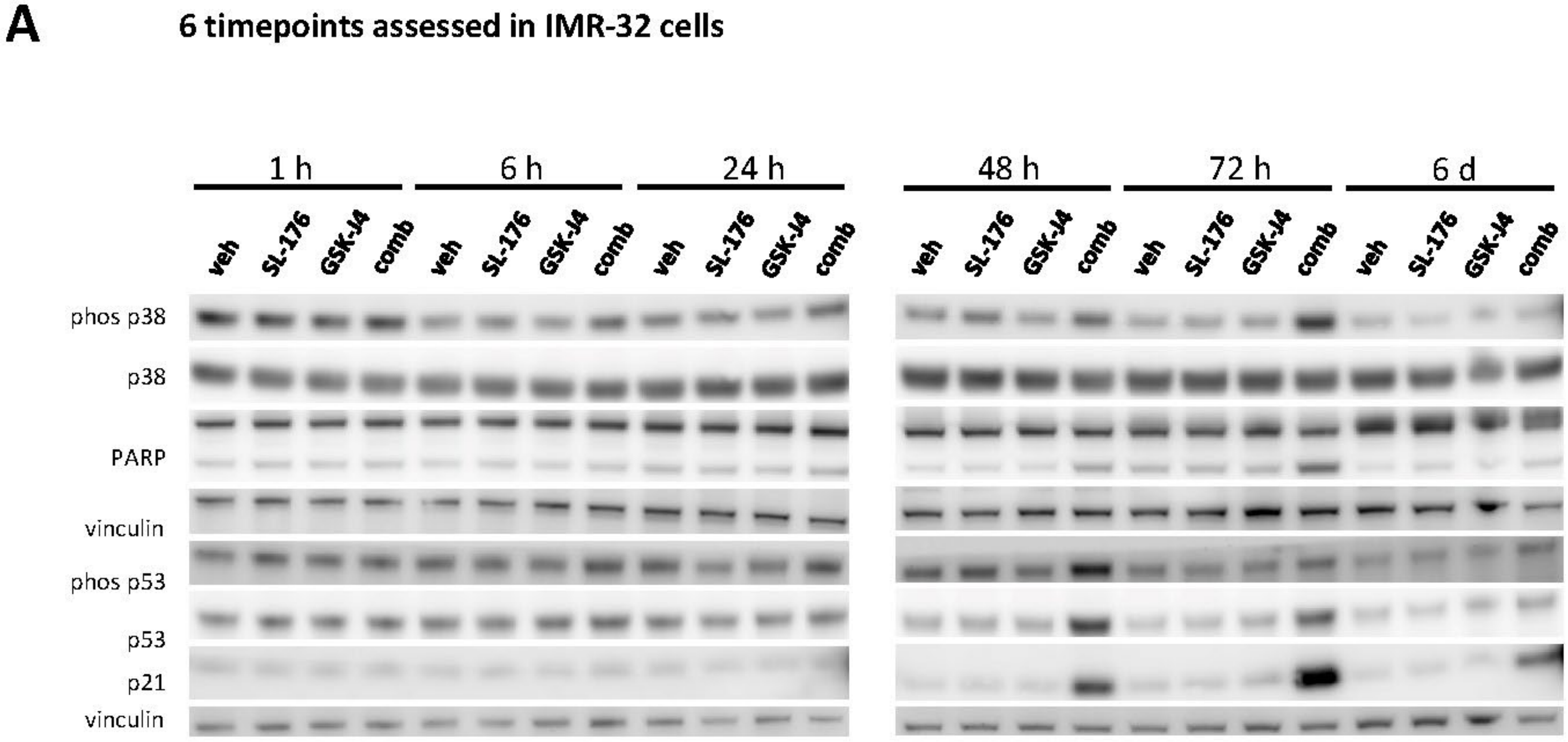

2020-DT-09: IMR-32 phos p38, p38, vinculin; 1-6 -24h

Loading:

Laddr	Laddr	1 h	1 h	1 h	1 h	6 h	6 h	6 h	6 h	24 h	24 h	24 h	24 h	Laddr
Magic Mark	Visual	Veh	SL-176	GSK-J4	Comb	Veh	SL-176	GSK-J4	Comb	Veh	SL-176	GSK-J4	comb	Magic Mark

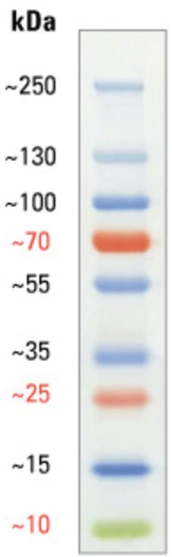

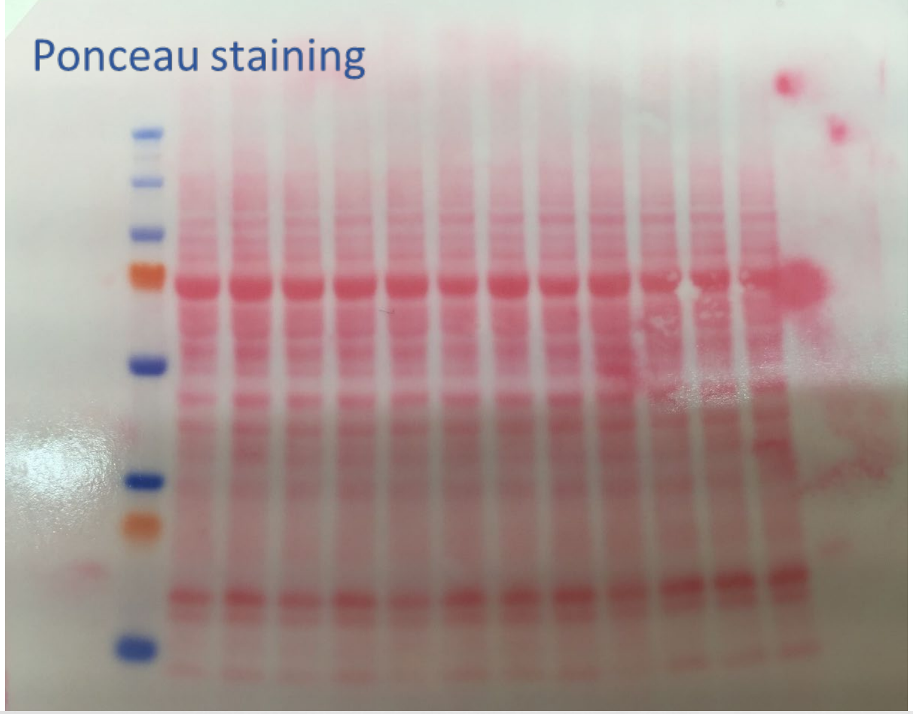

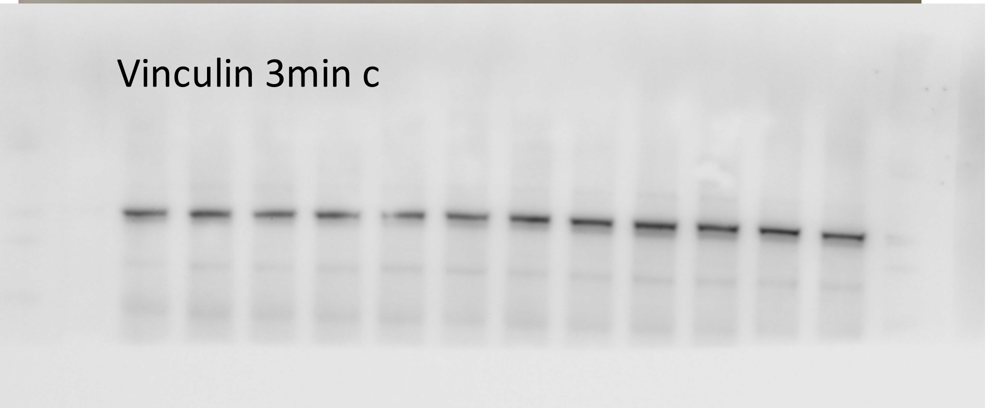

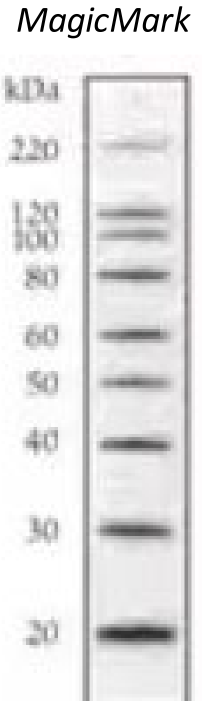

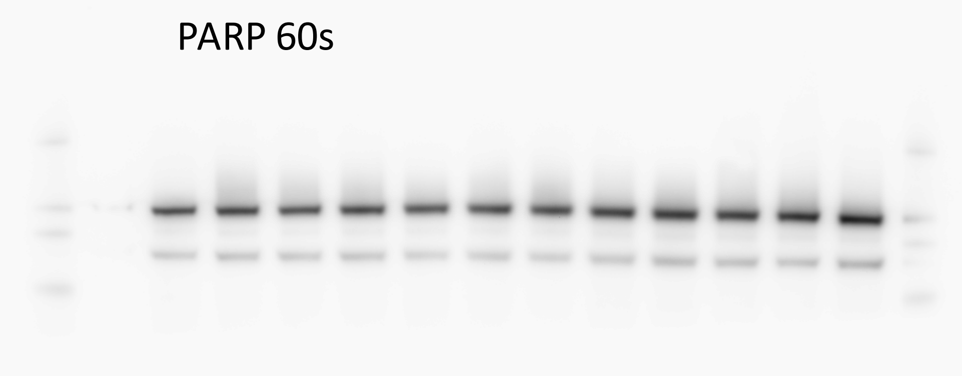

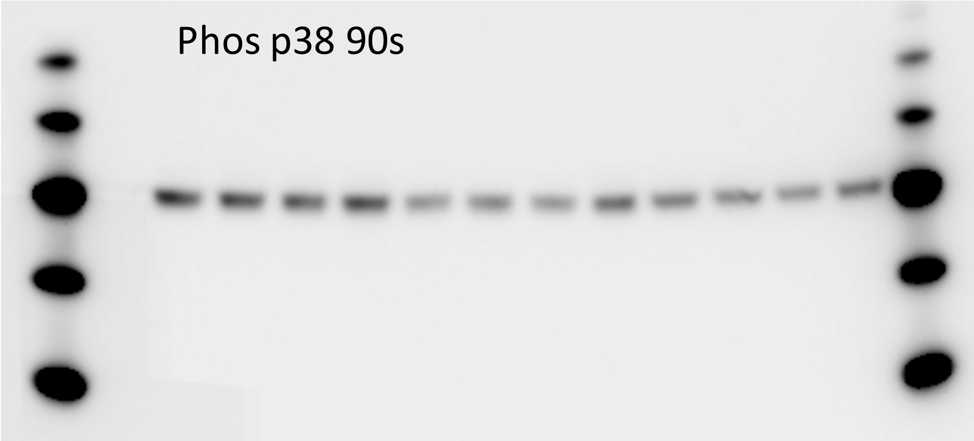

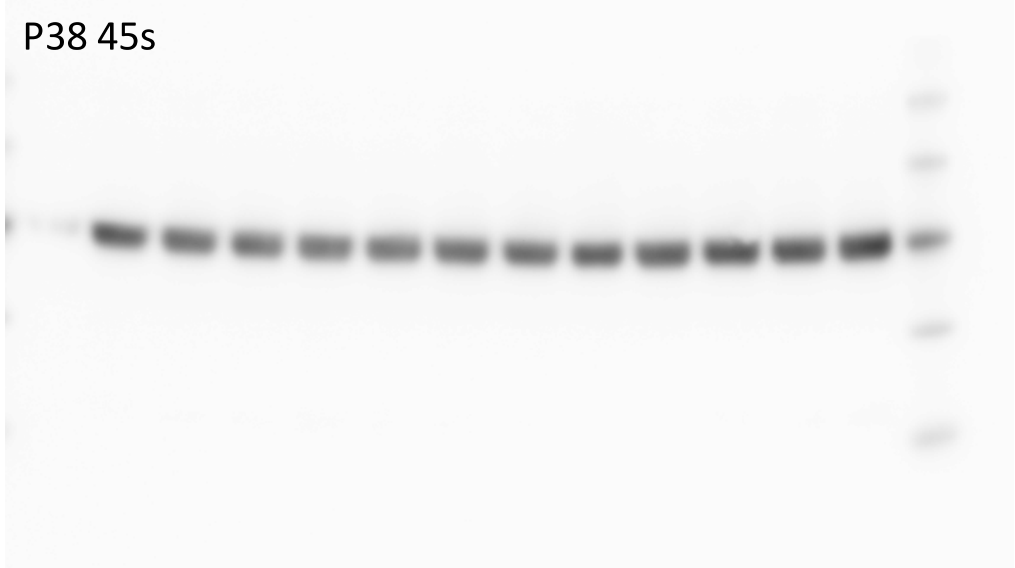

2020-DT-11: IMR-32 phos p53, p53, p21 vinculin; 1 - 6 - 24 h.

Laddr	Laddr	1 h	1 h	1 h	1 h	6 h	6 h	6 h	6 h	24 h	24 h	24 h	24 h	Laddr
Magic Mark	Visual	Veh	SL-176	GSK-J4	Comb	Veh	SL-176	GSK-J4	Comb	Veh	SL-176	GSK-J4	comb	Magic Mark

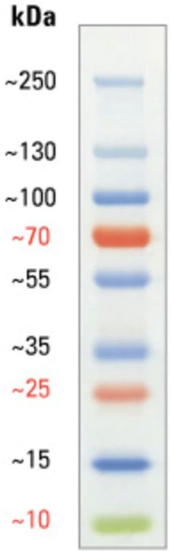

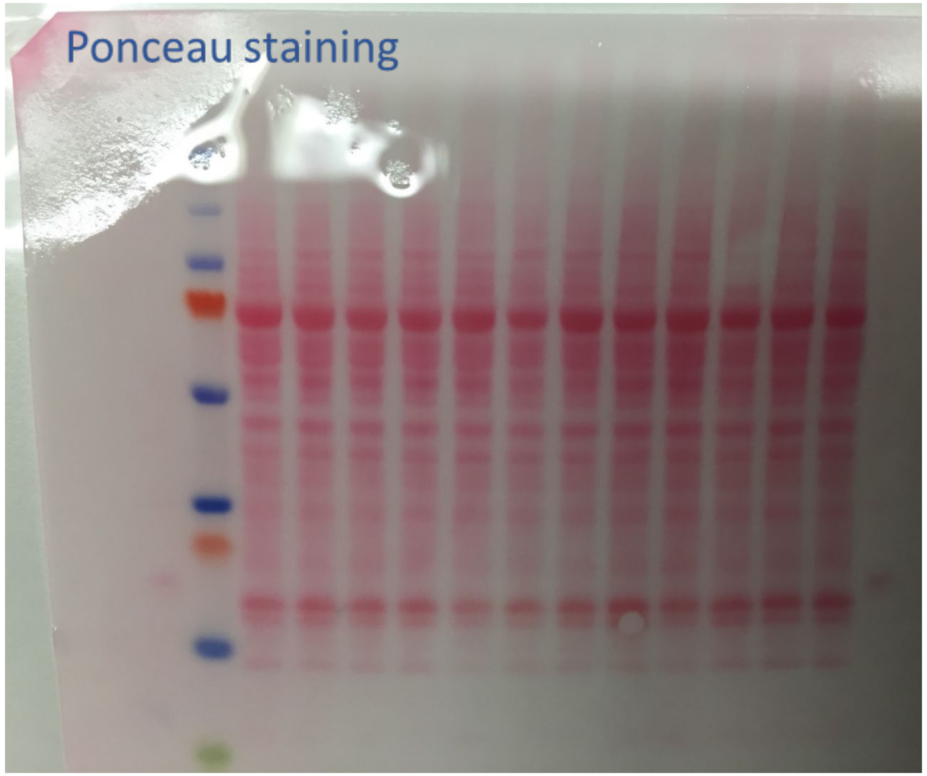

MagicMark

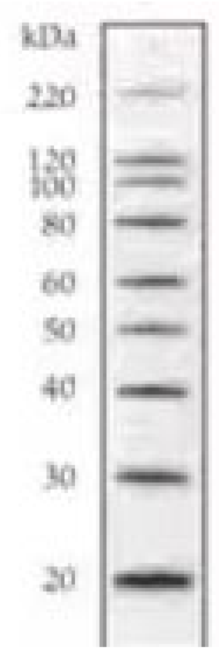

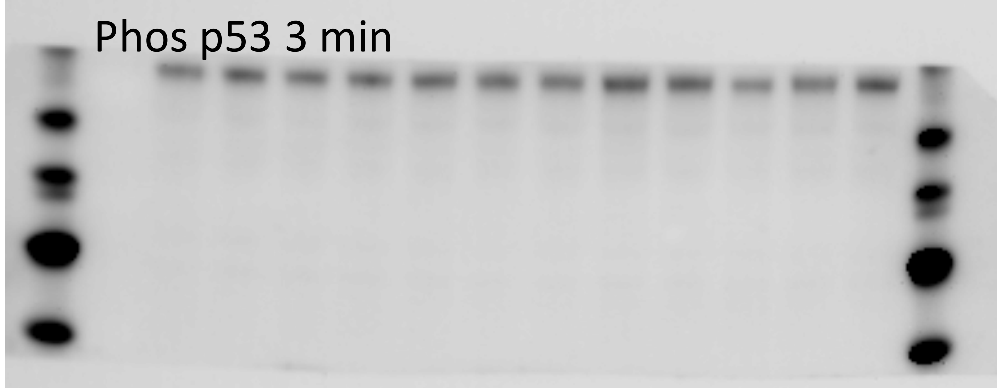

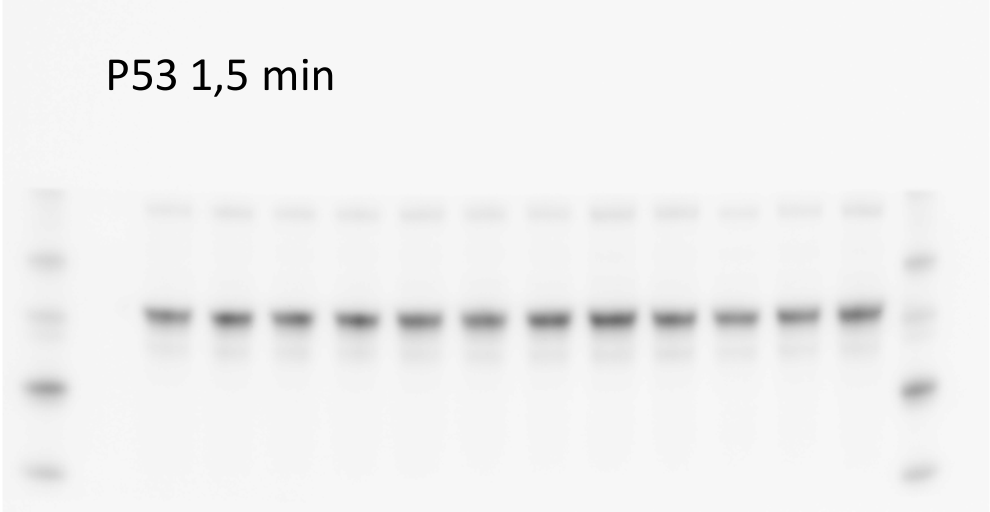

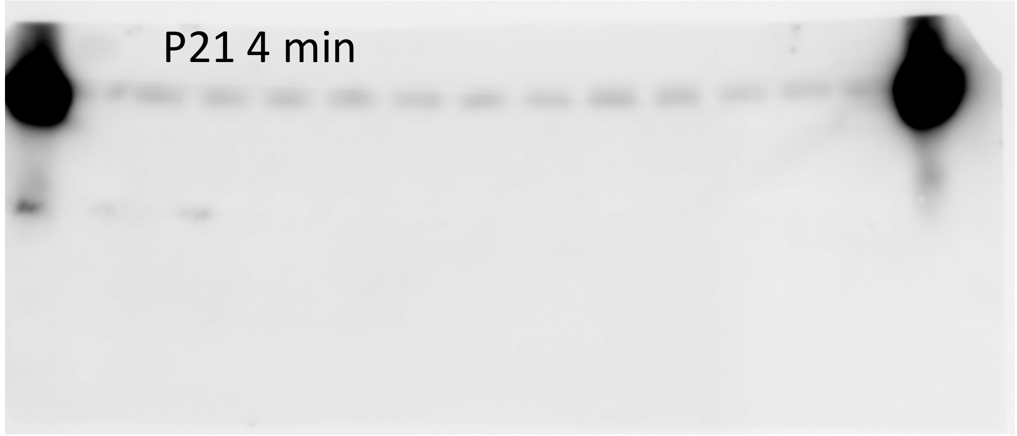

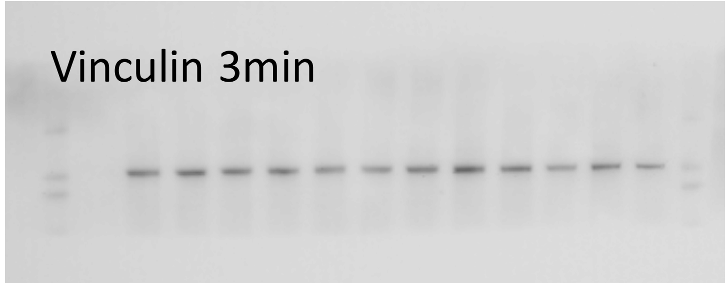

2020-DT-10:IMR-32 PARP, phos p38, p38, vinculin;48h - 72h – 6d

Loading:

Laddr	Laddr	48h	48h	48h	48h	72h	72h	72h	72 h	6 d	6 d	6 d	6 d	Laddr
Magic Mark	Visual	Veh	SL-176	GSK-J4	Comb	Veh	SL-176	GSK-J4	Comb	Veh	SL-176	GSK-J4	comb	Magic Mark

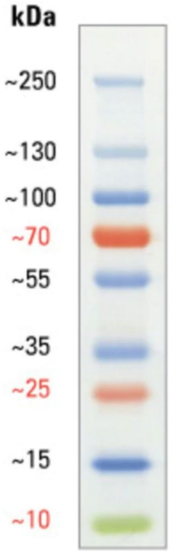

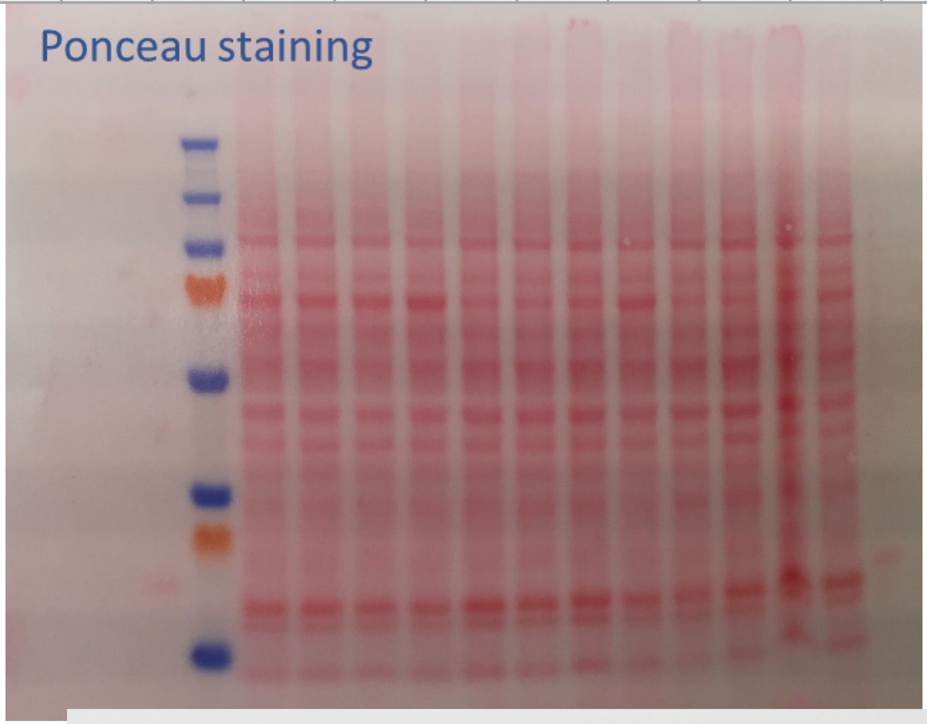

MagicMark

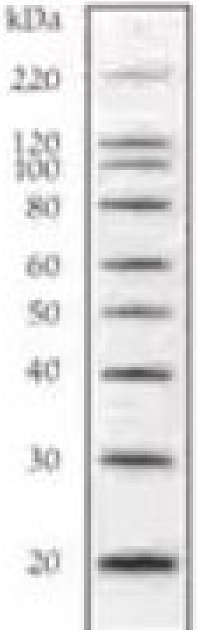

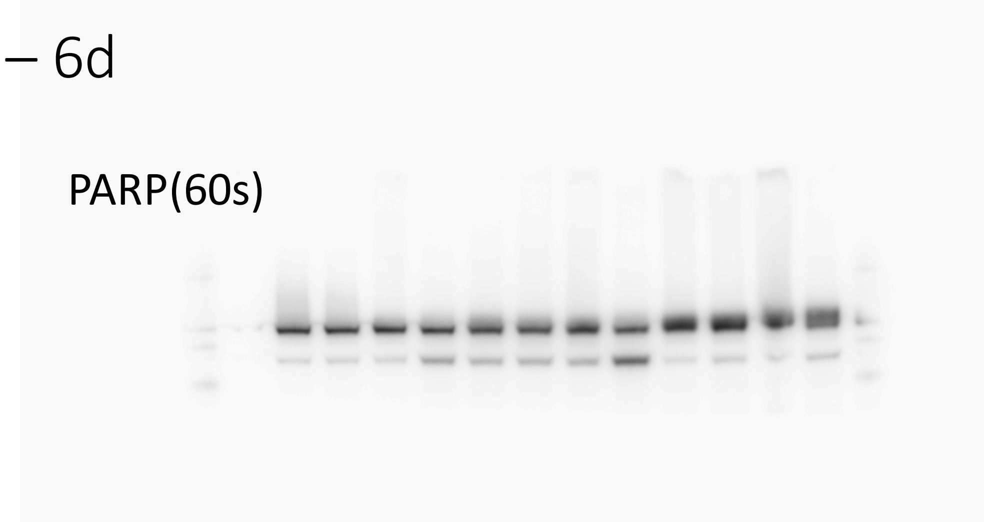

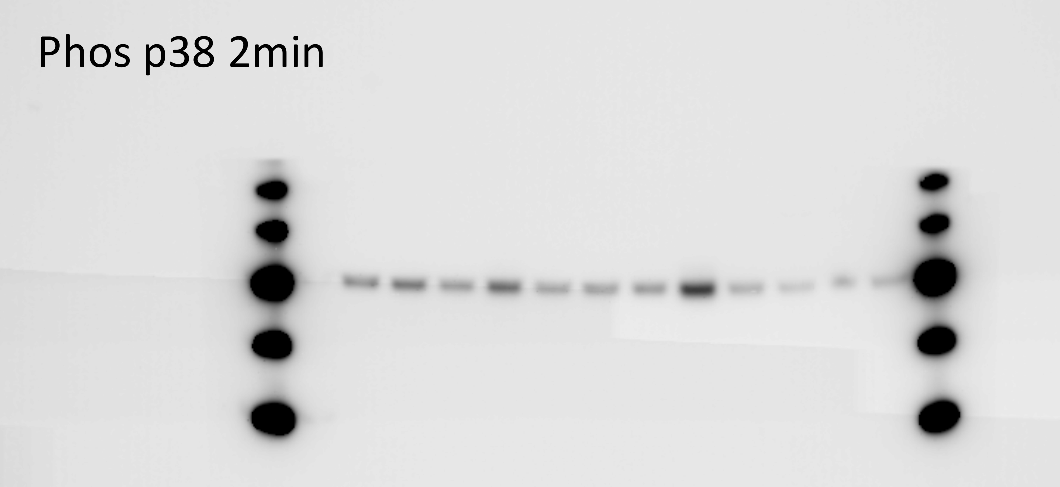

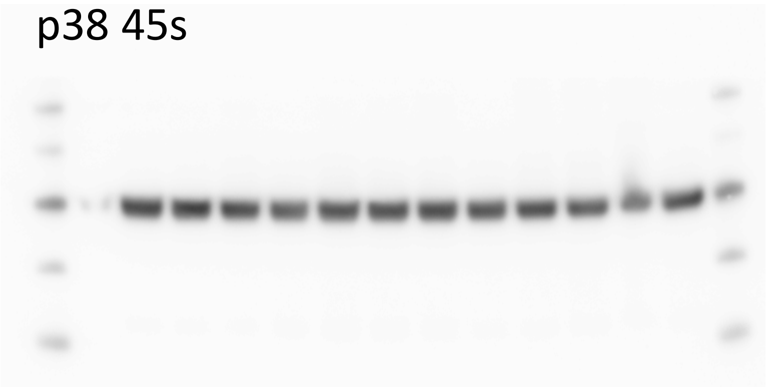

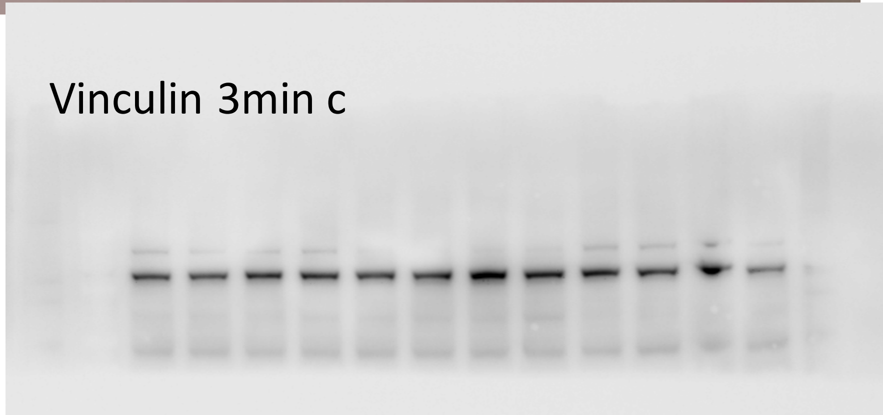

2020-DT-12: IMR-32 phos p53, p53, p21, vinculin;48-72h-6d

Loading:

Laddr	Laddr	48h	48h	48h	48h	72h	72h	72h	72 h	6 d	6 d	6 d	6 d	Laddr
Magic Mark	Visual	Veh	SL-176	GSK-J4	Comb	Veh	SL-176	GSK-J4	Comb	Veh	SL-176	GSK-J4	comb	Magic Mark

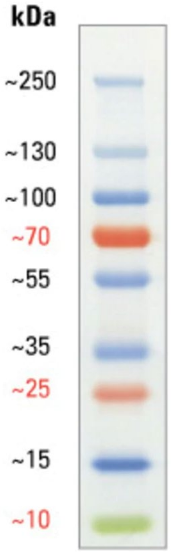

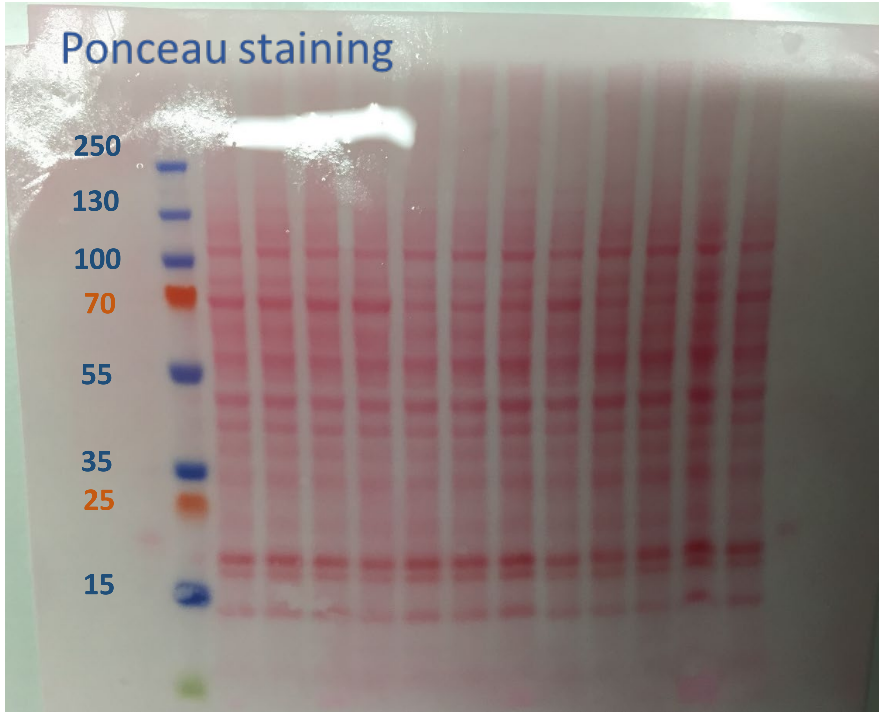

MagicMark

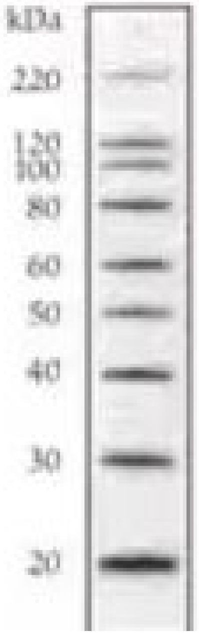

Phos p53 4min

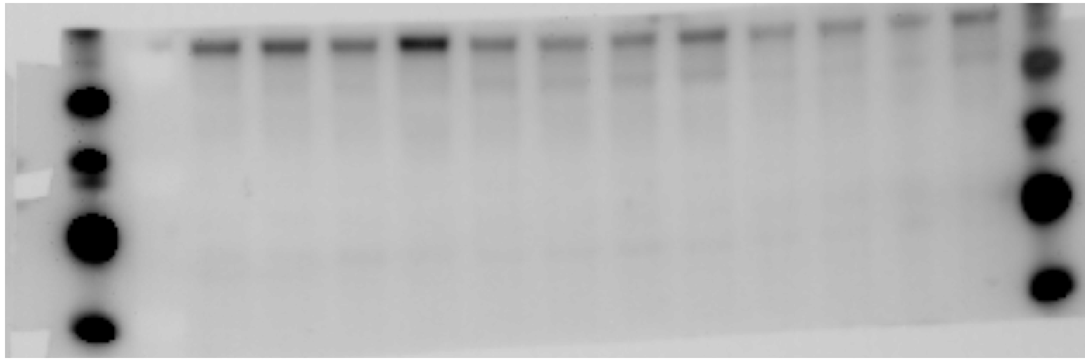

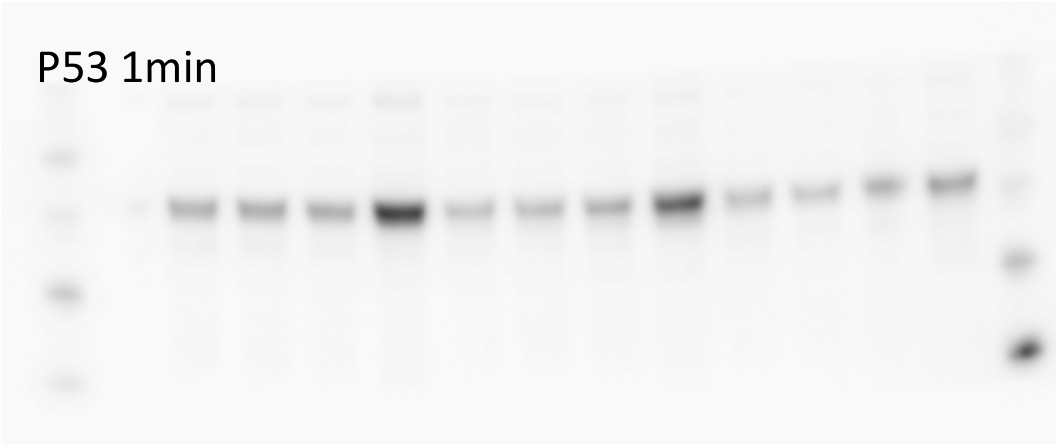

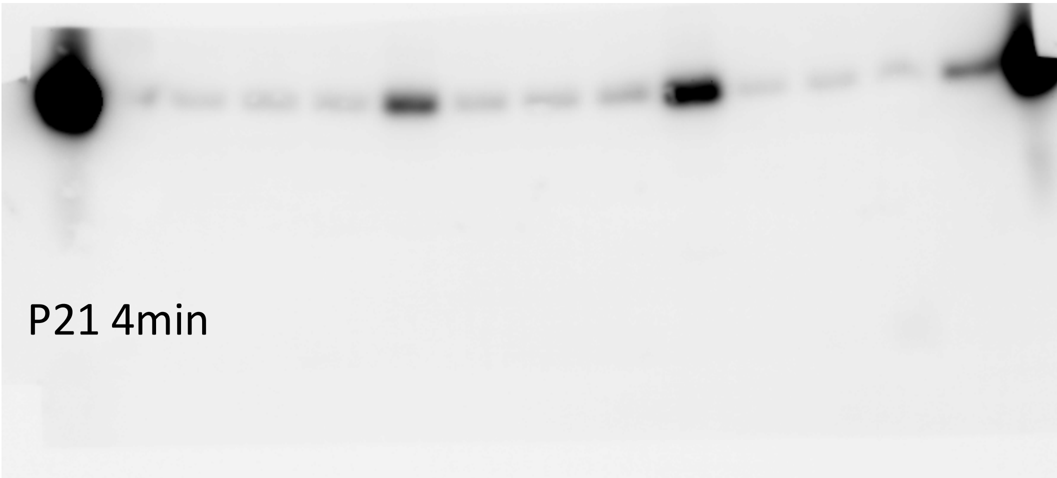

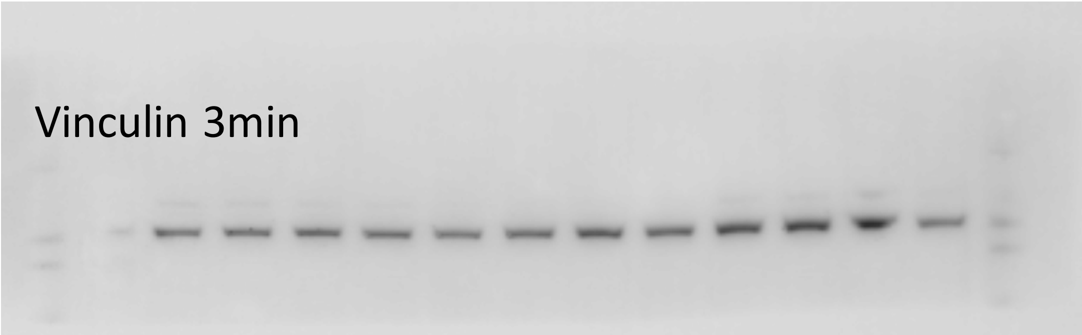

Blots for supplementary figure S4B

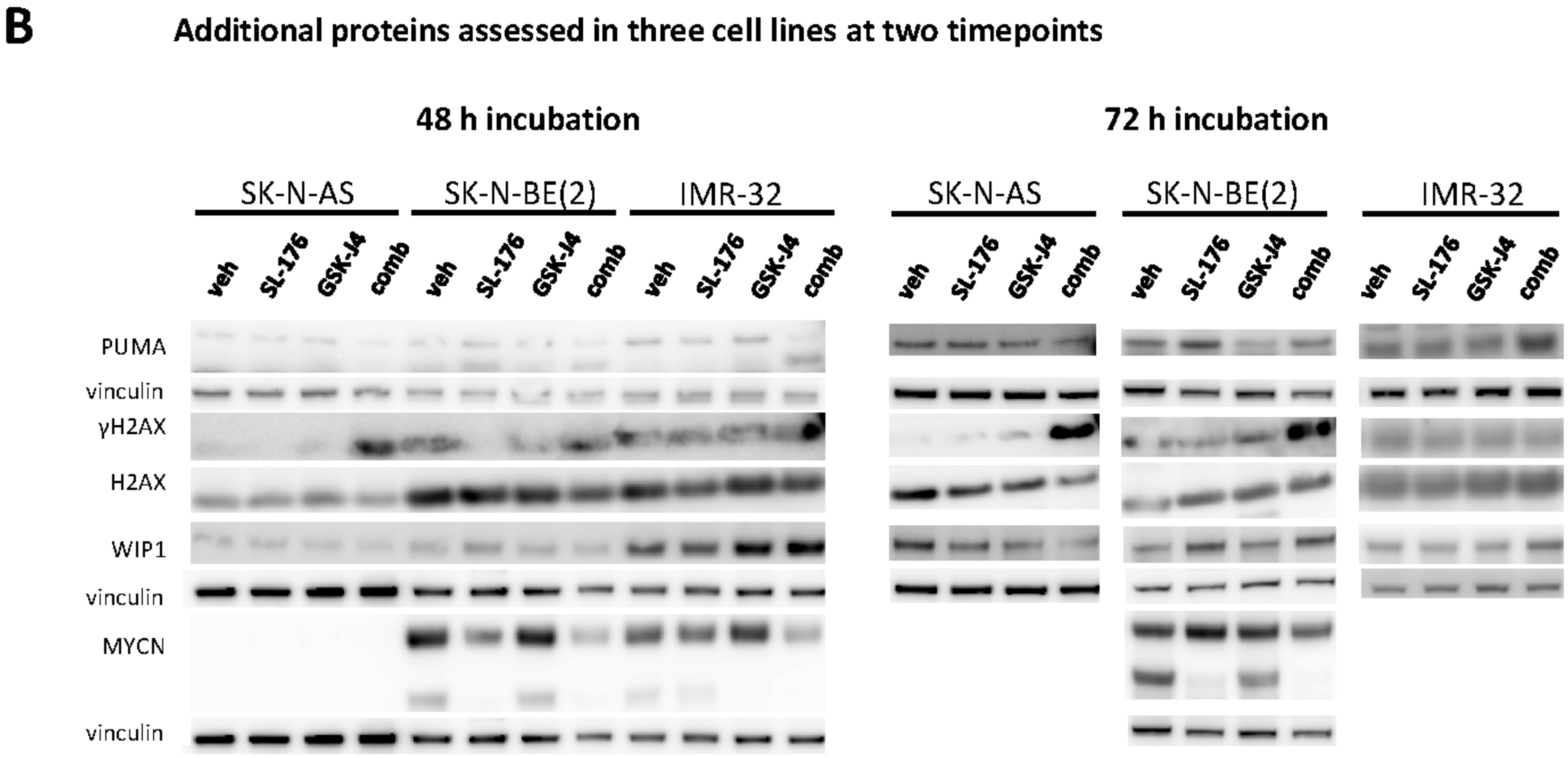

2021-DT-04: 3 cell lines (SK-N-AS, SK-N-BE(2), IMR-32); PUMA, vinculin; 48h incubation

Loading:

Laddr	Laddr	AS	AS	AS	AS	BE(2)	BE(2)	BE(2)	BE(2)	IMR	IMR	IMR	IMR	Laddr
Magic Mark	Visual	Veh	SL-176	GSK-J4	Comb	Veh	SL-176	GSK-J4	Comb	Veh	SL-176	GSK-J4	comb	Magic Mark

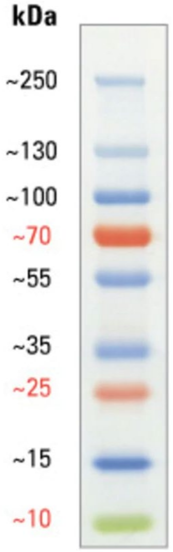

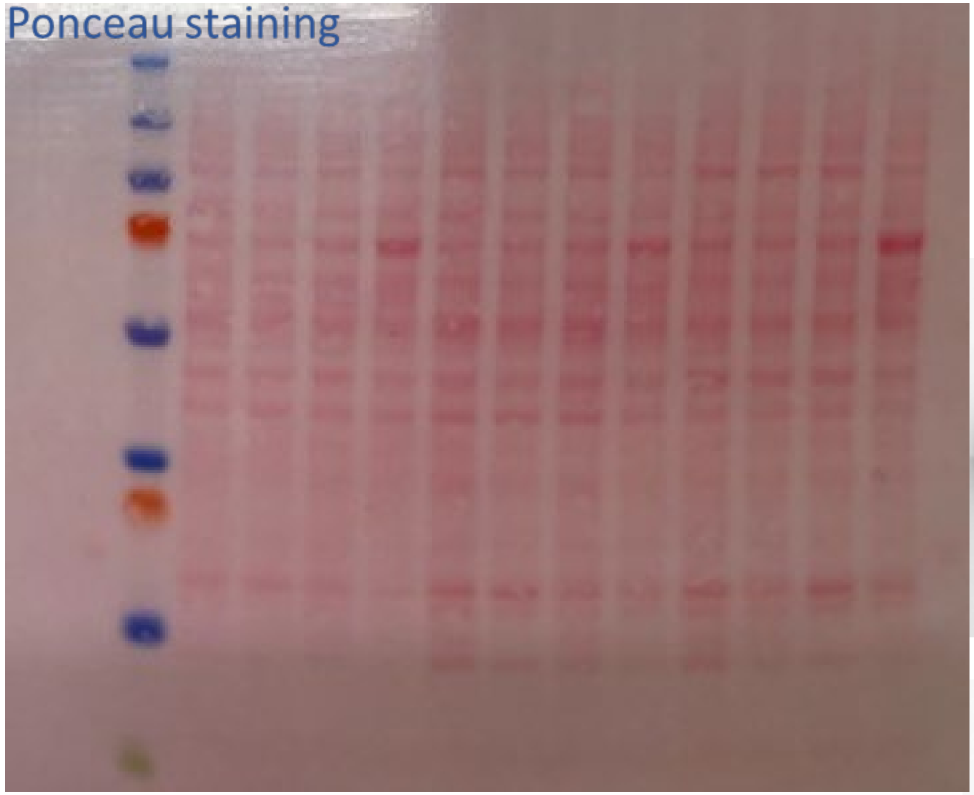

MagicMark

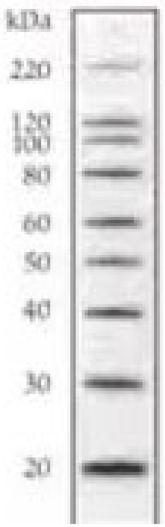

PUMA 2min

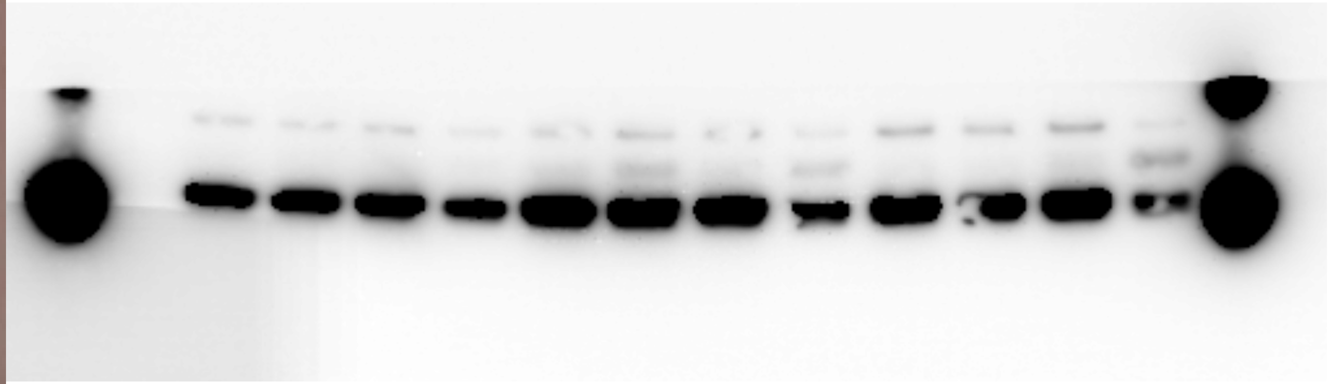

Vinculin 90s

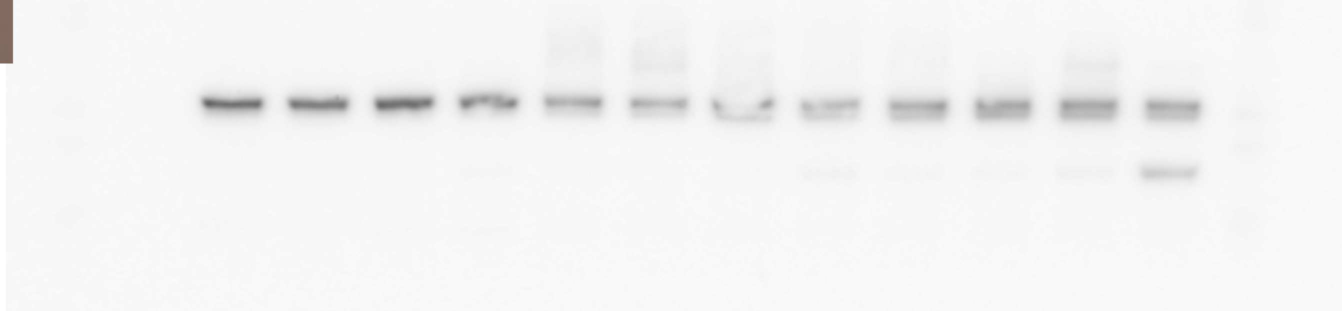

2021-DT-03: 3 cell lines (SK-N-AS, SK-N-BE(2), IMR-32); γH2AX, H2AX, WIP1, MYCN, vinculin;48h

Loading:

Laddr	Laddr	AS	AS	AS	AS	BE(2)	BE(2)	BE(2)	BE(2)	IMR	IMR	IMR	IMR	Laddr
Magic Mark	Visual	Veh	SL-176	GSK-J4	Comb	Veh	SL-176	GSK-J4	Comb	Veh	SL-176	GSK-J4	comb	Magic Mark

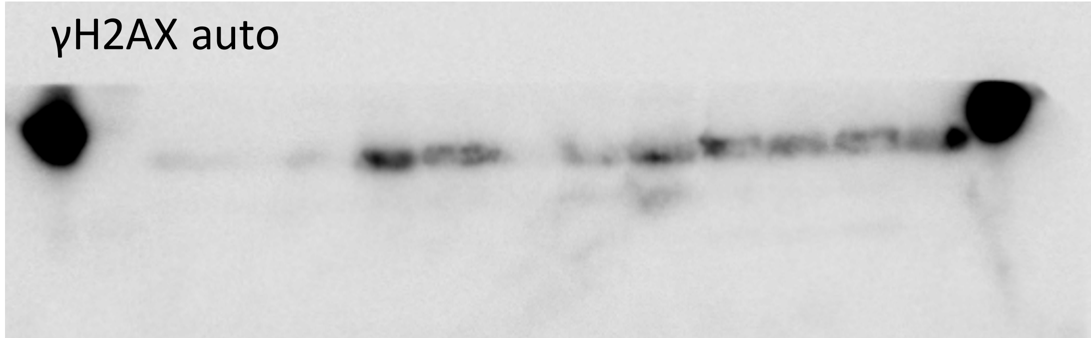

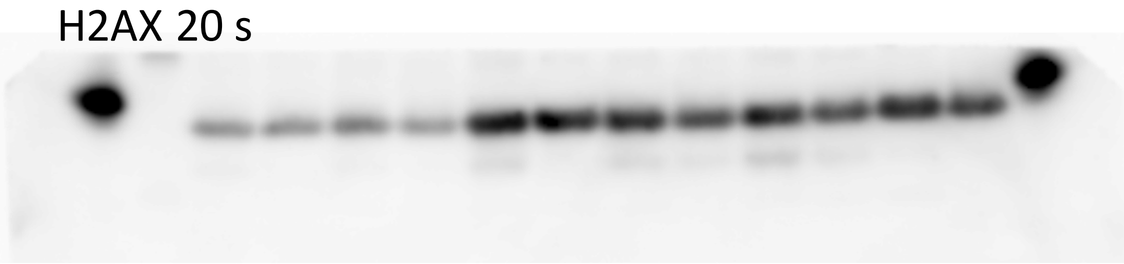

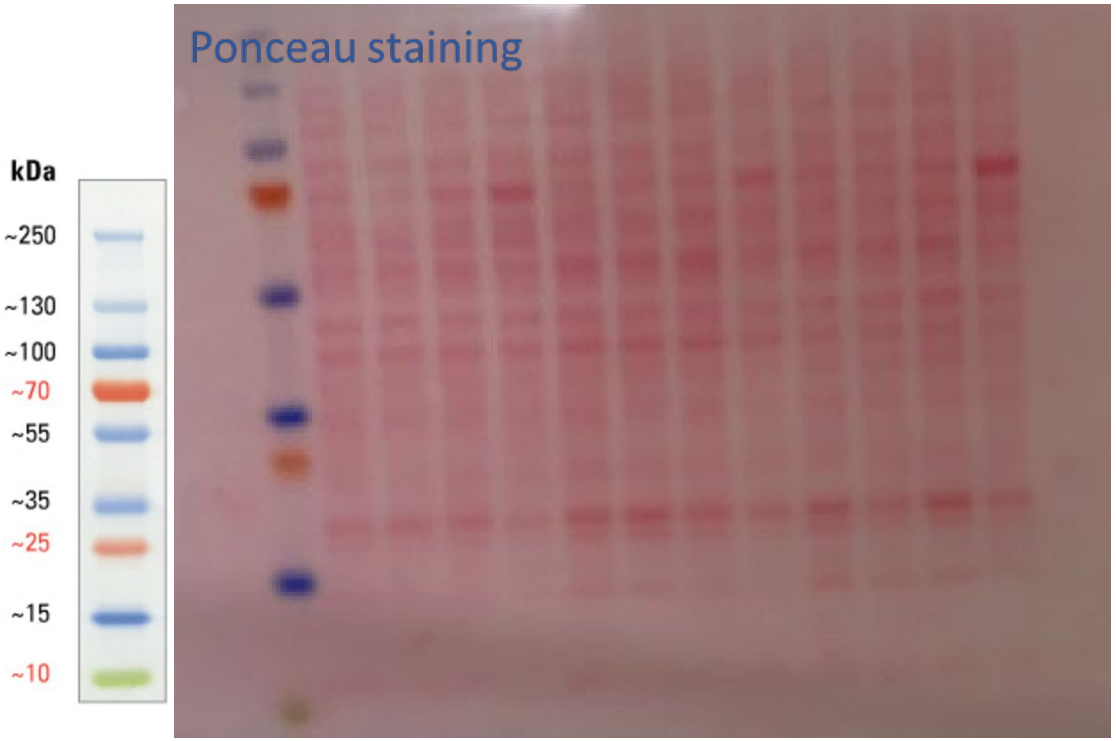

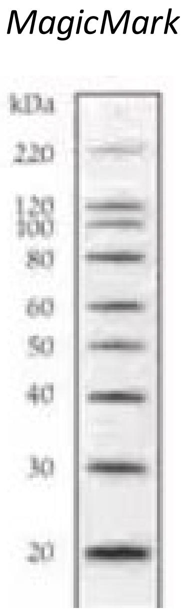

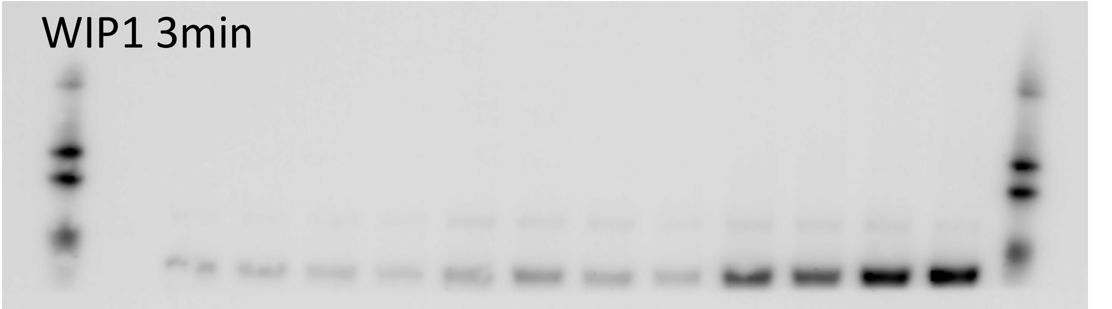

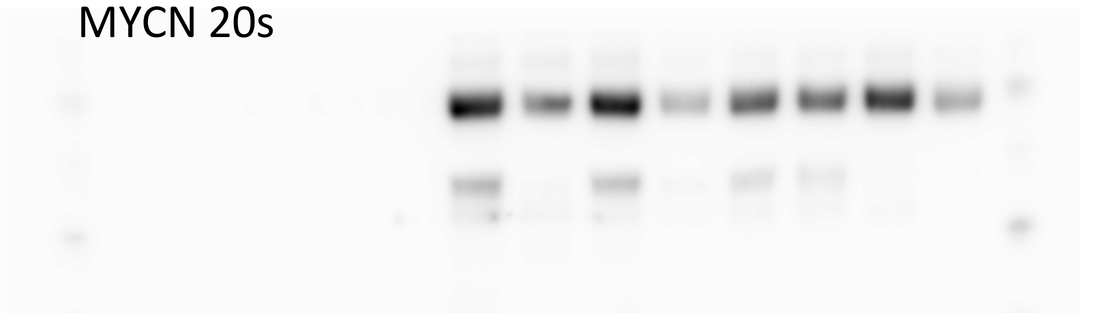

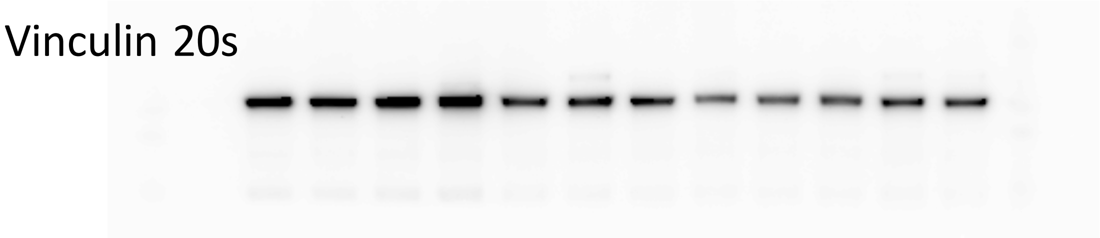

2021-DT-07: SK-N-AS cells; PUMA, vinculin; 72h incubation

Loading:

Laddr	Laddr	48h	48h	48h	48h	72h	72h	72h	72 h	Laddr	Laddr
Magic Mark	Visual	Veh	SL-176	GSK-J4	Comb	Veh	SL-176	GSK-J4	Comb	Magic Mark	Visual

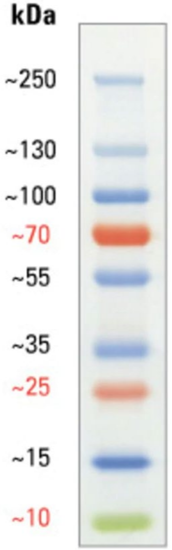

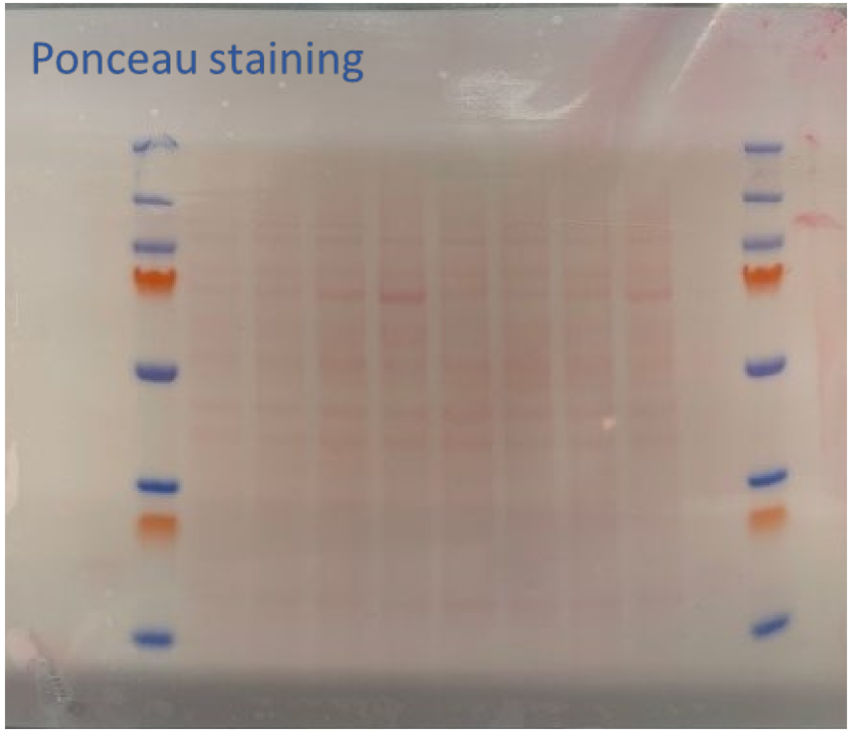

MagicMark

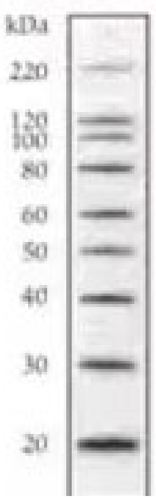

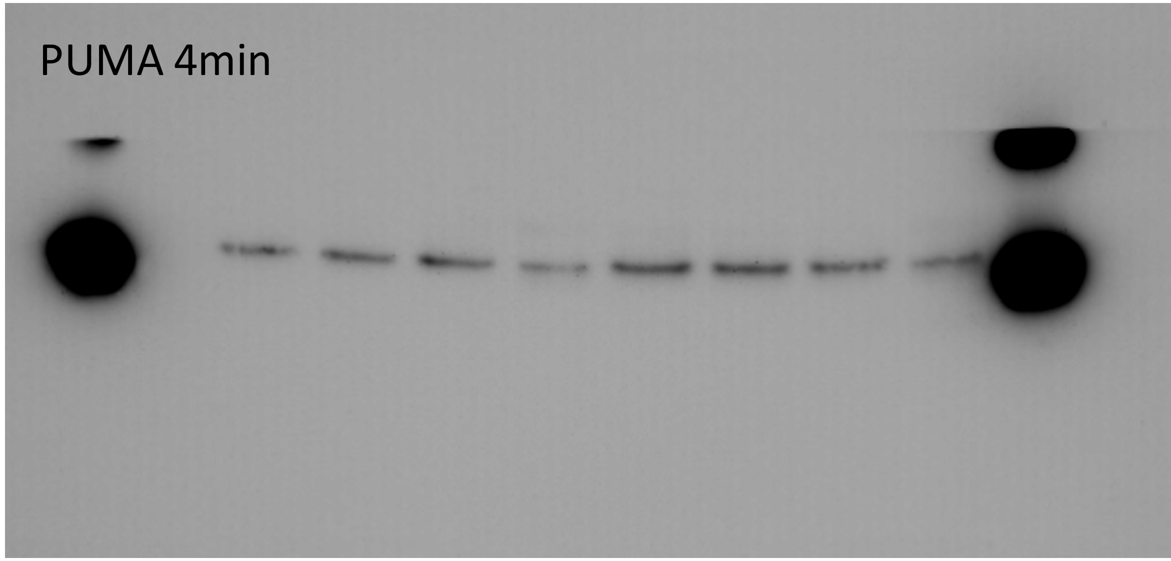

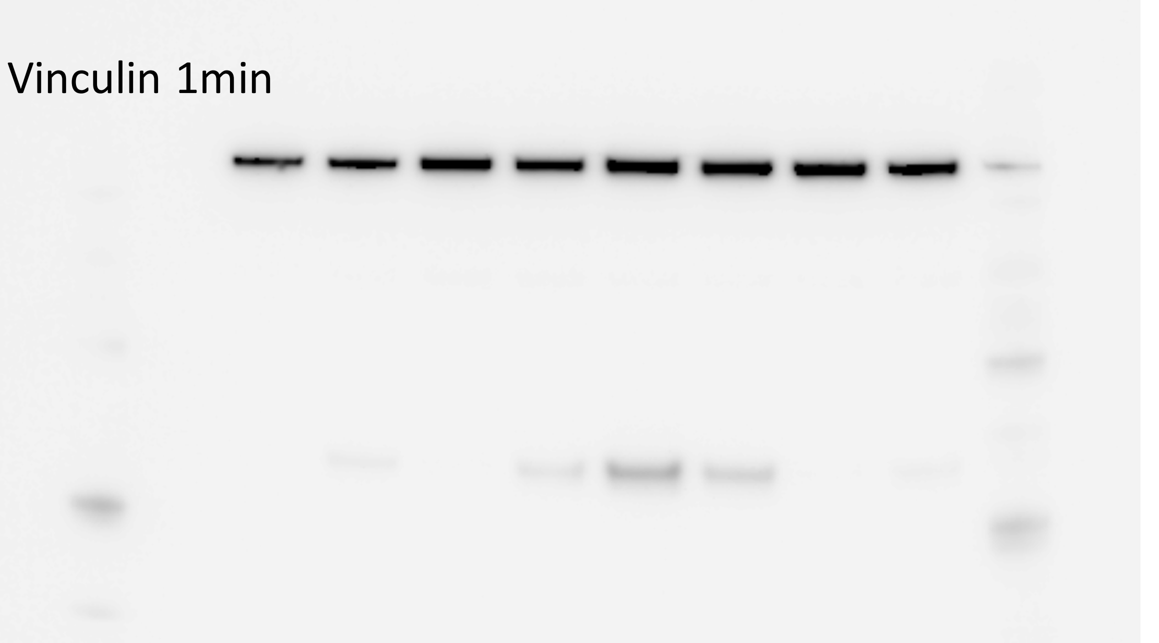

2021-DT-08: SK-N-AS cells; γH2AX, H2AX, WIP1, vinculin; 72h

Loading:

Laddr	Laddr	48h	48h	48h	48h	72h	72h	72h	72 h	Laddr	Laddr
Magic Mark	Visual	Veh	SL-176	GSK-J4	Comb	Veh	SL-176	GSK-J4	Comb	Magic Mark	Visual

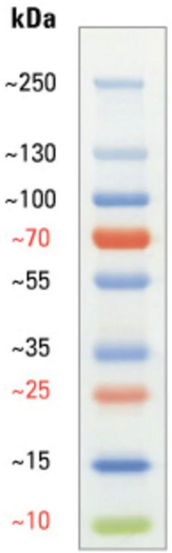

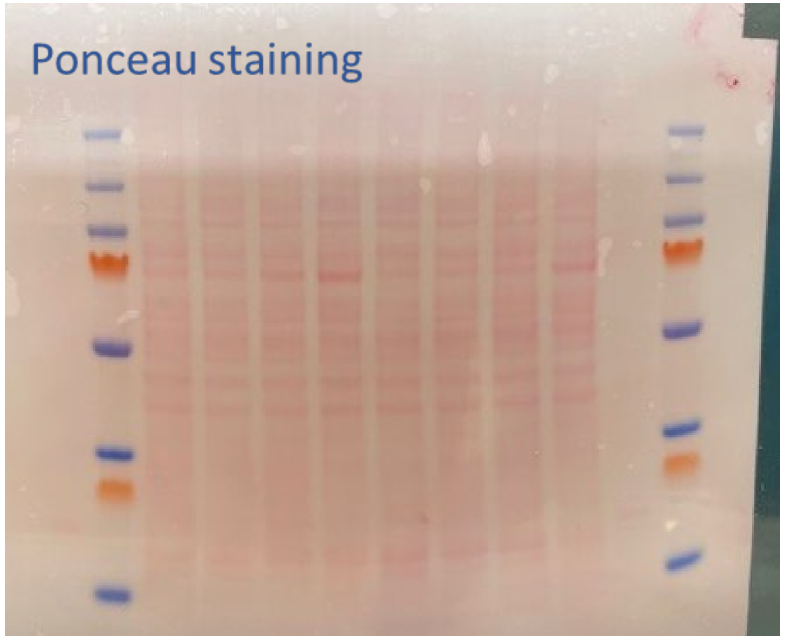

MagicMark

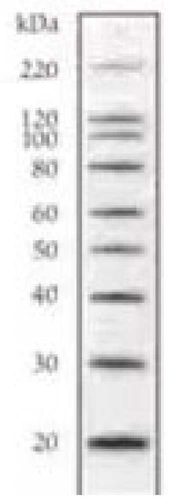

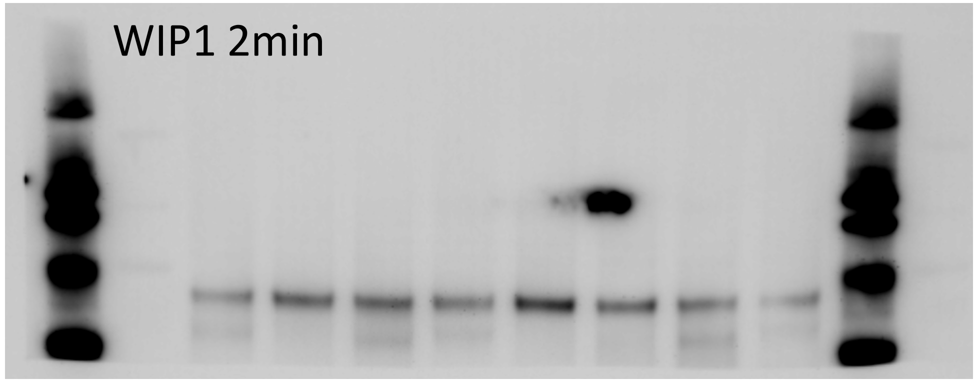

gammaH2AX 60s

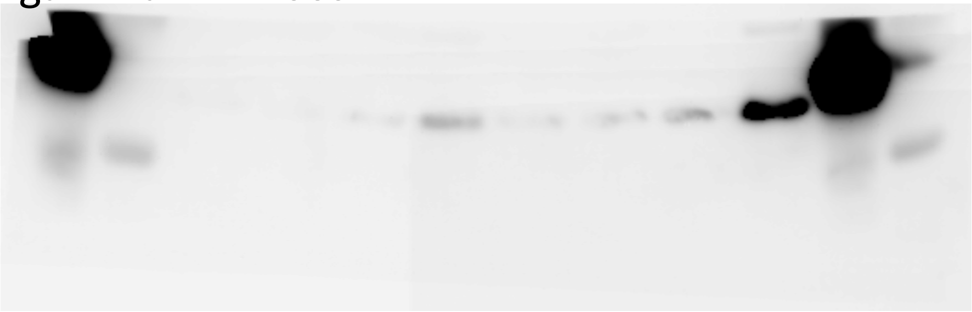

Vinculin 20s

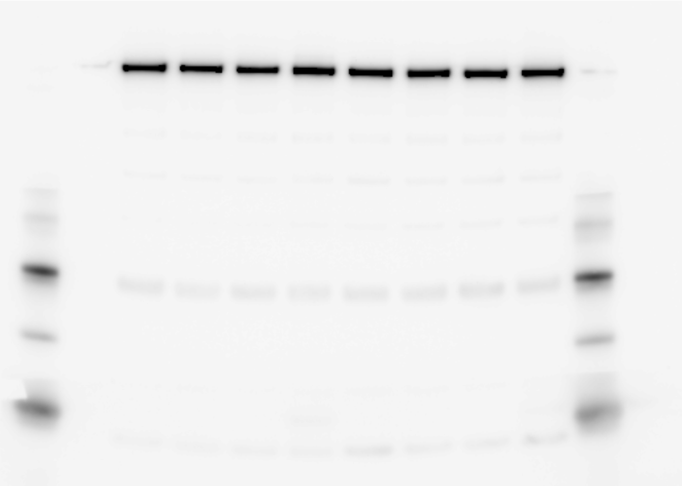

H2AX 40 s

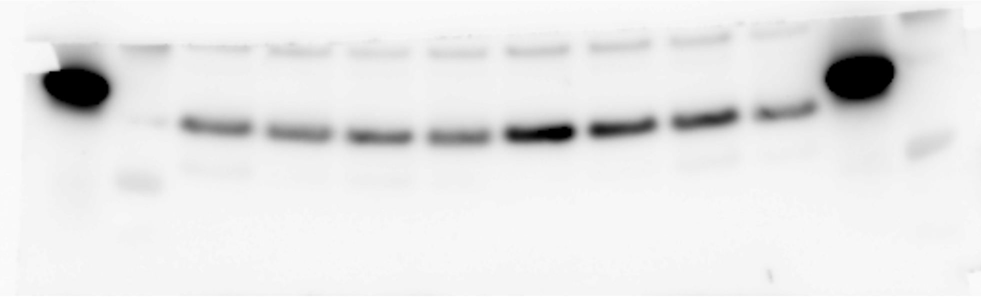

2021-DT-05: SK-N-BE(2) cells; PUMA, MYCN, vinculin; 72h

Loading:

Laddr	Laddr	48h	48h	48h	48h	72h	72h	72h	72 h	Laddr	Laddr
Magic Mark	Visual	Veh	SL-176	GSK-J4	Comb	Veh	SL-176	GSK-J4	Comb	Magic Mark	Visual

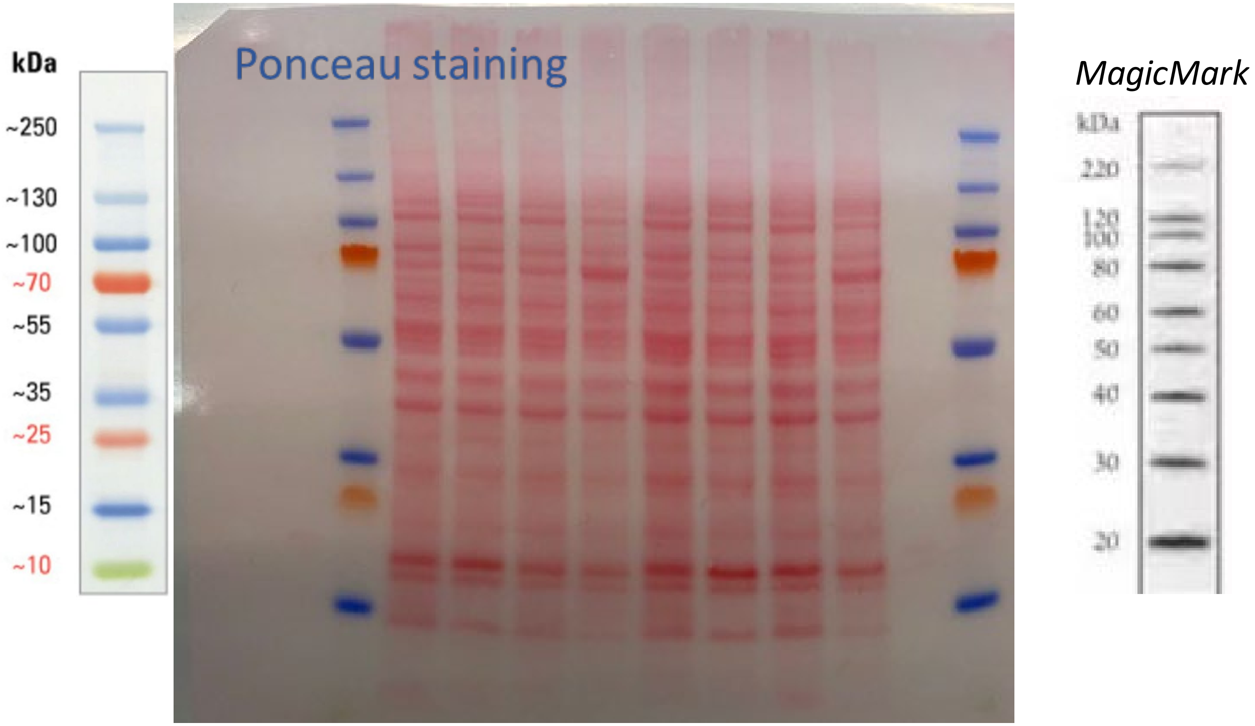

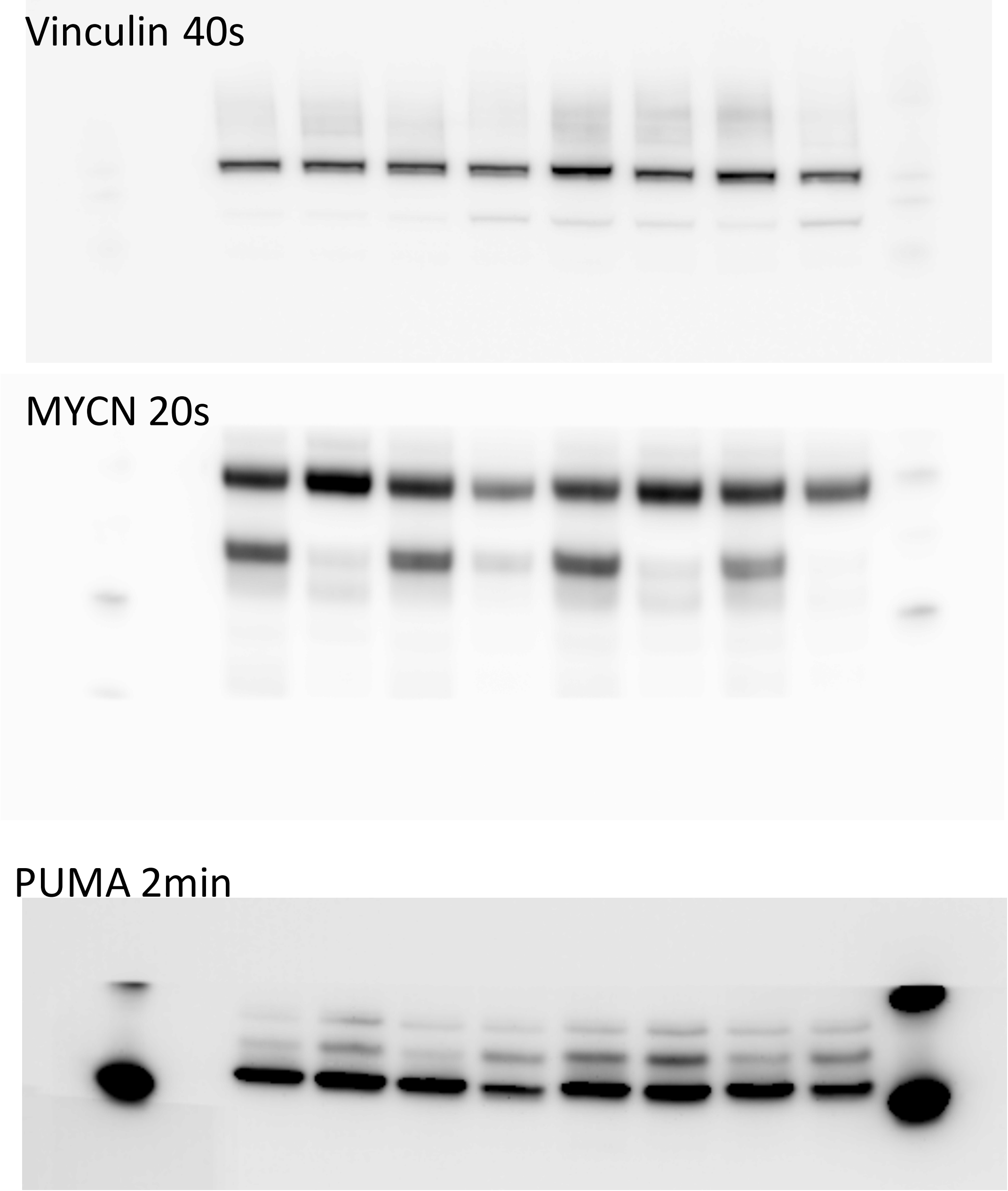

2021-DT-06: SK-N-BE(2) cells; γH2AX, H2AX, WIP1, vinculin; 72h

Loading:

Laddr	Laddr	48h	48h	48h	48h	72h	72h	72h	72 h	Laddr	Laddr
Magic Mark	Visual	Veh	SL-176	GSK-J4	Comb	Veh	SL-176	GSK-J4	Comb	Magic Mark	Visual

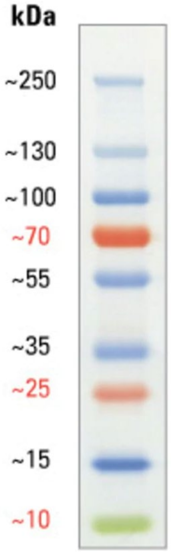

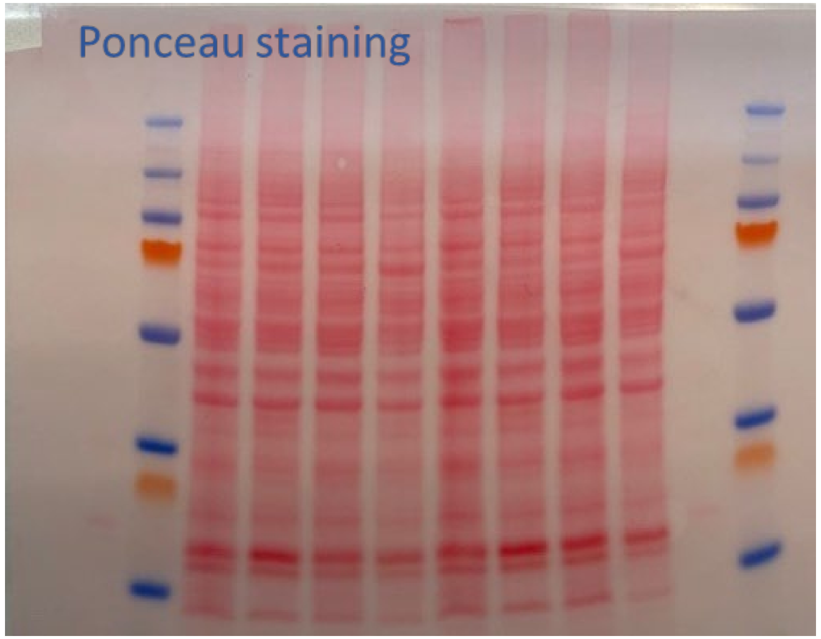

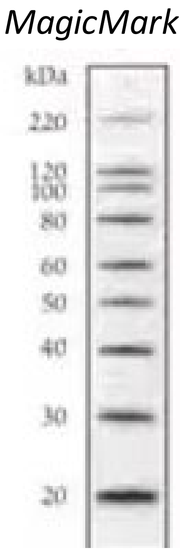

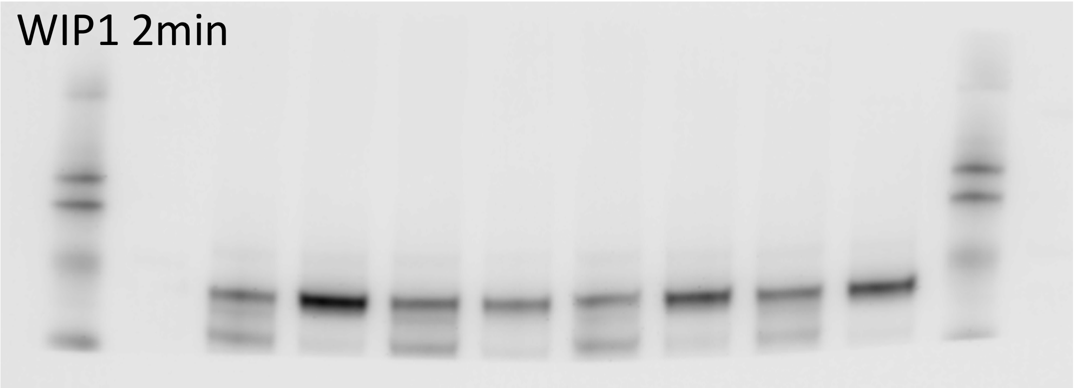

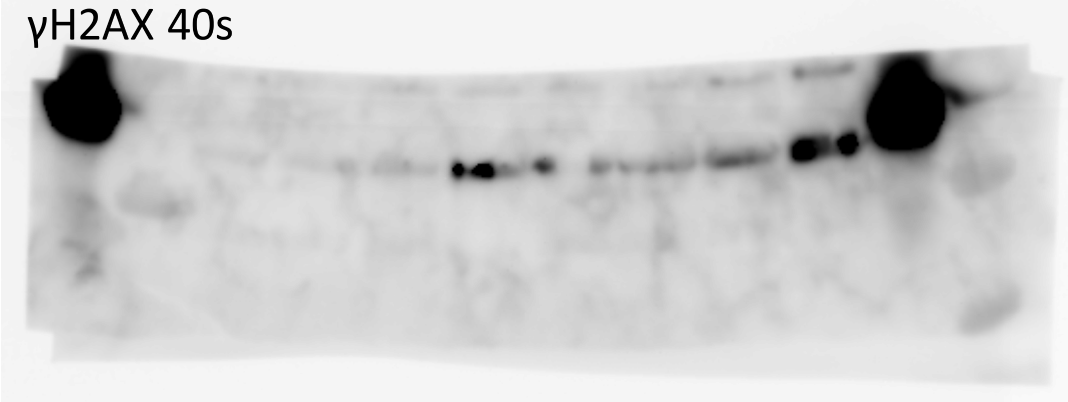

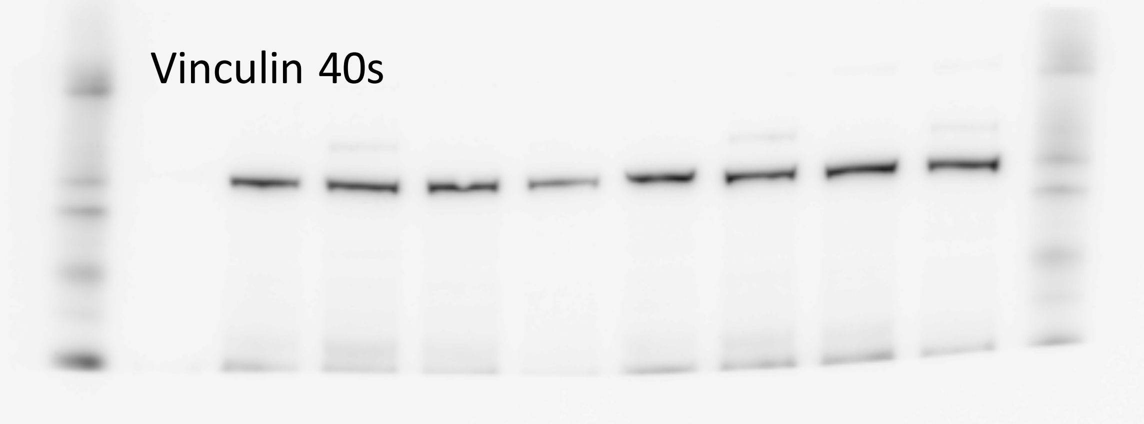

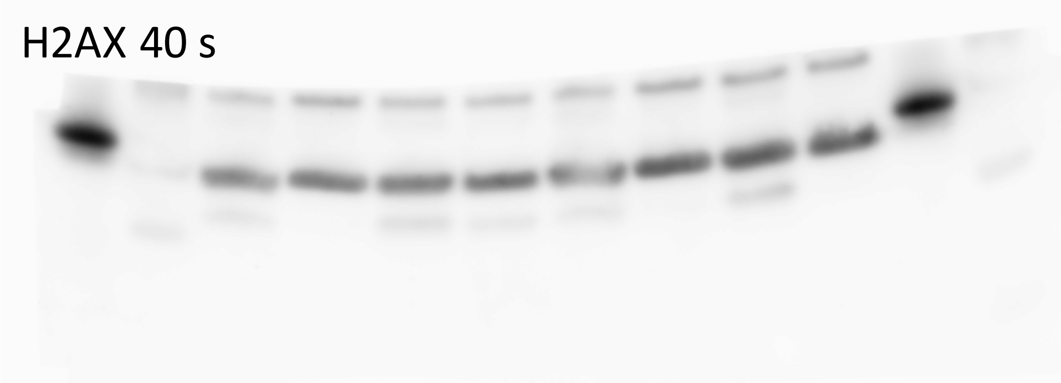

2021-DT-02: IMR-32 cells; PUMA, vinculin; gammaH2AX, H2AX; 72h

Loading:

Laddr	Laddr	48h	48h	48h	48h	72h	72h	72h	72 h	6 d	6 d	6 d	6 d	Laddr
Magic Mark	Visual	Veh	SL-176	GSK-J4	Comb	Veh	SL-176	GSK-J4	Comb	Veh	SL-176	GSK-J4	comb	Magic Mark

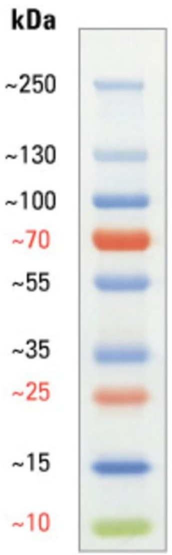

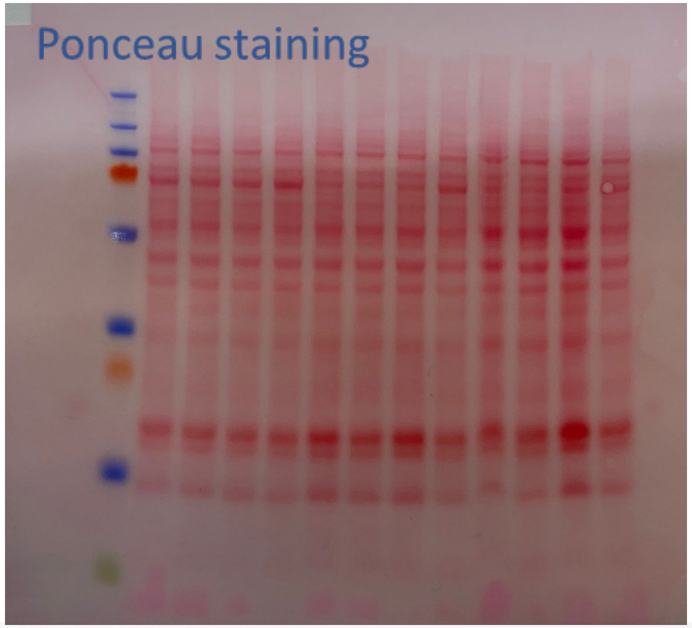

MagicMark

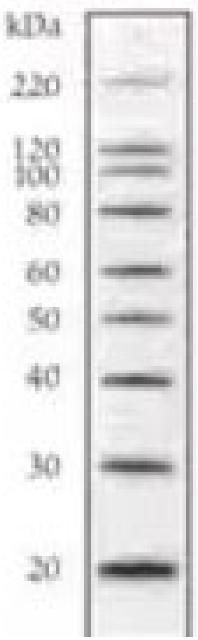

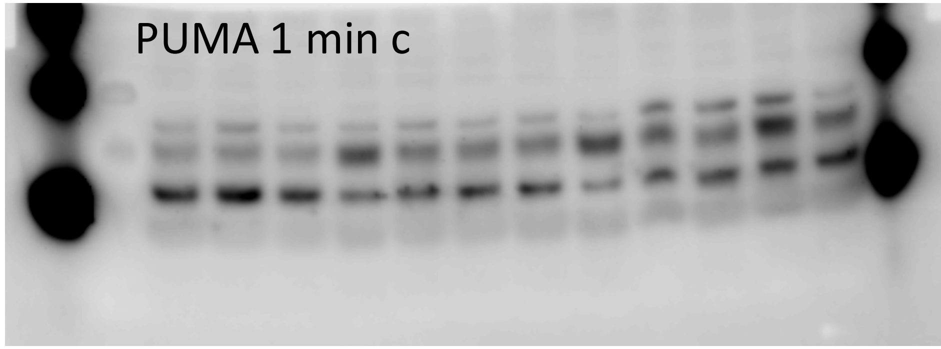

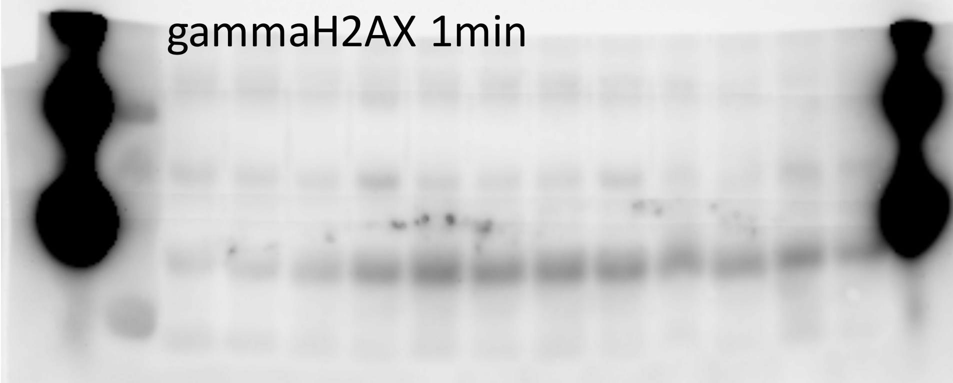

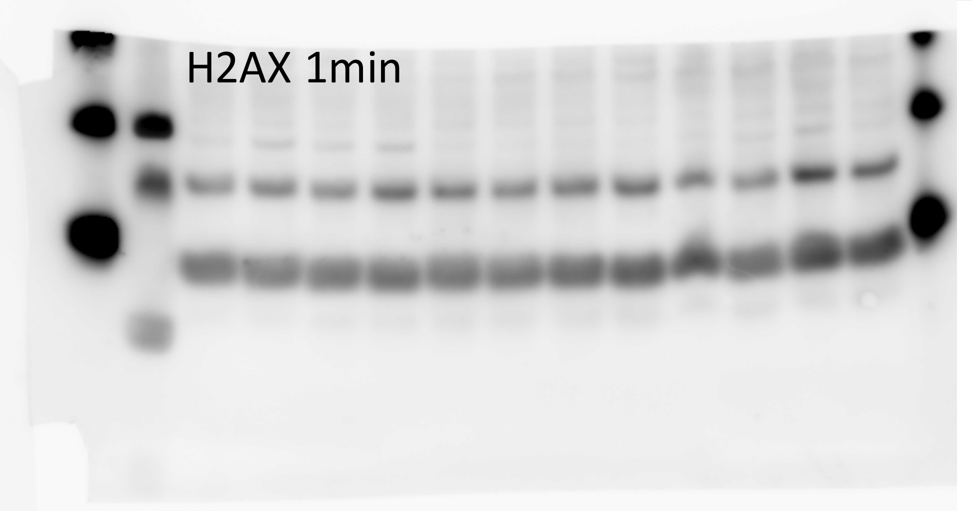

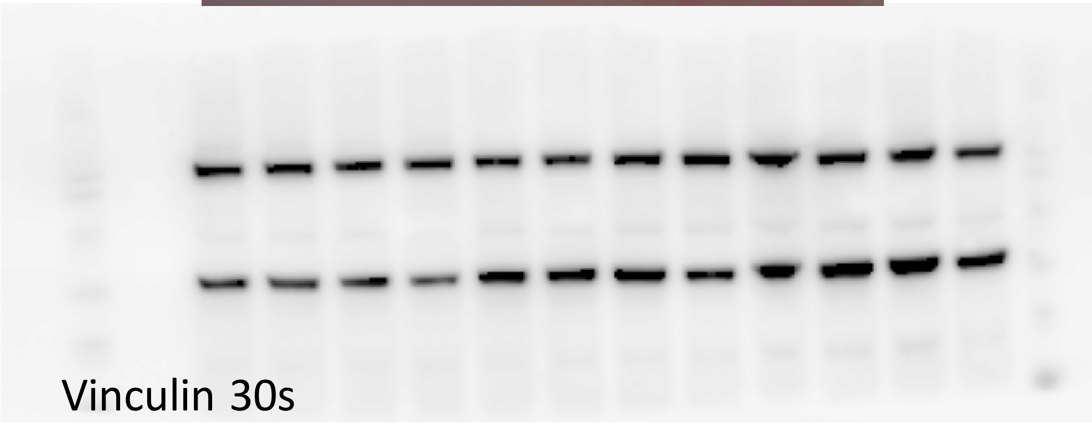

2020-DT-12: IMR-32 cells; WIP1, vinculin; 72h incubation

Loading:

Laddr	Laddr	48h	48h	48h	48h	72h	72h	72h	72 h	6 d	6 d	6 d	6 d	Laddr
Magic Mark	Visual	Veh	SL-176	GSK-J4	Comb	Veh	SL-176	GSK-J4	Comb	Veh	SL-176	GSK-J4	comb	Magic Mark

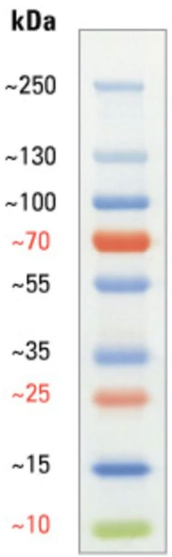

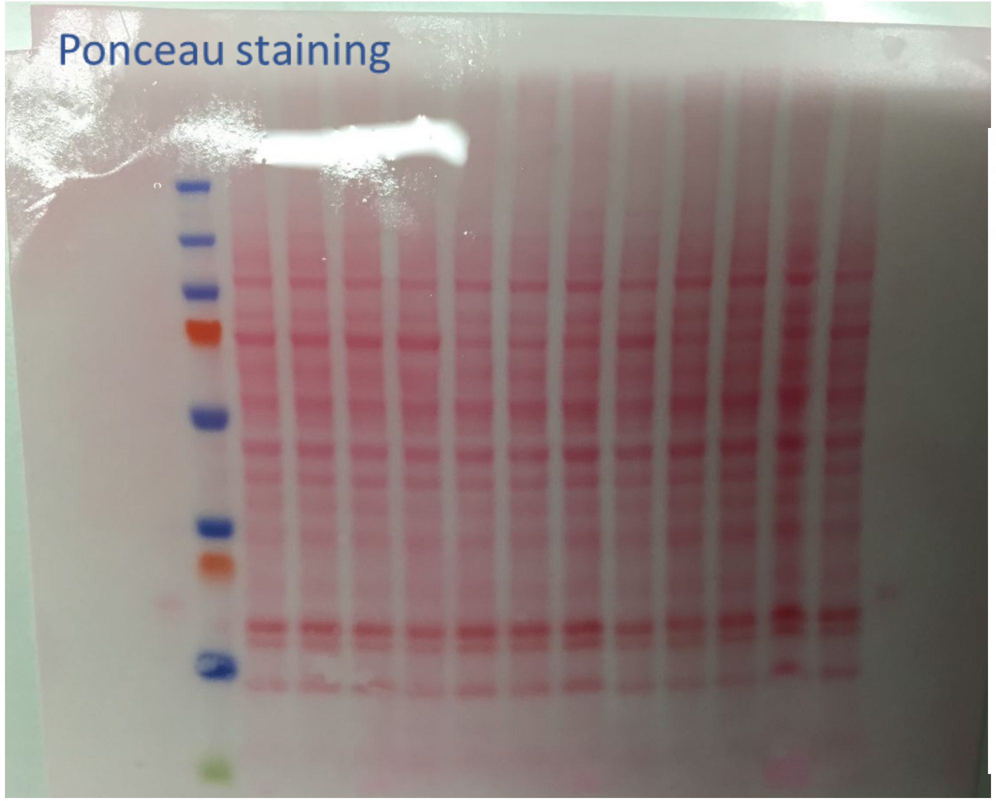

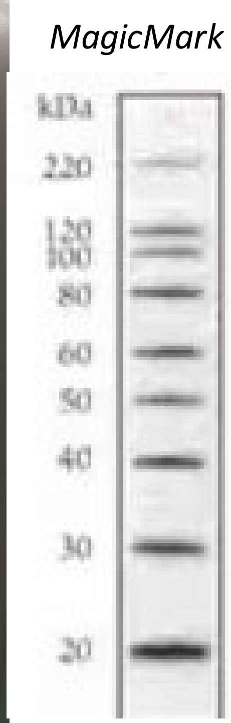

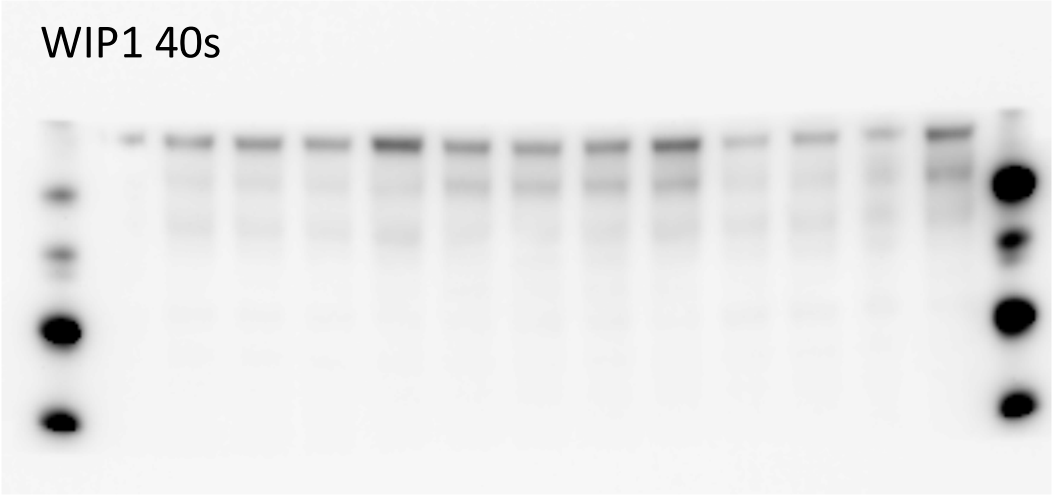

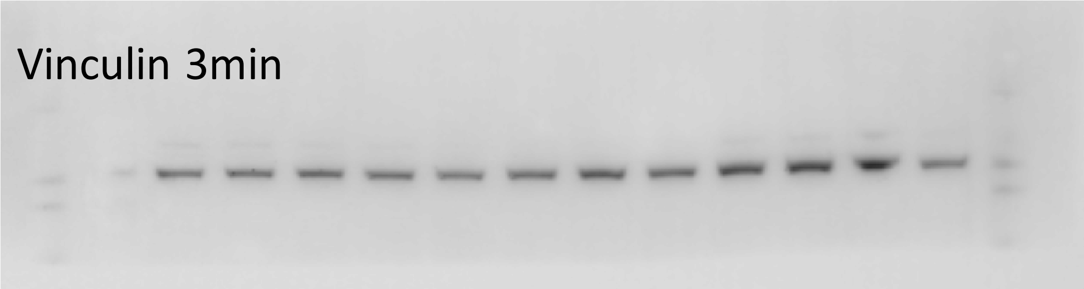

Supplement: Supplementary file 8 — Supplementary Immunoblot Material [file 41419_2025_7658_MOESM8_ESM.pdf]
